# Cyano-borrowing reaction: nickel-catalyzed direct conversion of cyanohydrins and aldehydes/ketones to β-cyano ketone†
†Electronic supplementary information (ESI) available. See DOI: 10.1039/c9sc00640k


**DOI:** 10.1039/c9sc00640k

**Published:** 2019-05-06

**Authors:** Zhao-Feng Li, Qian Li, Li-Qing Ren, Qing-Hua Li, Yun-Gui Peng, Tang-Lin Liu

**Affiliations:** a School of Chemistry and Chemical Engineering , Southwest University , Chongqing 400715 , China . Email: liuschop@swu.edu.cn ; Email: pyg@swu.edu.cn

## Abstract

A direct nickel-catalyzed, high atom- and step-economical reaction of cyanohydrins with aldehydes or ketones *via* an unprecedented “cyano-borrowing reaction” has been developed. Cleavage of the C–CN bond of cyanohydrins followed by aldol condensation and conjugate addition of cyanide to α,β-unsaturated ketones proceeded to deliver a range of racemic β-cyano ketones with good to high yields. The practical procedure with the use of a commercial and less-toxic CN source bodes well for wide application of this protocol.

## Abstract 2

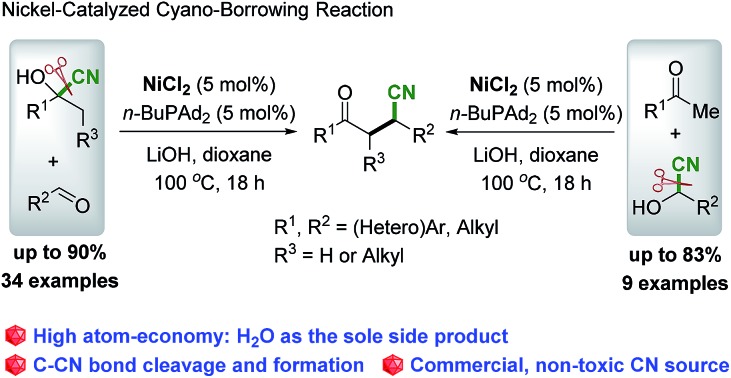
A direct nickel-catalyzed, high atom- and step-economical reaction of cyanohydrins with aldehydes or ketones *via* an unprecedented “cyano-borrowing reaction” has been developed.

## Introduction

**Scheme 1:**
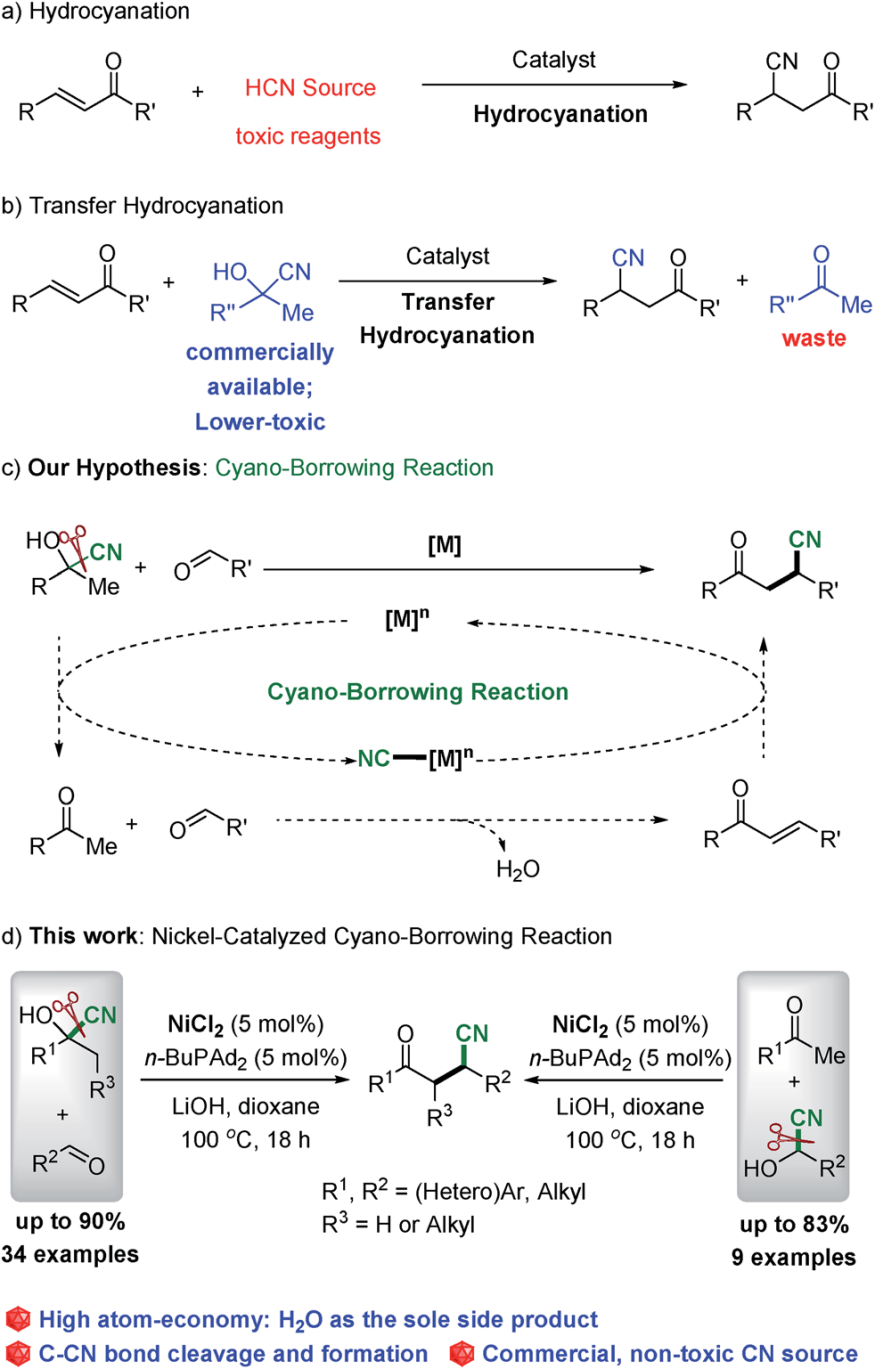
Development of cyano-borrowing.

Nitriles are important synthesis intermediates in transformation^1^ and are key components in various natural products, medicinal pharmacophores and drugs,^2^ and in organic synthesis, the cyano group is equivalent to an amine or carbonyl group. The catalytic addition of the cyano group to the C=C bond has been established as one of the most direct pathways for the synthesis of nitriles.^3^,^4^ Among the important nitriles, β-cyano-ketones are commonly utilized in organic synthesis.^5^ One of the classical approaches to deliver these compounds is the catalyzed conjugate addition of cyanide to α,β-unsaturated carbonyl compounds (hydrocyanation procedure, Scheme 1a),^6^ which utilizes the highly toxic and explosive HCN gas as the cyano source. An alternative strategy is transfer hydrocyanation, which involves the commercially available, less-toxic and less-explosive cyanohydrin to deliver nitriles (Scheme 1b), but with low atom economy.^7^ Recently, Morandi developed a nickel-catalyzed transfer hydrocyanation reaction between alkyl nitriles and alkenes or aryl chlorides, which utilizes non-toxic alkyl nitriles as the cyanide source.^8^ Although the catalyzed hydrocyanation and transfer hydrocyanation reactions have been well developed,^6^,^7^ it remains an important challenge to bypass the usage of toxic HCN gas as the cyano source and overcome the issue of atom-economy. To mitigate these concerns and inspired by the atom- and step-economical procedure of β-alkylation of secondary alcohols with aldehydes *via* borrowing hydrogen reactions,^9^,^10^ we postulated that the cyano group could be tolerated in the reaction with a mechanism analogous to the borrowing hydrogen reactions. Guided by these considerations, we envisioned that, as shown in Scheme 1c, under the catalysis of a transition metal, there is cleavage of the C–CN bond^11^,^12^ of the cyanohydrin and delivery of the corresponding ketones and metal–cyano intermediate ([M]^*n*^–CN), followed by aldol condensation of ketones with aldehydes, and subsequent conjugate addition of cyanide to chalcones and utilization of the [M]^*n*+1^–CN as the cyano donor to deliver the desired products (Scheme 1c). This hypothesis is notable in that cyanohydrin plays a dual role both as the source of ketone and the cyanide donor with high atom- and step-economy. Herein, we report the first catalytic process of the direct transformation of cyanohydrins with aldehydes to deliver β-cyano ketones as the sole product *via* nickel catalyzed cyano-borrowing reactions.^13^

## Results and discussion

To test our hypothesis, we initiated the cyano-borrowing reaction using commercially available acetophenone cyanohydrin **1a** which could be prepared from acetophenone and TMSCN and benzaldehyde **2a** for the optimization of the reaction conditions. After screening an array of transition metal catalysts, we found that nickel complexes showed better performance. To our delight, a cocktail consisting of NiBr_2_, PPh_3_, and LiOH as the base in dioxane at 100 °C could deliver the desired racemic β-cyano ketone **3aa** in 52% yield (Table 1, entry 1). Notably, determined by the crude ^1^H NMR of the reaction mixture, not even a trace of 1,2-addition products was observed.^14^ As shown in Table 1, we then examined an extensive array of parameters. By varying the anion of the nickel salt (*i.e.*, NiCl_2_, Ni(OAc)_2_, Ni(OTf)_2_ and Ni(acac)_2_) as precatalysts, NiCl_2_ exhibited the best yield (Table 1, entries 3–5 *vs.* 2). The screening of different phosphines revealed that this transformation requires a bulky, electron rich ligand, and ^*t*-^BuPAd_2_ showed the highest reactivity with 82% yield (Table 1, entry 11 *vs.* 2 and 6–10). Stronger bases such as NaOH and KO^*t*-^Bu show low reactivity. Meanwhile, Cs_2_CO_3_ and organic bases (*i.e.*, DMAP and DBU) failed to provide the target products (Table 1, entries 14–16). Optimization of the solvent led to no improvement (Table 1, entries 17–19). Lowering the temperature to 80 °C gives a lower yield (Table 1, entry 20). Control experiments verified that the presence of a Ni-complex was necessary to achieve high yield in the cyano-borrowing reaction (Table 1, entry 21). No reaction occurred in the absence of LiOH (Table 1, entry 22). After the screening of the reaction parameters, we found that NiCl_2_ and ^*n*-^BuPAd_2_ as the precatalyst and LiOH as the base in dioxane at 100 °C for 20 h (82% yield) were the optimal conditions.

**Table 1:** Screening studies of β-alkylation of cyanohydrin **1a** with benzaldehyde **2a**^*a*^

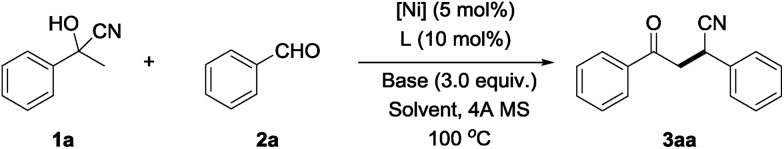					
Entry	[Ni]	**L**	Base	Solvent	Yield^*b*^ (%)
1	NiBr_2_	PPh_3_	LiOH	Dioxane	52
2	NiCl_2_	PPh_3_	LiOH	Dioxane	76
3	Ni(OAc)_2_	PPh_3_	LiOH	Dioxane	66
4	Ni(OTf)_2_	PPh_3_	LiOH	Dioxane	62
5	Ni(acac)_2_	PPh_3_	LiOH	Dioxane	71
6	NiCl_2_	PCy_3_	LiOH	Dioxane	76
7	NiCl_2_	dppe	LiOH	Dioxane	72
8	NiCl_2_	dppp	LiOH	Dioxane	78
9	NiCl_2_	dppb	LiOH	Dioxane	37
10	NiCl_2_	BINAP	LiOH	Dioxane	44
**11**	**NiCl**_**2**_	^***n*-**^ **BuPAd**_**2**_	**LiOH**	**Dioxane**	**82**
12	NiCl_2_	^*n*-^BuPAd_2_	NaOH	Dioxane	28
13	NiCl_2_	^*n*-^BuPAd_2_	KO^*t*-^Bu	Dioxane	Trace
14	NiCl_2_	^*n*-^BuPAd_2_	Cs_2_CO_3_	Dioxane	0
15	NiCl_2_	^*n*-^BuPAd_2_	DMAP	Dioxane	0
16	NiCl_2_	^*n*-^BuPAd_2_	DBU	Dioxane	0
17	NiCl_2_	^*n*-^BuPAd_2_	LiOH	Toluene	22
18	NiCl_2_	^*n*-^BuPAd_2_	LiOH	TBME	23
19	NiCl_2_	^*n*-^BuPAd_2_	LiOH	THF	72
20^*c*^	NiCl_2_	^*n*-^BuPAd_2_	LiOH	Dioxane	59
21	—	—	LiOH	Dioxane	<10
22	NiCl_2_	^*n*-^BuPAd_2_	—	Dioxane	0

^*a*^The reaction was carried out with 0.4 mmol of **1a**, 0.4 mmol of **2a**, 5 mol% [Ni], 10 mol% ligand (**L**) and 300 mol% base in 0.5 mL of solvent at 100 °C for 18 h.

^*b*^Isolated yield.

^*c*^The reaction was carried out at 80 °C. dppm = bis(diphenylphosphino)methane; dppe = bis(diphenylphosphino)ethane; dppp = bis(diphenylphosphino)-propane; dppb = bis(diphenylphosphino)butane; ^*n*-^BuPAd_2_ = di(1-adamantyl)-^*n*-^butylphosphine; TBME = methyl *tert*-butyl ether.

**Scheme 2:**
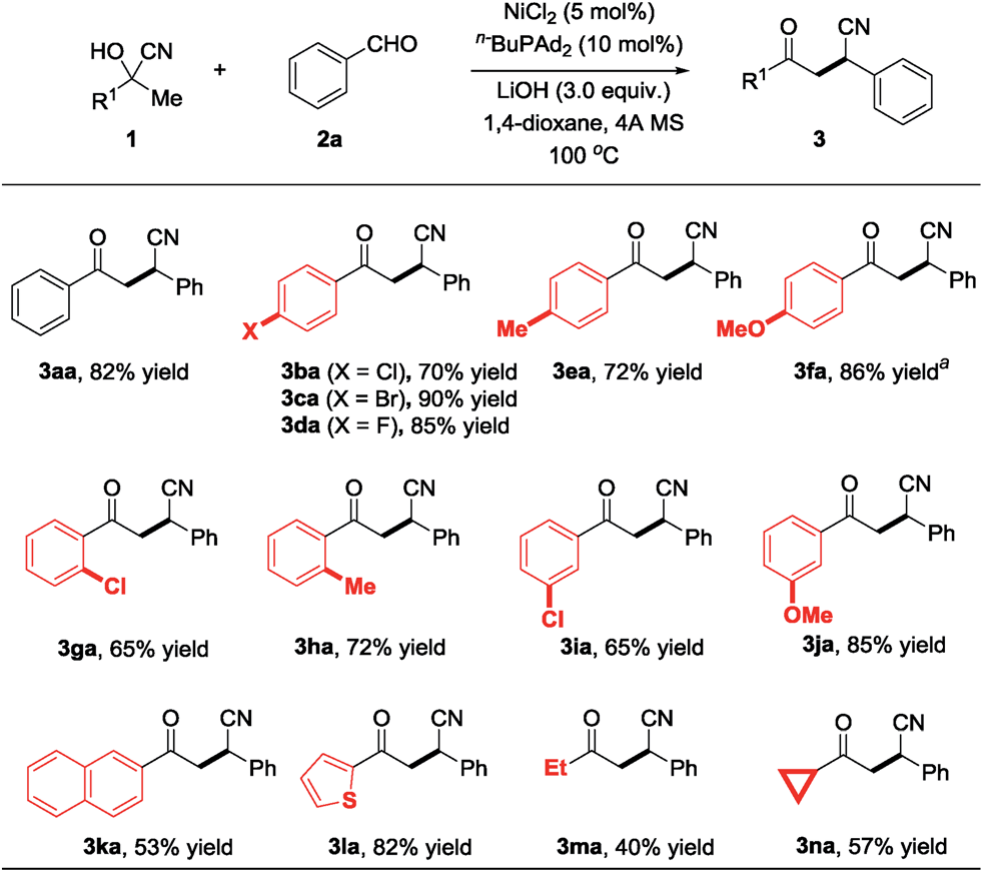
Reaction scope of ketone cyanohydrins. ^a^The reaction was carried out at 120 °C.

Having identified the optimized reaction conditions, we further explored the substrate scope of this reaction. Various commercially available ketone cyanohydrins were examined with benzaldehyde (**2a**) and the results are summarized in Scheme 2. The electron-deficient substrate with varying substituent patterns at the *p*-position did not dramatically influence yields (**3ba–3da**). Substituting the cyanohydrin with *p*-methyl also shows high reactivity and 72% of the desired product was obtained (Scheme 2, **3ea**). However, substituting the *para* position with a more electron-rich functionality (*i.e.*, *p*-MeO) led to diminished yields, but the desired product could be delivered with 86% yield while increasing the reaction temperature to 120 °C (Scheme 2, **3fa**). Reactions of cyanohydrin with *ortho*- and *meta*-substitution on aryl groups gave excellent yields (Scheme 2, **3ga–3ja**). The reaction proceeds smoothly in the case of the cyanohydrin bearing a thienyl group, affording the corresponding product **3la** in 82% isolated yield. In terms of the alkyl substituents, cyanohydrin derived from butanone proceeded smoothly to afford the desired product with 40% yield (Scheme 2, **3ma**). The substrate bearing a cyclopropyl group was well tolerated, leading to the cyclopropyl substituted product with 57% yield (Scheme 2, **3na**). Importantly, the cyclopropyl group remains untouched, which indicates that this nickel-catalyzed protocol does not proceed *via* a radical pathway.

**Scheme 3:**
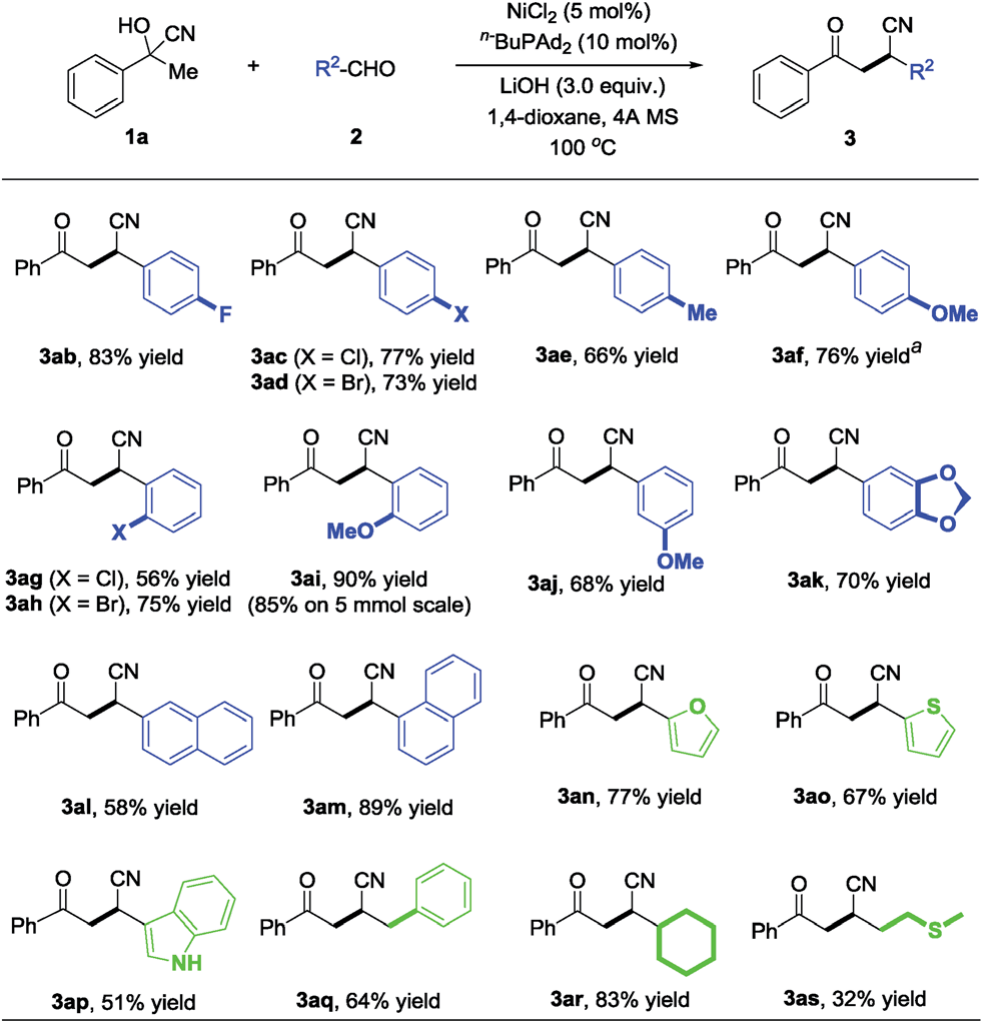
Reaction scope of aldehydes. ^a^The reaction was carried out at 120 °C.

Next, various aldehydes were investigated with acetophenone cyanohydrin (**1a**) using the optimized reaction conditions, and representative results are summarized in Scheme 3. Benzaldehydes bearing various electron-deficient (**3ab–3ad** and **3ag–3ah**), electron-neutral (**3al** and **3am**) and electron-rich (**3ae–3af** and **3ai–3ak**) substituents reacted with **1a** to deliver the desired products in moderate to excellent yields (58–90%). Furthermore, the reaction is also compatible with heteroaryl rings, such as 2-furanyl (**3an**), 2-thiophenyl (**3ao**) and unprotected 3-indolyl (**3ap**), providing diverse β-cyano ketones in 51–77% yield. Remarkably, compared with the aryl aldehydes, the alkyl substituted aldehydes (*e.g.*, 2-phenylacetaldehyde and cyclohexanecarbaldehyde) reacted with **1a** to afford the corresponding products **3aq** and **3ar** in 64% and 80% yields, respectively. The aldehyde containing a sulfur atom is also tolerated under the nickel-catalyzed protocol and delivers the desired product **3as** with a slightly lower yield. To demonstrate the practicality and scalability of our protocol, we proceeded to carry out a gram-scale reaction with 5.0 mmol **2i** reacted with 10.0 mmol **1a** catalyzed by 5 mmol% of NiCl_2_, affording 1.12 g **3ai** in 85% yield, suggesting that this procedure is quite reliable and practically applicable.

**Scheme 4:**
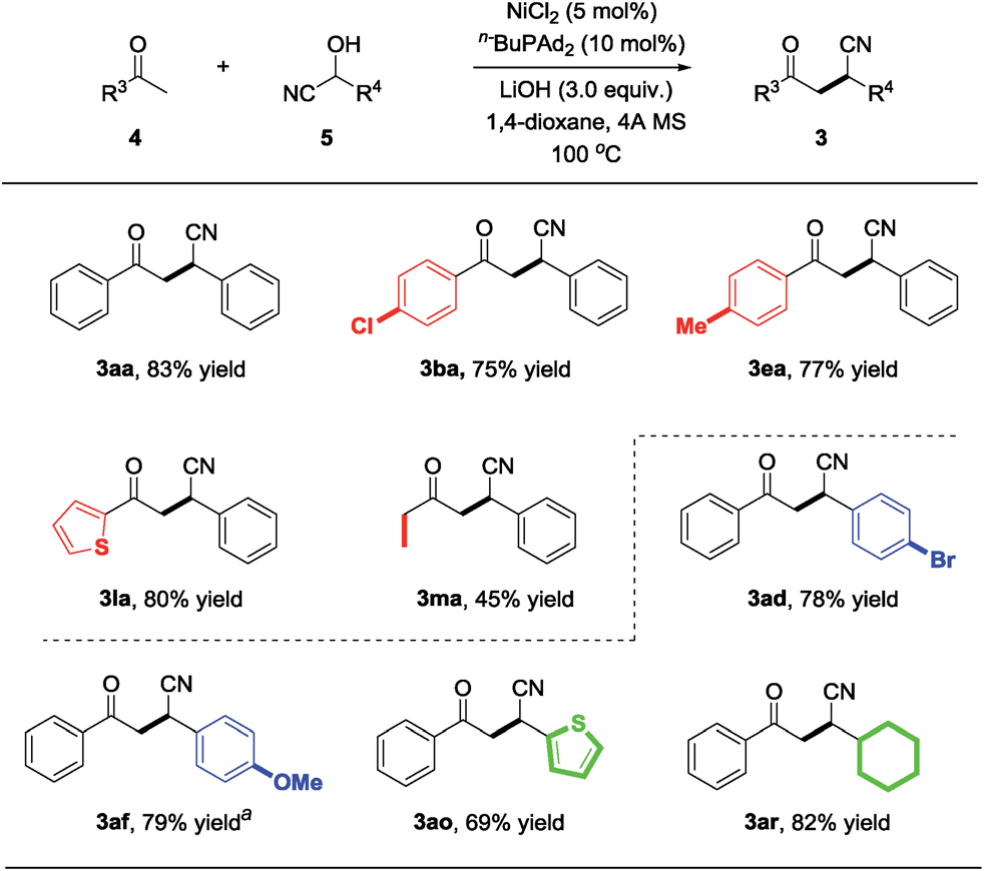
Examples of aldehyde cyanohydrin with ketone. ^a^The reaction was carried out at 120 °C.

Inspired by the success with the nickel-catalyzed cyano-borrowing reaction of cyanohydrins derived from ketones **1** with aldehydes, we further demonstrate the cyano-borrowing protocol with more challenging substrates such as aldehyde cyanohydrin **5**. To our delight, benzaldehyde cyanohydrin **5a** reacted with acetophenone **4a** smoothly under standard conditions, yielding the corresponding product **3aa** with full conversion and 83% isolated yield. To indicate the generality of this protocol, we then examined the scope of benzaldehyde cyanohydrins and ketones. In addition to the phenyl group, it was found that substrates bearing electron-rich or electron-deficient substituents on the benzene ring were well tolerated to give β-cyano ketones in moderate to good yields (**3ba**, **3ea**, **3ad** and **3af** in Scheme 4). Moreover, heteroaryl and alkyl substituents also participated in this protocol very well (Scheme 4, **3la**, **3ma**, **3ao** and **3ar**).

**Scheme 5:**
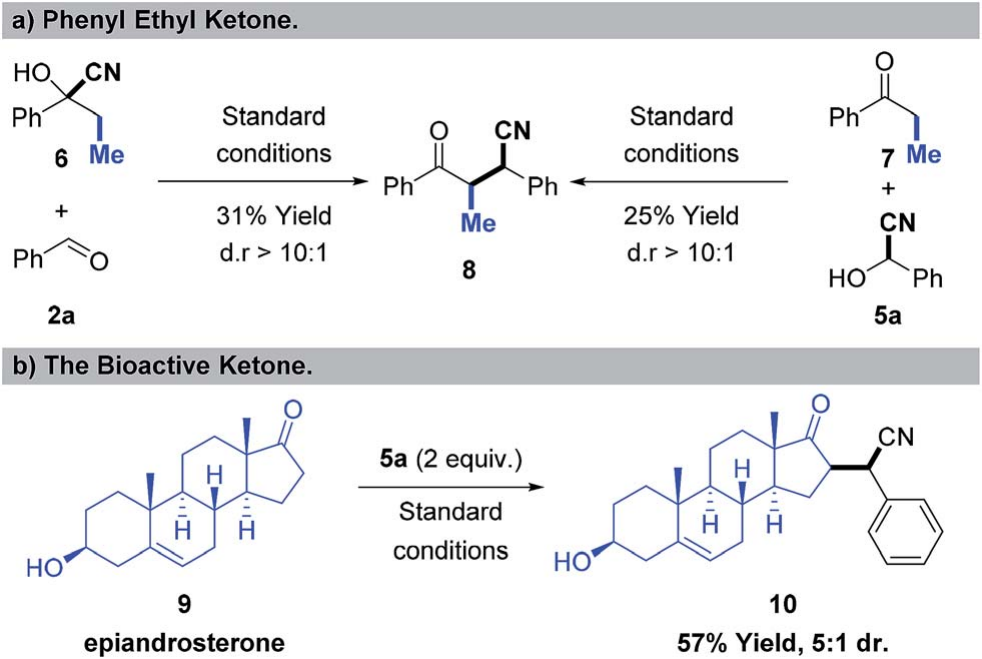
Cyano-borrowing beyond methyl ketones.

To illustrate the scope and limitations of the new transformation, challenging substrates beyond methyl ketones were further examined, shown in Scheme 5. The propiophenone cyanohydrin **6** was selected to react with benzaldehyde **2a** under standard reaction conditions, and the desired product **8** was obtained in 31% isolated yield and with high diastereoselectivity (dr > 10 : 1) (Scheme 5a, left). Furthermore, we also examined benzaldehyde cyanohydrin **5a** and propiophenone **7**, with a nickel catalyst, and the corresponding product **8** was obtained in 25% yield (Scheme 5a, right). Notably, the bioactive ketone, epiandrosterone was tested in this reaction with the partner of benzaldehyde cyanohydrin **5a**, and the corresponding product **10** was delivered in 57% yield with 5 : 1 dr (Scheme 5b), which shows the potential of this nickel-catalyzed cyano borrowing process for the selective modification of bioactive ketones.

**Scheme 6:**
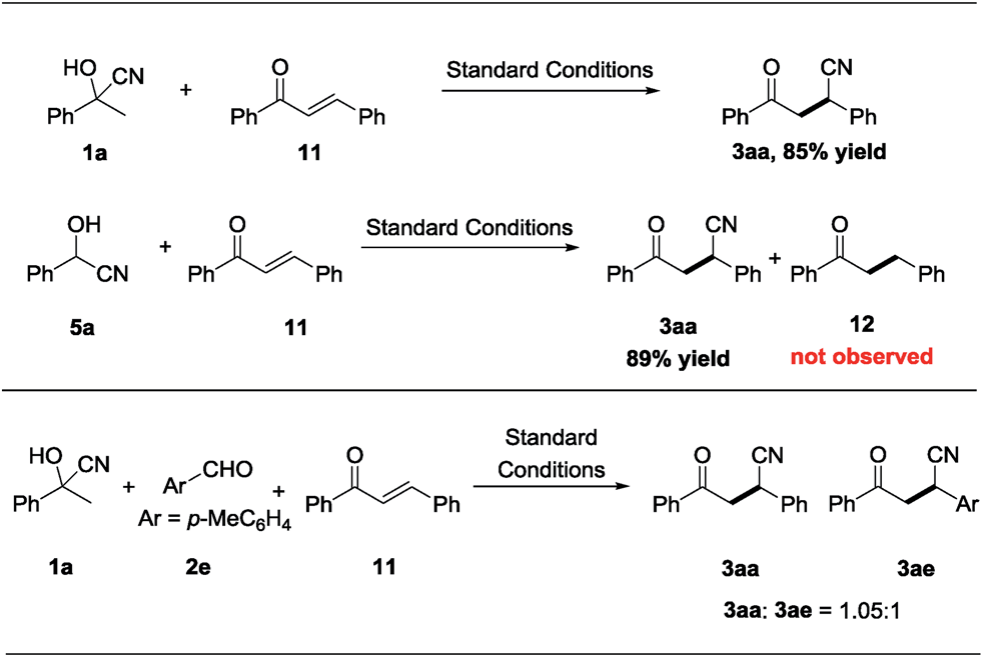
Mechanism studies.

To shed light on the mechanism for the nickel-catalyzed cyano-borrowing protocol, a series of control experiments were conducted. As shown in Scheme 6, acetophenone cyanohydrin **1a** reacted smoothly with chalcone **11** and give the corresponding product **3aa** in excellent yield, which indicated that cyanohydrin is the source of the cyano group in transfer hydrocyanation under standard reaction conditions. Benzaldehyde cyanohydrin **5a** could also react with chalcone **11** efficiently, delivering **3aa** with 89% yield, and the hydrogen-borrowing product **12** was not observed, showing that cleavage of the C–CN bond is easier than that of the C–H bond in cyanohydrins. Meanwhile, in the crossover reaction of **1a**, **2e** and **11** under standard conditions, we got the corresponding products **3aa** and **3ae** with the ratio of 1.05 : 1, which shows that the cyano group from the cleavage of the C–CN bond of cyanohydrin was a free anion in this nickel-catalyzed protocol and has the same opportunity to conjugate to each chalcone. Together, these experimental results support our hypothesis on nickel-catalyzed step- and atom-economical cyano-borrowing reaction of cyanohydrin with aldehydes or ketones (Scheme 1c) (for more details of the mechanism studies, please see the ESI†).

## Experimental

### General procedure

#### Method A

To a vial equipped with a dried stir bar was added aldehydes (0.2 mmol), ketone cyanohydrins (0.4 mmol), NiCl_2_ (5 mol%), ^*n*-^BuPAd_2_ (5 mol%), LiOH (0.6 mmol), 100 mg 4Å MS and anhydrous dioxane (1 mL) in a glovebox. The reaction mixture was taken outside the glovebox and allowed to stir at room temperature for 30 min. After that, the reaction mixture was allowed to stir at 100 °C for 18 hours. The crude reaction mixture was concentrated under reduced pressure and directly purified by silica gel chromatography to give pure products.

#### Method B

To a vial equipped with a dried stir bar was added ketones (0.2 mmol), aldehyde cyanohydrins (0.4 mmol), NiCl_2_ (5 mol%), ^*n*-^BuPAd_2_ (5 mol%), LiOH (0.6 mmol), 100 mg 4Å MS and anhydrous dioxane (1 mL) in a glovebox. The procedure was the same as Method A.

## Conclusions

In conclusion, we have developed an unprecedented nickel-catalyzed protocol for the direct conversion of cyanohydrins and aldehydes or ketones into racemic β-cyano ketones *via* a nickel-catalyzed cyano-borrowing reaction. A range of cyanohydrins derived from aldehydes or ketones could be tolerated and delivered products with high regioselectivity and good to excellent yields. To the best of our knowledge, catalytic conversion of cyanohydrins into β-cyano ketones by reaction with aldehydes/ketones has not been reported. Further studies of catalytic cyano-borrowing reaction of cyanohydrins are in progress in our research lab and will be reported in due course.

## Conflicts of interest

There are no conflicts to declare.

## Supplementary Material

Supplementary information
