# Supplementary material for: Cyano-borrowing reaction: nickel-catalyzed direct conversion of cyanohydrins and aldehydes/ketones to β-cyano ketone
Source: Chem Sci. 2019 May 6;10(22):5787–92. doi: 10.1039/c9sc00640k (PMC6568282; doi:10.1039/c9sc00640k)
Supplement: Supplementary file 1 [file SC-010-C9SC00640K-s001.pdf]

Electronic Supplementary Material (ESI) for Chemical Science.
This journal is © The Royal Society of Chemistry 2019

Supporting Information

Cyano-Borrowing Reaction: Nickel-Catalyzed Direct Conversion of Cyanohydrins and Aldehydes/Ketones to β-Cyano Ketone

Zhao-Feng Li, Qian Li, Li-Qing Ren, Qing-Hua Li, Yun-Gui Peng*, Tang-Lin Liu*

School of Chemistry and Chemical Engineering, Southwest University, Chongqing 400715, China.

Contents:

I General information....................2

II General Procedure for preparation of product from cyanohydrins....................3

III Characterization of products....................3

IV Procedure of gram scale reaction....................17

V The mechanism study....................18

VI References....................23

VII NMR of products....................24

I General information

^{1}**H** and ^{13}**C NMR** spectra were recorded on a Bruker 14A04336 (600 MHz) spectrometer. Chemical shifts were reported in parts per million (ppm), and the residual solvent peak was used as an internal reference: proton (chloroform δ 7.26), carbon (chloroform δ 77.0) or tetramethylsilane (TMS δ 0.00) was used as a reference. Multiplicity was indicated as follows: s (singlet), d (doublet), t (triplet), q (quartet), m (multiplet), dd (doublet of doublet), bs (broad singlet). Coupling constants were reported in Hertz (Hz). All high resolution mass spectra (**HRMS**) were obtained on a micrOTOF-Q II 10269 spectrometer. For thin layer chromatography (**TLC**), TLC plates were used, and compounds were visualized with a UV light at 254 nm. Further visualization was achieved by staining with iodine, or potassium permanganate solution followed by heating using a heat gun. Flash chromatography separations were performed on 300-400 mesh silica gel. The cyanohydrins are commercially available or synthesis *via* the known procedures[1]

II General Procedure for preparation of product from cyanohydrins

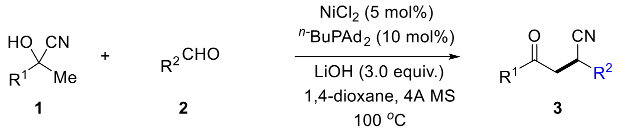

Method A: To a vial equipped with a dried stir bar was added aldehydes (0.2 mmol) ketone cyanohydrins (0.4 mmol) $NiCl_2$ (5 mol%), ligand **L*** (5 mol%), LiOH (0.6 mmol), 100 mg 4Å MS and anhydrous dioxane (1 mL) in the glovebox. The reaction mixture was taken outside the glovebox and allowed to stir at room temperature for 30 min. After then, the reaction mixture was allowed to stir at 100 ^oC for 18 hours. The crude reaction mixture was concentrated under reduced pressure and directly purified by silica gel chromatography to give pure products.

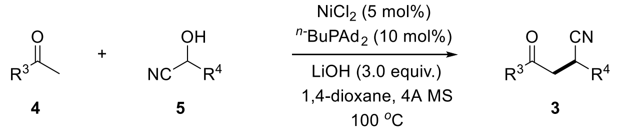

Method B: To a vial equipped with a dried stir bar was added ketones (0.2 mmol) aldehyde cyanohydrins (0.4 mmol) $NiCl_2$ (5 mol%), ligand **L*** (5 mol%), LiOH (0.6 mmol), 100 mg 4Å MS and anhydrous dioxane (1 mL) in the glovebox. The reaction mixture was taken outside the glovebox and allowed to stir at room temperature for 30 min. After then, the reaction mixture was allowed to stir at 100 ^oC for 18 hours. The crude reaction mixture was concentrated under reduced pressure and directly purified by silica gel chromatography to give pure products.

III Characterization of products

4-oxo-2,4-diphenylbutanenitrile (3aa)[2]

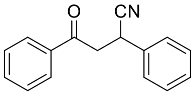

3aa

The title compound was prepared according to the general procedure as described. Silica gel flash column chromatography was performed using hexanes and ethyl acetate (10:1) (R*f* = 0.40 in hexane:ethyl acetate = 5:1) resulting in a white solid in 82% yield (**method A**), 83% yield (**method B**). ^1H NMR (600 MHz, $CDCl_3$) δ 7.91 (d, J = 7.6 Hz, 2H), 7.58 (t, J = 7.1 Hz, 1H), 7.44 (m, 4H), 7.38 (t, J = 7.1 Hz, 2H), 7.32 (t, J = 7.1 Hz, 1H), 4.59-4.52 (m, 1H), 3.71 (dd, J = 17.9 Hz, 8.0 Hz, 1H), 3.50 (dd, J = 17.9 Hz, 5.7 Hz, 1H).

^{13}C NMR (151 MHz, $CDCl_3$) δ 194.66, 135.79, 135.36, 133.87, 129.28, 128.84, 128.38, 128.11, 127.51, 120.63, 44.49, 31.94.

4-(4-chlorophenyl)-4-oxo-2-phenylbutanenitrile (3ba)[2]

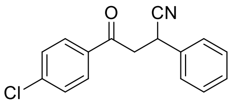

3ba

The title compound was prepared according to the general procedure as described. Silica gel flash column chromatography was performed using hexanes and ethyl acetate (10:1) (R*f* = 0.40 in hexane:ethyl acetate = 5:1) resulting in a white solid in 70% yield (**method A**) and 75% yield (**method B**).

^{1}H NMR (600 MHz, $CDCl_3$) δ 7.78 (d, *J* = 7.7 Hz, 2H), 7.39-7.29 (m, 6H), 7.27 (d, *J* = 5.9 Hz, 1H), 4.47 (t, *J* = 7.6 Hz, 1H), 3.62 (d, *J* = 17.7 Hz, 7.6 Hz, 1H), 3.39 (d, *J* = 17.2 Hz, 1H).

^{13}C NMR (151 MHz, $CDCl_3$) δ 193.49, 140.48, 135.13, 134.09, 130.36–128.85, 128.46, 127.46, 120.42, 44.48, 31.94.

4-(4-bromophenyl)-4-oxo-2-phenylbutanenitrile (3ca)[2]

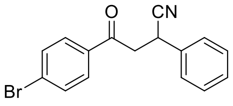

3ca

The title compound was prepared according to the general procedure **A** as described. Silica gel flash column chromatography was performed using hexanes and ethyl acetate (10:1) (R*f* = 0.40 in hexane:ethyl acetate = 5:1) resulting in a white solid in 90% yield.

^{1}H NMR (600 MHz, $CDCl_3$) δ 7.70 (d, *J* = 8.5 Hz, 2H), 7.52 (d, *J* = 8.5 Hz, 2H), 7.36-7.29 (m, 4H), 7.26 (t, *J* = 7.1 Hz, 1H), 4.46 (dd, *J* = 7.7 Hz, 6.1 Hz, 1H), 3.60 (dd, *J* = 17.9 Hz, 8.1 Hz, 1H), 3.38 (dd, *J* = 17.9 Hz, 5.9 Hz, 1H).

^{13}C NMR (151 MHz, $CDCl_3$) δ 193.73, 135.11, 134.47, 132.19, 129.57, 129.33, 129.22, 128.47, 127.47, 120.43, 44.4, 31.93, 29.69.

4-(4-fluorophenyl)-4-oxo-2-phenylbutanenitrile (3da)[3]

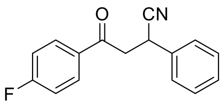

3da

The title compound was prepared according to the general procedure **A** as described. Silica gel flash column chromatography was

performed using hexanes and ethyl acetate (10:1) (R*f* = 0.40 in hexane:ethyl acetate = 5:1) resulting in a white solid in 85% yield.

^{1}H NMR (600 MHz, $CDCl_3$) δ 7.60 (d, *J* = 4.5 Hz, 2H), 7.34 (d, *J* = 7.4 Hz, 2H), 7.30 (t, *J* = 7.4 Hz, 2H), 7.25 (t, *J* = 7.2 Hz, 1H), 7.19 (d, *J* = 12.7 Hz, 1H), 7.04 (t, *J* = 4.1 Hz, 1H), 4.47 (t, *J* = 7.0 Hz, 1H), 3.57 (dd, *J* = 17.3 Hz, 7.8 Hz, 1H), 3.36 (dd, *J* = 17.3 Hz, 6.3 Hz, 1H).

^{13}C NMR (151 MHz, $CDCl_3$) δ 187.42, 142.76, 135.06, 134.73, 132.56, 129.30, 128.40 (d, *J* = 13.7 Hz), 127.49, 120.37, 44.81, 31.99.

4-oxo-2-phenyl-4-(p-tolyl)butanenitrile (3ea)[2]

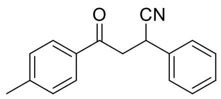

3ea

The title compound was prepared according to the general procedure as described. Silica gel flash column chromatography was performed using hexanes and ethyl acetate (10:1) (R*f* = 0.40 in hexane:ethyl acetate = 5:1) resulting in a white solid in 72% yield (**method A**) and 77% yield (**method B**).

^{1}H NMR (600 MHz, $CDCl_3$) δ 7.74 (d, *J* = 7.1 Hz, 2H), 7.29 (m, 5H), 7.18 (d, *J* = 6.4 Hz, 2H), 4.48 (s, 1H), 3.61 (dd, *J* = 17.7 Hz, 7.8 Hz, 1H), 3.40 (dd, *J* = 17.7 Hz, 3.8 Hz, 1H), 2.33 (s, 3H).

^{13}C NMR (151 MHz, $CDCl_3$) δ 194.22, 144.86, 135.45, 133.37, 129.50, 129.25, 128.33, 128.23, 127.50, 120.67, 44.38, 31.96, 21.66.

4-(4-methoxyphenyl)-4-oxo-2-phenylbutanenitrile (3fa)[3]

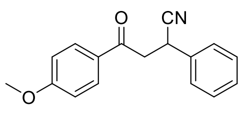

3fa

The title compound was prepared according to the general procedure **A** as described. Silica gel flash column chromatography was performed using hexanes and ethyl acetate (10:1) (R*f* = 0.30 in hexane:ethyl acetate = 5:1) resulting in a white solid in 86% yield.

^{1}H NMR (600 MHz, $CDCl_3$) δ 7.83 (d, *J* = 8.7 Hz, 2H), 7.36 (d, *J* = 7.4 Hz, 2H), 7.32 (t, *J* = 7.5 Hz, 2H), 7.26 (t, *J* = 7.2 Hz, 1H), 6.86 (d, *J* = 8.7 Hz, 2H), 4.50 (t, *J* = 6.9 Hz, 1H), 3.80 (s, 3H), 3.60 (dd, *J* = 17.6 Hz, 7.9 Hz, 1H), 3.38 (dd, *J* = 17.6 Hz, 6.0 Hz, 1H).

^{13}C NMR (151 MHz, $CDCl_3$) δ 193.03, 164.12, 135.49, 130.44, 129.23, 128.88, 128.30, 127.49, 120.72, 114.00, 55.53, 44.16, 32.01.

4-(2-chlorophenyl)-4-oxo-2-phenylbutanenitrile (3ga)[4]

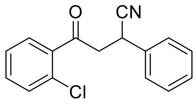

3ga

The title compound was prepared according to the general procedure **A** as described. Silica gel flash column chromatography was performed using hexanes and ethyl acetate (10:1) (R*f* = 0.40 in hexane:ethyl acetate = 5:1) resulting in a white solid in 65% yield.

^{1}H NMR (600 MHz, $CDCl_3$) δ 7.40 (d, *J* = 7.4 Hz, 1H), 7.35-7.28 (m, 6H), 7.28 – 7.22 (m, 2H), 4.46 (t, *J* = 7.1 Hz, 1H), 3.63 (dd, *J* = 17.9 Hz, 8.0 Hz, 1H), 3.46 (dd, *J* = 17.9 Hz, 6.3 Hz, 1H).

^{13}C NMR (151 MHz, $CDCl_3$) δ 197.53, 137.67, 134.87, 132.67, 131.36, 130.77, 129.54 , 129.28, 128.46, 127.51, 127.19, 120.23, 48.30, 32.26.

4-oxo-2-phenyl-4-(o-tolyl)butanenitrile (3ha)

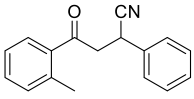

3ha

The title compound was prepared according to the general procedure **A** as described. Silica gel flash column chromatography was performed using hexanes and ethyl acetate (10:1) (R*f* = 0.40 in hexane:ethyl acetate = 5:1) resulting in a white solid in 72% yield.

^{1}H NMR (600 MHz, $CDCl_3$) δ 7.48 (t, *J* = 10.3 Hz, 1H), 7.31 (m, 5H), 7.25 (t, *J* = 7.1 Hz, 1H), 7.16 (t, *J* = 8.1 Hz, 2H), 4.46 (t, *J* = 7.1 Hz, 1H), 3.55 (dd, *J* = 17.6 Hz, 8.0 Hz, 1H), 3.36 (dd, *J* = 17.6 Hz, 6.2 Hz, 1H), 2.41 (s, 3H).

^{13}C NMR (151 MHz, $CDCl_3$) δ 198.02, 139.05, 136.20, 135.29, 132.32, 132.18, 129.26, 128.57, 128.37, 127.50, 125.87, 120.62, 46.86, 32.22, 21.39.

HRMS (ESI): m/z Calcd. for $[C_{17}H_{15}NO, M+H]^+$: 272.1046; Found: 272.1047.

4-(3-chlorophenyl)-4-oxo-2-phenylbutanenitrile (3ia)[4]

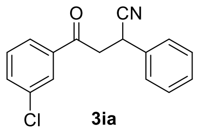

3ia

The title compound was prepared according to the general procedure **A** as described. Silica gel flash column chromatography was performed using hexanes and ethyl acetate (10:1) (R*f* = 0.40 in hexane:ethyl acetate = 5:1) resulting in a white solid in 65% yield

^{1}H NMR (600 MHz, $CDCl_3$) δ 7.81 (s, 1H), 7.71 (d, *J* = 7.3 Hz, 1H), 7.48 (d, *J* = 7.4 Hz, 1H), 7.38–7.29 (m, 5H), 7.27 (d, *J* = 5.9 Hz, 1H), 4.46 (s, 1H), 3.62 (dd, *J* = 17.6 Hz, 7.4 Hz, 1H), 3.40 (dd, *J* = 17.8 Hz, 2.9 Hz, 1H).

^{13}C NMR (151 MHz, $CDCl_3$) δ 193.47 , 137.24, 135.28, 135.05, 133.81, 130.18, 129.34, 128.50, 128.23, 127.47, 126.15, 120.34, 44.62, 31.90.

4-(3-methoxyphenyl)-4-oxo-2-phenylbutanenitrile (3ja)

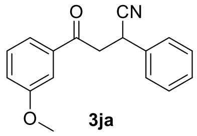

3ja

The title compound was prepared according to the general procedure **A** as described. Silica gel flash column chromatography was performed using hexanes and ethyl acetate (10:1) (R*f* = 0.40 in hexane:ethyl acetate = 5:1) resulting in a white solid in 85% yield.

^{1}H NMR (600 MHz, $CDCl_3$) δ 7.40-7.24 (m, 8H), 7.04 (d, *J* = 7.7 Hz, 1H), 4.46 (t, *J* = 6.7 Hz, 1H), 3.75 (s, 3H), 3.62 (dd, *J* = 17.9 Hz, 8.0 Hz, 1H), 3.41 (dd, *J* = 17.9 Hz, 5.8 Hz, 1H).

^{13}C NMR (151 MHz, $CDCl_3$) δ 193.49 , 159.02 , 136.11 , 134.31 , 128.78 , 128.25 , 127.35 , 126.47 , 119.59 (d, *J* = 9.4 Hz), 119.36 , 111.41 , 54.47 , 43.54 , 30.97 .

HRMS (ESI): m/z Calcd. for $[C_{17}H_{15}NO_2, M+H]^+$: 288.0995; Found: 288.1000.

4-(naphthalen-2-yl)-4-oxo-2-phenylbutanenitrile (3ka)[2]

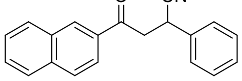

3ka

The title compound was prepared according to the general procedure **A** as described. Silica gel flash column chromatography was performed using hexanes and ethyl acetate (10:1) (*Rf* = 0.40 in hexane:ethyl acetate = 5:1) resulting in a white solid in 53% yield.

^{1}H NMR (600 MHz, $CDCl_3$) δ 8.32 (s, 1H), 7.90 (d, *J* = 8.6 Hz, 1H), 7.84 (d, *J* = 8.2 Hz, 1H), 7.79 (dd, *J* = 11.8, 8.6 Hz, 2H), 7.53 (t, *J* = 7.4 Hz, 1H), 7.47 (t, *J* = 7.5 Hz, 1H), 7.39 (d, *J* = 7.4 Hz, 2H), 7.32 (t, *J* = 7.6 Hz, 2H), 7.26 (t, *J* = 7.4 Hz, 1H), 4.54

(dd, J = 7.7 Hz, 6.2 Hz, 1H), 3.77 (dd, J = 17.7 Hz, 8.0 Hz, 1H), 3.56 (dd, J = 17.7 Hz, 6.0 Hz, 1H).

^{13}C NMR (151 MHz, $CDCl_3$) δ 194.56, 135.90, 135.40, 133.12, 132.42, 130.03, 129.61, 129.31, 128.94, 128.79, 128.40, 127.86, 127.55, 127.08, 123.50, 120.67, 44.59 , 32.07.

4-oxo-2-phenyl-4-(thiophen-2-yl)butanenitrile (3la)[2]

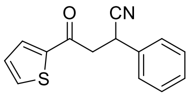

3la

The title compound was prepared according to the general procedure as described. Silica gel flash column chromatography was performed using hexanes and ethyl acetate (10:1) (R*f* = 0.40 in hexane:ethyl acetate = 5:1) resulting in a white solid in 82% yield(**method A**), 80% yield (**method B**).

^{1}H NMR (600 MHz, $CDCl_3$) δ 7.60 (t, J = 3.9 Hz, 2H), 7.34 (d, J = 7.3 Hz, 2H), 7.30 (t, J = 7.5 Hz, 2H), 7.25 (t, J = 7.2 Hz, 1H), 7.07–7.02 (m, 1H), 4.47 (t, J = 7.0 Hz, 1H), 3.56 (dd, J = 17.3 Hz, 7.8 Hz, 1H), 3.36 (dd, J = 17.3 Hz, 6.3 Hz, 1H).

^{13}C NMR (151 MHz, $CDCl_3$) δ 197.44, 166.20, 140.29, 133.93, 129.82, 128.18, 52.40 , 26.80.

4-oxo-2-phenylhexanenitrile (3ma)

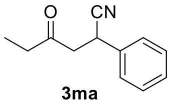

3ma

The title compound was prepared according to the general procedure as described. Silica gel flash column chromatography was performed using hexanes and ethyl acetate (10:1) (R*f* = 0.40 in hexane:ethyl acetate = 5:1) resulting in a white solid in 40% yield(**method A**), 45% yield (**method B**).

^{1}H NMR (600 MHz, $CDCl_3$) δ 7.32–7.24 (m, 5H), 4.30 (dd, J = 7.5 Hz, 6.6 Hz, 1H), 3.08 (dd, J = 17.7 Hz, 7.8 Hz, 1H), 2.86 (dd, J = 17.7 Hz, 6.3 Hz, 1H), 2.40 (dq, J = 17.8 Hz, 7.3 Hz, 1H), 2.30 (dq, J = 17.8 Hz, 7.3 Hz, 1H), 0.99 (t, J = 7.3 Hz, 3H).

^{13}C NMR (151 MHz, $CDCl_3$) δ 205.89, 135.16, 129.24, 128.35, 127.36, 120.43, 47.53, 36.18, 31.69, 7.46.

HRMS (ESI): m/z Calcd. for $[C_{12}H_{13}NO, M+H]^+$: 210.0889; Found: 210.0892.

4-cyclopropyl-4-oxo-2-phenylbutanenitrile (3na)

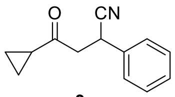

3na

The title compound was prepared according to the general procedure **A** as described. Silica gel flash column chromatography was performed using hexanes and ethyl acetate (10:1) (R*f* = 0.40 in hexane:ethyl acetate = 5:1) resulting in a white solid in 57% yield.

^{1}H NMR (600 MHz, $CDCl_3$) δ 7.33-7.24 (m, 5H), 4.30 (dd, *J* = 7.9 Hz, 6.2 Hz, 1H), 3.24 (dd, *J* = 17.7 Hz, 7.9 Hz, 1H), 3.02 (dd, *J* = 17.7 Hz, 6.1 Hz, 1H), 1.81 (tt, *J* = 7.8 Hz, 4.5 Hz, 1H), 1.05 (m, 1H), 0.98 (m, 1H), 0.86 (m, 2H).

^{13}C NMR (151 MHz, $CDCl_3$) δ 205.24, 135.23, 129.19, 128.28, 127.35, 120.44, 48.47, 31.73, 20.77, 11.43, 11.32.

HRMS (ESI): m/z Calcd. for $[C_{13}H_{13}NO, M+H]^+$: 222.0889; Found: 222.0890.

2-(4-fluorophenyl)-4-oxo-4-phenylbutanenitrile (3ab)[2]

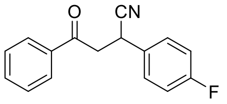

3ab

The title compound was prepared according to the general procedure **A** as described. Silica gel flash column chromatography was performed using hexanes and ethyl acetate (10:1) (R*f* = 0.30 in hexane : ethyl acetate = 5:1) resulting in a white solid in 83% yield.

^{1}H NMR (600 MHz, $CDCl_3$) δ 7.84 (d, *J* = 7.4 Hz, 2H), 7.52 (t, *J* = 7.4 Hz, 1H), 7.39 (t, *J* = 7.8 Hz, 2H), 7.34 (dd, *J* = 8.6 Hz, 5.1 Hz, 2H), 7.00 (t, *J* = 8.6 Hz, 2H), 4.49 (t, *J* = 6.9 Hz, 1H), 3.63 (dd, *J* = 17.9 Hz, 7.4 Hz, 1H), 3.43 (dd, *J* = 17.9 Hz, 6.4 Hz, 1H).

^{13}C NMR (151 MHz, $CDCl_3$) δ 194.44, 163.37, 161.73, 135.68, 133.97, 131.12 (d, *J* = 3.2 Hz), 129.34 (d, *J* = 8.3 Hz), 128.87, 128.09, 44.43, 31.22.

2-(4-chlorophenyl)-4-oxo-4-phenylbutanenitrile (3ac)[2]

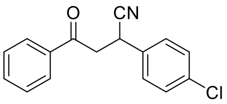

3ac

The title compound was prepared according to the general procedure **A** as described. Silica gel flash column chromatography was performed using hexanes and ethyl acetate (10:1) (R*f* = 0.40 in hexane:ethyl acetate =5:1) resulting in a white solid in 77% yield.

^{1}H NMR (600 MHz, $CDCl_3$) δ 7.84 (dd, *J* = 8.3, 1.1 Hz, 2H), 7.53 (t, *J* = 7.4 Hz, 1H), 7.40 (t, *J* = 7.8 Hz, 2H), 7.33–7.26 (m, 4H), 4.49 (t, *J* = 6.9 Hz, 1H), 3.63 (dd, *J* = 17.9 Hz, 7.4 Hz, 1H), 3.43 (dd, *J* = 17.9 Hz, 6.4 Hz, 1H).

^{13}C NMR (151 MHz, $CDCl_3$) δ 194.32, 135.62, 134.47, 134.01, 133.82, 129.45, 128.96, 128.88, 128.09, 44.27, 31.34.

2-(4-bromophenyl)-4-oxo-4-phenylbutanenitrile (3ad)[2]

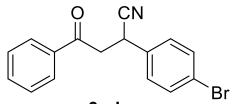

3ad

The title compound was prepared according to the general procedure as described. Silica gel flash column chromatography was performed using hexanes and ethyl acetate (10:1) (R*f* = 0.40 in hexane:ethyl acetate = 5:1) resulting in a white solid in 73% yield (**method A**), 78% yield (**method B**).

^{1}H NMR (600 MHz, $CDCl_3$) δ 7.83 (d, *J* = 7.5 Hz, 2H), 7.52 (t, *J* = 7.3 Hz, 1H), 7.43 (d, *J* = 8.2 Hz, 2H), 7.39 (t, *J* = 7.6 Hz, 2H), 7.24 (d, *J* = 8.1 Hz, 2H), 4.46 (t, *J* = 6.8 Hz, 1H), 3.62 (dd, *J* = 17.9 Hz, 7.4 Hz, 1H), 3.42 (dd, *J* = 17.9 Hz, 6.4 Hz, 1H).

^{13}C NMR (151 MHz, $CDCl_3$) δ 194.32, 135.62, 134.37, 134.00, 132.41, 129.28, 128.88, 128.09, 122.50, 120.15, 44.19, 31.42.

4-oxo-4-phenyl-2-(p-tolyl)butanenitrile (3ae)[2]

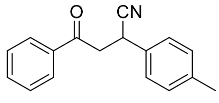

3ae

The title compound was prepared according to the general procedure **A** as described. Silica gel flash column chromatography was performed using hexanes and ethyl acetate (10:1) (R*f* = 0.40 in hexane:ethyl acetate = 5:1) resulting in a white solid in 66% yield.

^{1}H NMR (600 MHz, $CDCl_3$) δ 7.84 (d, *J* = 7.6 Hz, 2H), 7.50 (t, *J* = 7.4 Hz, 1H), 7.38 (t, *J* = 7.7 Hz, 2H), 7.23 (d, *J* = 7.9 Hz, 2H), 7.11 (d, *J* = 7.8 Hz, 2H), 4.48–4.39 (m, 1H), 3.62 (dd, *J* = 17.9 Hz, 7.9 Hz, 1H), 3.40 (dd, *J* = 17.9 Hz, 6.0 Hz, 1H), 2.26 (s, 3H).

^{13}C NMR (151 MHz, $CDCl_3$) δ 194.74 , 138.24, 135.84, 133.82, 132.32, 129.91, 128.82, 128.10, 127.36, 44.53, 31.56, 21.04.

2-(4-methoxyphenyl)-4-oxo-4-phenylbutanenitrile (3af)[2]

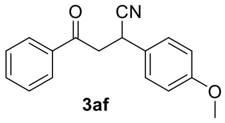

3af

The title compound was prepared according to the general procedure as described. Silica gel flash column chromatography was performed using hexanes and ethyl acetate (10:1) (R*f* = 0.30 in hexane:ethyl acetate = 5:1) resulting in a white solid in 76% yield (**method A**), 79% yield (**method B**).

^{1}H NMR (600 MHz, $CDCl_3$) δ 7.85 (d, *J* = 7.7 Hz, 2H), 7.52 (t, *J* = 7.2 Hz, 1H), 7.39 (t, *J* = 7.4 Hz, 2H), 7.27 (d, *J* = 7.4 Hz, 2H), 6.83 (d, *J* = 7.4 Hz, 2H), 4.45 (t, *J* = 6.7 Hz, 1H), 3.73 (s, 3H), 3.62 (dd, *J* = 17.8 Hz, 7.6 Hz, 1H), 3.42 (dd, *J* = 17.8 Hz, 6.2 Hz, 1H).

^{13}C NMR (151 MHz, $CDCl_3$) δ 194.76, 159.59, 135.85, 133.82, 128.81, 128.66, 128.08, 127.24, 120.85, 114.65, 55.36, 44.56, 31.17.

2-(2-chlorophenyl)-4-oxo-4-phenylbutanenitrile (3ag)[2]

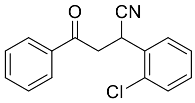

3ag

The title compound was prepared according to the general procedure **A** as described. Silica gel flash column chromatography was performed using hexanes and ethyl acetate (10:1) (R*f* = 0.40 in hexane:ethyl acetate = 5:1) resulting in a white solid in 56% yield.

^{1}H NMR (600 MHz, $CDCl_3$) δ 7.87 (d, *J* = 7.8 Hz, 2H), 7.61 (d, *J* = 7.5 Hz, 1H), 7.53 (t, *J* = 7.2 Hz, 1H), 7.40 (t, *J* = 7.5 Hz, 2H), 7.36 (d, *J* = 7.8 Hz, 1H), 7.26 (dt, *J* = 23.7 Hz, 7.3 Hz, 2H), 4.85 (d, *J* = 9.3, 1H), 3.60 (dd, *J* = 17.9 Hz, 9.4 Hz, 1H), 3.46 (dd, *J* = 17.9 Hz, 4.0 Hz, 1H).

^{13}C NMR (151 MHz, $CDCl_3$) δ 194.41, 135.67, 133.89, 132.75, 130.31, 129.88, 129.49, 128.84, 128.12, 127.77, 119.65, 42.42, 30.07.

2-(2-bromophenyl)-4-oxo-4-phenylbutanenitrile (3ah)[4]

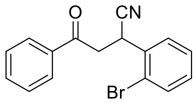

3ah

The title compound was prepared according to the general procedure **A** as described. Silica gel flash column chromatography was performed using hexanes and ethyl acetate (10:1) (R*f* = 0.40 in hexane:ethyl acetate = 5:1) resulting in a white solid in 75% yield.

^{1}H NMR (600 MHz, $CDCl_3$) δ 7.87 (d, *J* = 7.3 Hz, 2H), 7.62 (d, *J* = 7.7 Hz, 1H), 7.53 (t, *J* = 8.4 Hz, 2H), 7.40 (t, *J* = 7.4 Hz, 2H), 7.33 (t, *J* = 7.5 Hz, 1H), 7.16 (dd, *J* = 13.8 Hz, 6.3 Hz, 1H), 4.85 (dd, *J* = 9.2 Hz, 3.6 Hz, 1H), 3.58 (dd, *J* = 17.9 Hz, 9.7 Hz, 1H), 3.45 (dd, *J* = 17.9 Hz, 3.1 Hz, 1H).

^{13}C NMR (151 MHz, $CDCl_3$) δ 194.35, 135.66, 134.46, 133.89, 133.64, 130.08, 129.50, 128.84, 128.42, 128.13, 122.87, 119.72, 42.73, 32.49.

2-(2-methoxyphenyl)-4-oxo-4-phenylbutanenitrile (3ai)[2]

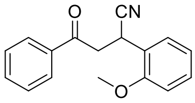

3ai

The title compound was prepared according to the general procedure **A** as described. Silica gel flash column chromatography was performed using hexanes and ethyl acetate (10:1) (R*f* = 0.30 in hexane:ethyl acetate = 5:1) resulting in a white solid in 90% yield.

^{1}H NMR (600 MHz, $CDCl_3$) δ 7.86 (d, *J* = 7.6 Hz, 2H), 7.50 (t, *J* = 7.3 Hz, 1H), 7.42 (d, *J* = 7.5 Hz, 1H), 7.38 (t, *J* = 7.6 Hz, 2H), 7.25 (t, *J* = 7.7 Hz, 1H), 6.92 (t, *J* = 7.4 Hz, 1H), 6.84 (d, *J* = 8.2 Hz, 1H), 4.69 (dd, *J* = 8.9 Hz, 4.7 Hz, 1H), 3.78 (s, 3H), 3.57 (dd, *J* = 17.8 Hz, 9.1 Hz, 1H), 3.42 (dd, *J* = 17.8 Hz, 4.6 Hz, 1H).

^{13}C NMR (151 MHz, $CDCl_3$) δ 195.33, 156.31, 136.00, 133.65, 129.78, 128.95, 128.75, 128.09, 123.24, 121.11, 120.54, 111.10, 55.59, 42.19, 27.48.

2-(3-methoxyphenyl)-4-oxo-4-phenylbutanenitrile (3aj)[3]

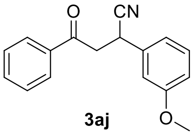

3aj

The title compound was prepared according to the general procedure **A** as described. Silica gel flash column chromatography was performed using hexanes and ethyl acetate (10:1) (R*f* = 0.30 in hexane:ethyl acetate = 5:1) resulting in a white solid in 68% yield.

^{1}H NMR (600 MHz, $CDCl_3$) δ 7.84 (d, *J* = 7.6 Hz, 2H), 7.51 (t, *J* = 7.3 Hz, 1H), 7.38 (t, *J* = 7.7 Hz, 2H), 7.22 (t, *J* = 7.9 Hz, 1H), 6.92 (d, *J* = 7.5 Hz, 1H), 6.88 (s, 1H), 6.78 (dd, *J* = 8.2 Hz, 1.7 Hz, 1H), 4.45 (dd, *J* = 7.8 Hz, 6.0 Hz, 1H), 3.73 (s, 3H), 3.64 (dd, *J* = 17.9 Hz, 8.1 Hz, 1H), 3.42 (dd, *J* = 17.9 Hz, 5.8 Hz, 1H).

^{13}C NMR (151 MHz, $CDCl_3$) δ 194.65, 160.23, 136.76, 135.78, 133.87, 130.35, 128.83, 128.11, 120.52, 119.64, 113.82, 113.35, 55.37, 44.47, 31.93.

2-(benzo[d][1,3]dioxol-5-yl)-4-oxo-4-phenylbutanenitrile (3ak)[2]

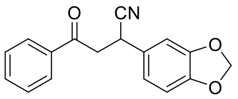

3ak

The title compound was prepared according to the general procedure **A** as described. Silica gel flash column chromatography was performed using hexanes and ethyl acetate (10:1) (R*f* = 0.30 in hexane:ethyl acetate = 5:1) resulting in a white solid in 70% yield.

^{1}H NMR (600 MHz, $CDCl_3$) δ 7.89-7.82 (m, 2H), 7.52 (t, *J* = 7.4 Hz, 1H), 7.40 (t, *J* = 7.8 Hz, 2H), 6.84-6.79 (m, 2H), 6.72 (d, *J* = 7.9 Hz, 1H), 5.89 (s, 2H), 4.41 (t, *J* = 6.9 Hz, 1H), 3.60 (dd, *J* = 17.8 Hz, 7.6 Hz, 1H), 3.41 (dd, *J* = 17.8 Hz, 6.3 Hz, 1H).

^{13}C NMR (151 MHz, $CDCl_3$) δ 194.63, 148.35, 147.70, 135.76, 133.89, 128.84, 128.10, 121.02, 120.68, 108.77, 107.96, 101.46, 44.54, 31.59.

2-(naphthalen-2-yl)-4-oxo-4-phenylbutanenitrile (3al)[4]

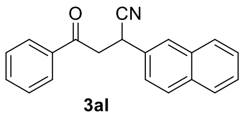

3al

The title compound was prepared according to the general procedure **A** as described. Silica gel flash column chromatography was performed using hexanes and ethyl acetate (10:1) (R*f* = 0.50 in hexane:ethyl acetate = 5:1) resulting in a white solid in 58% yield.

^{1}H NMR (600 MHz, $CDCl_3$) δ 7.89-7.82 (m, 3H), 7.82-7.73 (m, 3H), 7.51 (t, *J* = 7.4 Hz, 1H), 7.47–7.40 (m, 3H), 7.38 (t, *J* = 7.8 Hz, 2H), 4.66 (dd, *J* = 7.6 Hz, 6.3 Hz, 1H), 3.72 (dd, *J* = 17.9 Hz, 7.9 Hz, 1H), 3.52 (dd, *J* = 17.9 Hz, 6.0 Hz, 1H).

^{13}C NMR (151 MHz, $CDCl_3$) δ 194.63, 135.74, 133.91, 133.35, 132.9, 132.55, 129.32, 128.85, 128.13, 127.94, 127.75, 126.84, 126.76, 126.69, 124.83, 120.64, 44.53, 32.09.

2-(naphthalen-1-yl)-4-oxo-4-phenylbutanenitrile (3am)

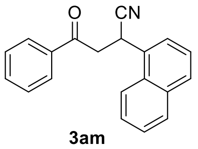

3am

The title compound was prepared according to the general procedure **A** as described. Silica gel flash column chromatography was performed using hexanes and ethyl acetate (10:1) (R*f* = 0.50 in hexane:ethyl acetate = 5:1) resulting in a white solid in 89% yield.

^{1}H NMR (600 MHz, $CDCl_3$) δ 7.88–7.81 (m, 4H), 7.78 (d, *J* = 8.3 Hz, 1H), 7.71 (d, *J* = 7.1 Hz, 1H), 7.53–7.47 (m, 2H), 7.44 (m, 2H), 7.37 (t, *J* = 7.8 Hz, 2H), 5.24 (dd, *J* =

9.7 Hz, 4.0 Hz, 1H), 3.79 (dd, *J* = 18.1 Hz, 9.7 Hz, 1H), 3.48 (dd, *J* = 18.1 Hz, 4.0 Hz, 1H).

^{13}C NMR (151 MHz, $CDCl_3$) δ 194.91, 135.69, 134.17, 133.93, 130.81, 129.76, 129.47, 129.34, 128.85, 128.17, 127.29, 126.33, 125.93, 125.54, 122.02, 120.68, 43.74, 29.06.

HRMS (ESI): m/z Calcd. for $[C_{20}H_{15}NO, M+H]^+$: 308.1046; Found: 308.1048.

2-(furan-2-yl)-4-oxo-4-phenylbutanenitrile (3an)[2]

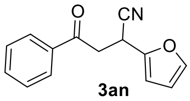

3an

The title compound was prepared according to the general procedure **A** as described. Silica gel flash column chromatography was performed using hexanes and ethyl acetate (10:1) (R*f* = 0.30 in hexane:ethyl acetate = 5:1) resulting in a white solid in 77% yield.

^{1}H NMR (600 MHz, $CDCl_3$) δ 7.88 (dd, *J* = 8.3, 1.1 Hz, 2H), 7.54 (t, *J* = 7.4 Hz, 1H), 7.41 (t, *J* = 7.8 Hz, 2H), 7.31 (d, *J* = 1.1 Hz, 1H), 6.31 (d, *J* = 3.3 Hz, 1H), 6.28 (dd, *J* = 3.2, 1.9 Hz, 1H), 4.60 (t, *J* = 6.9 Hz, 1H), 3.65-3.56 (m, 2H).

^{13}C NMR (151 MHz, $CDCl_3$) δ 194.28, 146.94, 143.14, 135.61, 133.99, 128.88, 128.14, 118.36, 110.86, 108.24, 40.71, 26.02.

4-oxo-4-phenyl-2-(thiophen-2-yl)butanenitrile (3ao)[2]

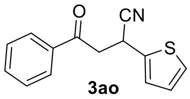

3ao

The title compound was prepared according to the general procedure **A** as described. Silica gel flash column chromatography was performed using hexanes and ethyl acetate (10:1) (R*f* = 0.30 in hexane:ethyl acetate = 5:1) resulting in a white solid in 67% yield (**method A**), 69% yield (**method B**).

^{1}H NMR (600 MHz, $CDCl_3$) δ 7.90–7.86 (m, 2H), 7.54 (t, *J* = 7.4 Hz, 1H), 7.42 (t, *J* = 7.8 Hz, 2H), 7.22–7.17 (m, 1H), 7.09 (d, *J* = 3.5 Hz, 1H), 6.91 (dd, *J* = 5.1 Hz, 3.6 Hz, 1H), 4.79 (t, *J* = 6.8 Hz, 1H), 3.69 (dd, *J* = 17.8 Hz, 7.3 Hz, 1H), 3.56 (dd, *J* = 17.8 Hz, 6.5 Hz, 1H).

^{13}C NMR (151 MHz, $CDCl_3$) δ 194.26, 137.04, 135.63, 134.02, 128.89, 128.15, 127.18, 126.73, 125.90, 44.59, 27.27.

2-(1H-indol-3-yl)-4-oxo-4-phenylbutanenitrile (3ap)

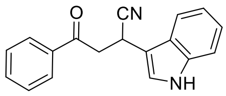

3ap

The title compound was prepared according to the general procedure **A** as described. Silica gel flash column chromatography was performed using hexanes and ethyl acetate (10:1) (R*f* = 0.30 in hexane:ethyl acetate = 5:1) resulting in a white solid in 51% yield.

^{1}H NMR (600 MHz, $CDCl_3$) δ 8.21 (s, 1H), 7.85 (d, *J* = 7.5 Hz, 2H), 7.62 (d, *J* = 7.9 Hz, 1H), 7.50 (t, *J* = 7.4 Hz, 1H), 7.38 (t, *J* = 7.7 Hz, 2H), 7.33 (d, *J* = 8.1 Hz, 1H), 7.22 (d, *J* = 2.0 Hz, 1H), 7.18 (t, *J* = 7.4 Hz, 1H), 7.11 (t, *J* = 7.5 Hz, 1H), 4.77 (dd, *J* = 7.9, 5.9 Hz, 1H), 3.70 (dd, *J* = 17.9, 8.1 Hz, 1H), 3.60 (dd, *J* = 17.9, 5.7 Hz, 1H).

^{13}C NMR (151 MHz, $CDCl_3$) δ 195.31, 136.50, 135.88, 133.82, 128.81, 128.11, 125.00, 122.93, 122.89, 120.79, 120.39, 118.36, 111.77, 109.76, 42.94, 23.73.

HRMS (ESI): m/z Calcd. for $[C_{18}H_{14}N_2O, M+H]^+$: 297.0998; Found: 297.1003.

2-benzyl-4-oxo-4-phenylbutanenitrile (3aq)

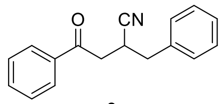

3aq

The title compound was prepared according to the general procedure **A** as described. Silica gel flash column chromatography was performed using hexanes and ethyl acetate (10:1) (R*f* = 0.50 in hexane:ethyl acetate = 5:1) resulting in a white solid in 64% yield.

^{1}H NMR (600 MHz, $CDCl_3$) δ 7.84 (dd, *J* = 8.3 Hz, 1.1 Hz, 2H), 7.53 (dd, *J* = 10.6 Hz, 4.3 Hz, 1H), 7.41 (t, *J* = 7.8 Hz, 2H), 7.29-7.24 (m, 2H), 7.21 (dd, *J* = 7.1 Hz, 5.0 Hz, 3H), 3.47 (dq, *J* = 13.2 Hz, 6.6 Hz, 1H), 3.28 (dd, *J* = 17.9 Hz, 6.5 Hz, 1H), 3.17 (dd, *J* = 17.9 Hz, 6.9 Hz, 1H), 2.94 (qd, *J* = 13.7 Hz, 7.0 Hz, 2H).

^{13}C NMR (151 MHz, $CDCl_3$) δ 195.25, 136.41, 135.93, 133.86, 129.20, 128.86, 128.04, 127.49, 121.42, 39.79, 37.55, 28.21.

HRMS (ESI): m/z Calcd. for $[C_{17}H_{15}NO, M+H]^+$: 272.1046; Found: 272.1043.

2-cyclohexyl-4-oxo-4-phenylbutanenitrile (3ar)

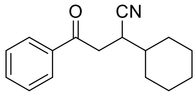

3ar

The title compound was prepared according to the general procedure as described. Silica gel flash column chromatography was performed using hexanes and ethyl acetate (10:1) (R*f* = 0.50 in hexane:ethyl acetate = 5:1) resulting in a white solid in 83% yield (**method A**), 82% yield (**method B**).

^{1}H NMR (600 MHz, $CDCl_3$) δ 7.88 (d, J = 7.4 Hz, 2H), 7.53 (t, J = 7.4 Hz, 1H), 7.41 (t, J = 7.7 Hz, 2H), 3.31 (td, J = 9.3 Hz, 3.3 Hz, 1H), 3.17 (dt, J = 20.0 Hz, 5.7 Hz, 2H), 1.82 (d, J = 6.3 Hz, 1H), 1.72 (d, J = 10.5 Hz, 3H), 1.62 (d, J = 11.2 Hz, 1H), 1.52 (s, 1H), 1.25-1.06 (m, 5H).

^{13}C NMR (151 MHz, $CDCl_3$) δ 195.55, 136.07, 133.74, 128.82, 128.05, 120.97, 38.97, 38.45, 32.55, 31.46, 29.20, 25.99, 25.86, 25.81.

2-(2-(methylthio)ethyl)-4-oxo-4-phenylbutanenitrile (3as)

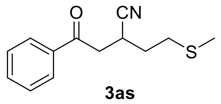

3as

The title compound was prepared according to the general procedure **A** as described. Silica gel flash column chromatography was performed using hexanes and ethyl acetate (10:1) (R*f* = 0.40 in hexane:ethyl acetate = 5:1) resulting in a white solid in 32% yield.

^{1}H NMR (600 MHz, $CDCl_3$) δ 7.88 (d, J = 7.3 Hz, 2H), 7.55 (t, J = 7.4 Hz, 1H), 7.43 (t, J = 7.8 Hz, 2H), 3.50-3.42 (m, 1H), 3.38 (dd, J = 17.8 Hz, 6.4 Hz, 1H), 3.23 (dd, J = 17.8 Hz, 6.9 Hz, 1H), 2.71 (dt, J = 13.2 Hz, 6.6 Hz, 1H), 2.61 (dt, J = 7.9 Hz, 6.3 Hz, 1H), 2.08 (s, 3H), 1.95-1.87 (m, 2H).

^{13}C NMR (151 MHz, $CDCl_3$) δ 194.93, 135.87, 133.90, 128.88, 128.04, 121.23, 40.50, 31.43, 31.25, 25.43, 15.44.

HRMS (ESI): m/z Calcd. for $[C_{13}H_{15}NOS, M+H]^+$: 256.0767; Found: 256.0769.

3-methyl-4-oxo-2,4-diphenylbutanenitrile (8)

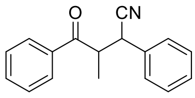

8

The title compound was prepared according to the general procedure **A** as described. Silica gel flash column chromatography was performed using hexanes and ethyl acetate (10:1) (Rf = 0.30 in hexane:ethyl acetate = 5:1) resulting in a white solid in 31% yield (**method A**), 25% yield (**method B**).

^{1}H NMR (600 MHz, $CDCl_3$) δ 7.99-7.84 (m, 2H), 7.50 (t, J = 7.4 Hz, 1H), 7.40 (t, J = 7.8 Hz, 2H), 7.35 (d, J = 7.2 Hz, 2H), 7.29 (t, J = 7.6 Hz, 2H), 7.22 (t, J = 7.3 Hz, 1H), 4.93 (d, J = 8.0 Hz, 1H), 3.83-3.65 (m, 1H), 1.00 (d, J = 7.2 Hz, 3H).

2-((3*S*, 8*R*, 9*S*, 10*R*, 13*S*, 14*S*)-3-hydroxy-10,13-dimethyl-17-oxo-2, 3, 4, 7, 8, 9, 10, 11, 12, 13, 14, 15, 16, 17-tetradecahydro-1H-cyclopenta[a]phenanthren-16-yl)-2-phenylacetonitrile (10)

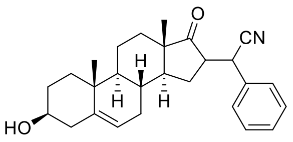

The title compound was prepared according to the general procedure **B** as described. Silica gel flash column chromatography was performed using hexanes and ethyl acetate (6:1) (R*f* = 0.40 in hexane:ethyl acetate = 3:1) resulting in a white solid in 57% yield.

^{1}H NMR (600 MHz, $CDCl_3$) δ 7.50-7.43 (m, 2H), 7.36 (dt, *J* = 15.1 Hz, 5.0 Hz, 2H), 7.30 (d, *J* = 7.4 Hz, 1H), 5.35-5.28 (m, 1H), 3.51-3.43 (m, 1H), 2.83 (ddd, *J* = 15.8 Hz, 6.6 Hz, 1.9 Hz, 1H), 2.45-2.32 (m, 1H), 2.26 (dd, *J* = 5.2 Hz, 2.2 Hz, 1H), 2.20 (d, *J* = 2.7 Hz, 1H), 2.16-2.09 (m, 1H), 2.07-1.96 (m, 1H), 1.92 (ddd, *J* = 12.9 Hz, 4.3 Hz, 2.5 Hz, 1H), 1.84-1.69 (m, 3H), 1.69-1.58 (m, 3H), 1.54-1.40 (m, 3H), 1.37-1.25 (m, 3H), 1.00 (s, 3H), 0.92 (s, 3H).

^{13}C NMR (151 MHz, $CDCl_3$) δ 209.61, 141.19, 135.99, 135.65, 133.08, 130.32, 128.68, 120.83, 71.62, 50.38, 49.92, 47.34, 42.25, 37.16, 36.75, 31.62, 31.60, 31.23, 30.95, 29.37, 20.44, 19.47, 14.23.

IV Procedure of gram scale reaction

To a vial equipped with a dried stir bar was added *o*-Anisaldehyde (5 mmol) acetophenone cyanohydrin (10 mmol) $NiCl_2$ (5 mol%), ligand **L** (5 mol%), LiOH (300 mol%), 300 mg 4Å MS and anhydrous dioxane (10 mL) in the glovebox. The reaction mixture was taken outside the glovebox and allowed to stir at room temperature for 30 min. After then, the reaction mixture was allowed to stir at 100 °C for 18 hours. The crude reaction mixture was concentrated under reduced pressure and directly purified by silica gel chromatography to give pure product 1.12 g, 85% yield.

V The mechanism study.

To a vial equipped with a dried stir bar was added Chalcone **11** (0.2 mmol), *p*-Tolualdehyde (0.2 mmol), acetophenone cyanohydrin (0.4 mmol), $NiCl_2$ (5 mol%), ligand L (5 mol%), LiOH (0.6 mmol), 100 mg 4Å MS and anhydrous dioxane (1 mL) in the glovebox. The reaction mixture was taken outside the glovebox and allowed to stir at room temperature for 30 min. After then, the reaction mixture was allowed to stir at 100 oC for 18 hours. The crude reaction mixture was concentrated under reduced pressure and given ^{1}H NMR. We got the corresponding products **3aa** and **3ae** with the ratio of 1.05:1, which shows that the cyano group from the cleavage of C-CN bond of cyanohydrin was a free anion in this nickel-catalyzed protocol and has the same opportunity to conjugated addition to each chalcone.

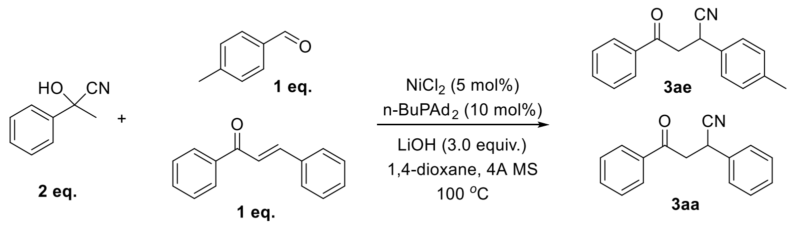

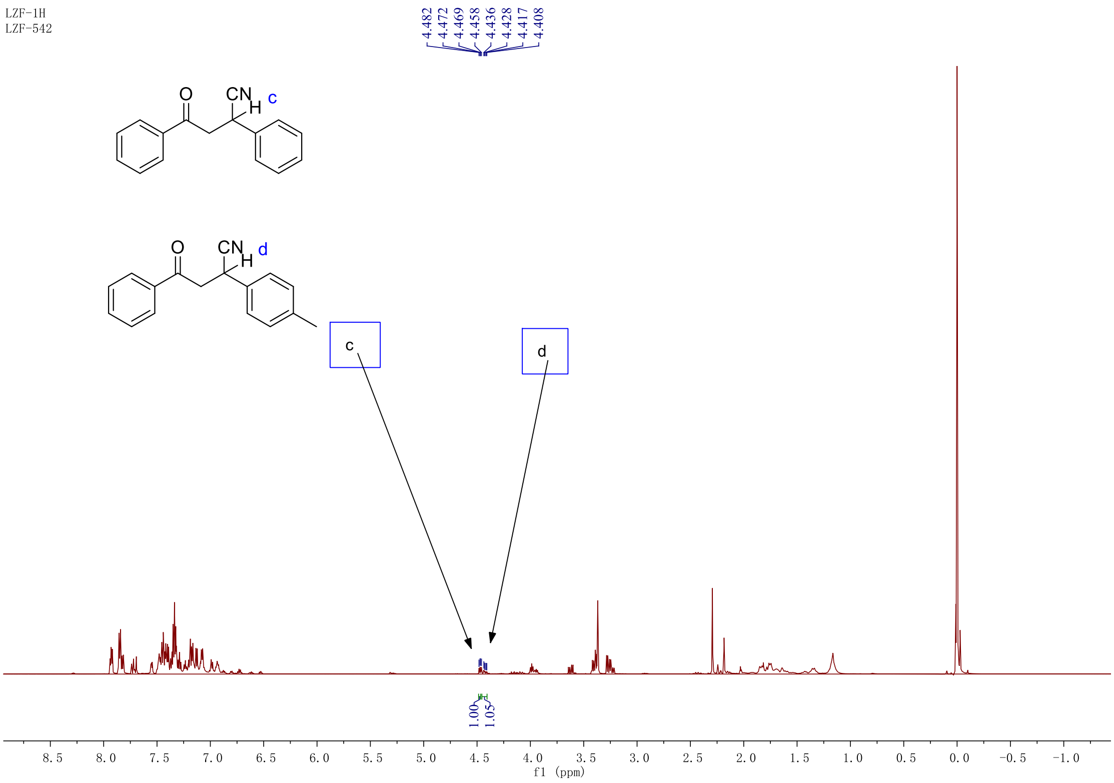

To a vial equipped with a dried stir bar was added Chalcone **11** (0.2 mmol), acetophenone cyanohydrin (0.4 mmol), under standard conditions in the glovebox. The reaction mixture was taken outside the glovebox and allowed to stir at room temperature for 30 min. After then, the reaction mixture was allowed to stir at 100 ^{o}C for 18 hours. The crude reaction mixture was concentrated under reduced pressure and given ^{1}H NMR. We got the corresponding products **3aa** with 89% yield, and the hydrogen-borrowing product **12** was not observed, showing that cleavage the C-CN bond is more easy than the C-H bond in cyanohydrins.

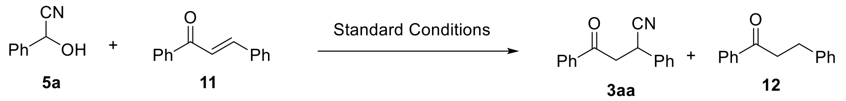

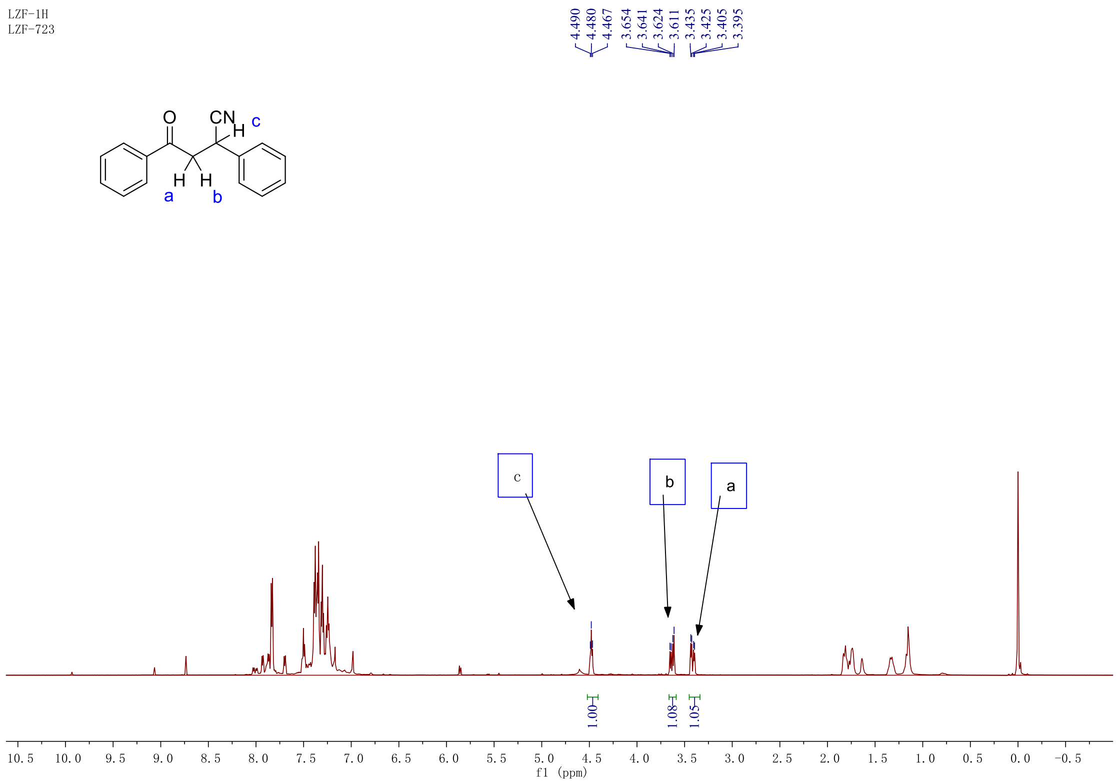

In order to observe the role of nickel in this cyano borrowing reaction, the ^{31}P NMR and HIMS were tested. As it shown below, the peak of the mixture of $NiCL_2$ and ^{n}BuPAd$_2$ in ^{31}P NMR spectrum is 24.04 ppm, but after the addition of acetophenone cyanohydrin **1a**, the 31P was remove to 51.34 ppm and 68.76 ppm, which means that the nickel was interacted with the cyanohydrins. Additionally, HIMS was tested under the standard procedure A, we found the fragment of [(^{n}BuPAd$_2$)$_2$Ni(CN)(**1a**)]. All these results could

prove that the coordination of CN toward Ni.

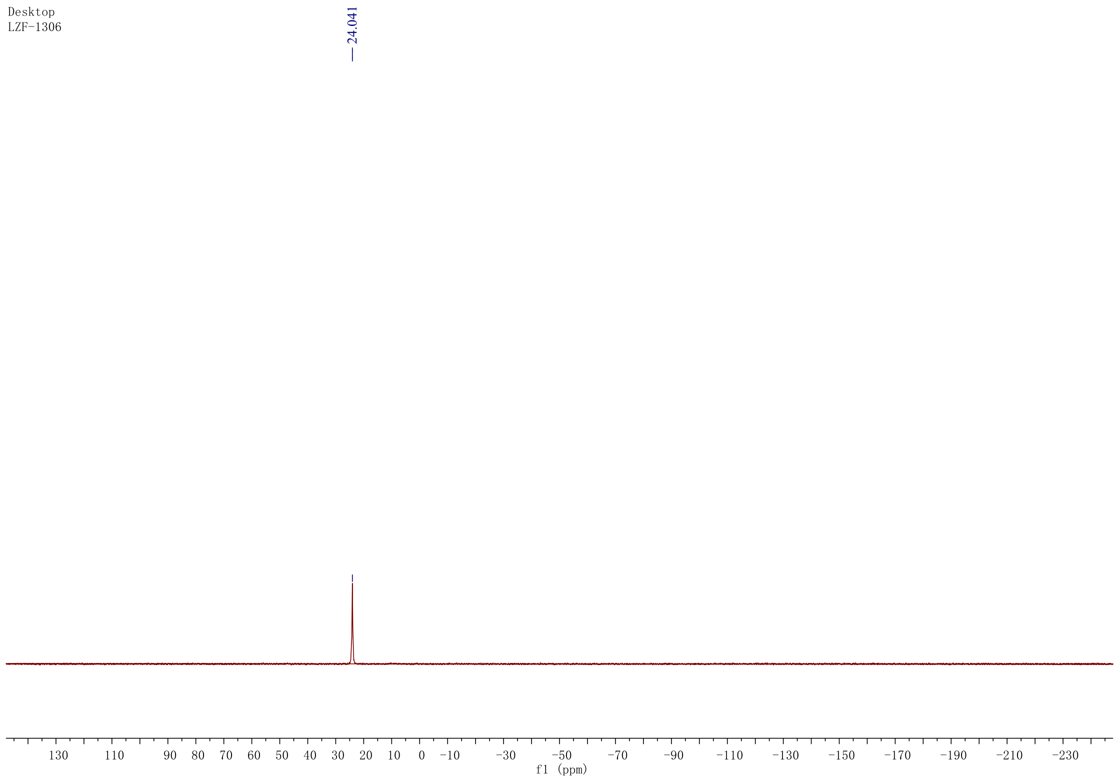

The ^{31}P NMR of the mixture of NiCL$_2$ and ^{n}BuPAd$_2$.

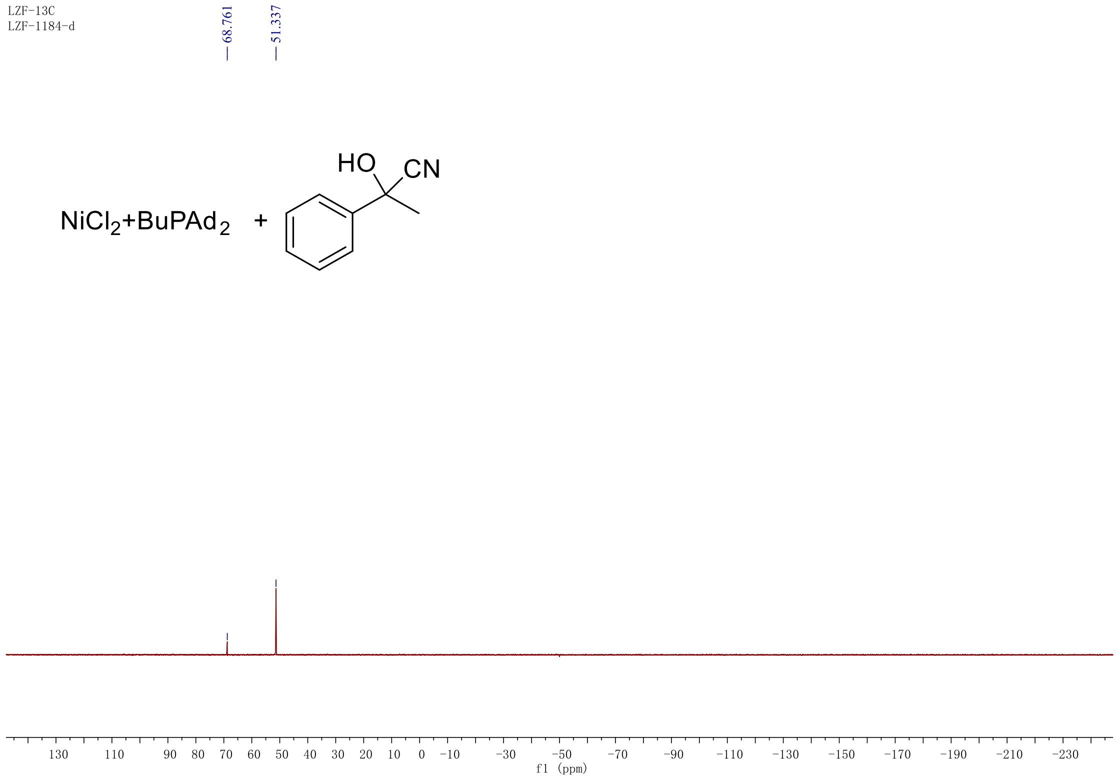

The ^{31}P NMR of the mixture of NiCL$_2$, ^{n}BuPAd$_2$ and acetophenone cyanohydrin 1a.

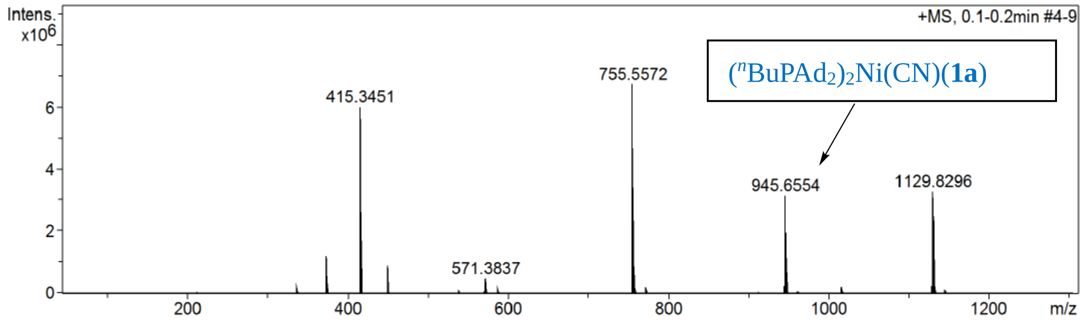

The HIMS of the reaction mixture under standard condition.

In order to understand the role of nickel catalyst in mechanism, we have tested every step of the reaction in the presence and absence of the nickel catalyst in 1 h and 18 h, respectively, as it shows in figure 1. We found that the nickel catalyst improves the conjugate 1,4-addition of the cyano group to chalcone.

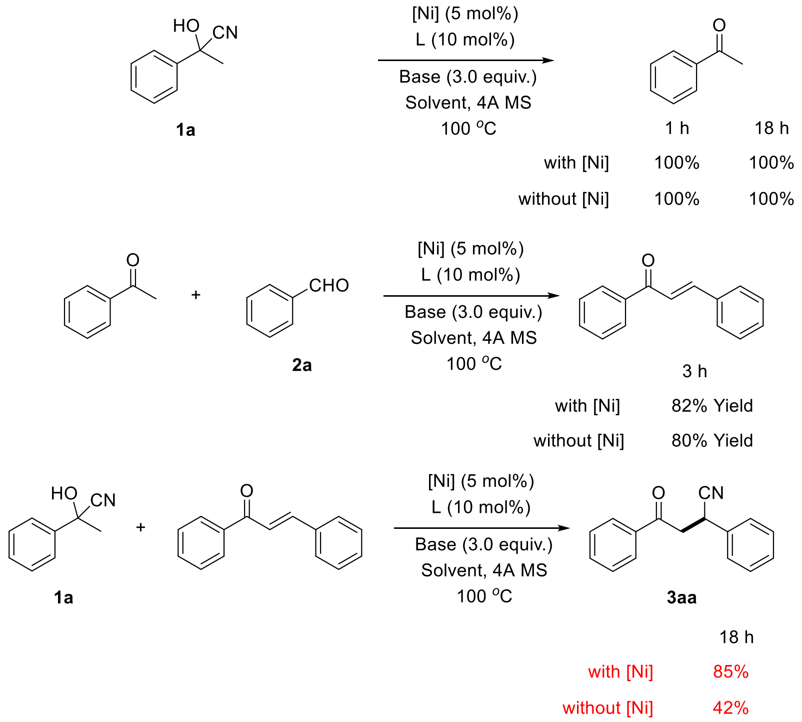

The control experiments with other Lewis acid catalyst, such as Ti(O^{i}-Pr)$_4$ was tested, and got 35% Yield. The Brϕnsted acid catalyst, such as benzoic acid ($PhCO_2H$) was used in this cyano-borrowing reaction, 23% Yield was obtained. These control experiments shown that the acid could improve the reaction, but nickel catalyst was the optimal choose. All these control experiments shows that the Lewis acid was the role playing for nickel complex, and the oxidation number of Ni does not change through the cyano borrowing process.

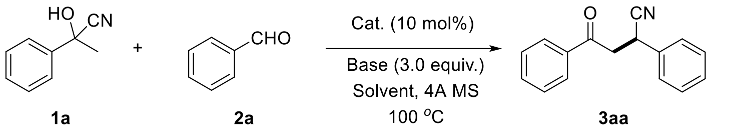

Cat. = $Ti(O^{i}Pr)_4$ (Lewis acid): 35% Yield
Cat. = $PhCO_2H$ (Bronsted acid): 23% Yield

LiOH is very important in this nickel-catalyzed cyano borrowing process, and the reaction with different equivalents of LiOH were tested, the results were listed as below. The transformation did not work while catalytic amounts was tested, and trace desired product was obtained with one equivalent LiOH. But the desired product 3 was obtained in 64% yield while two equivalent of LiOH was introduced to the cyano borrowing reaction, and 85% yield was achieved under the standard reaction conditions.

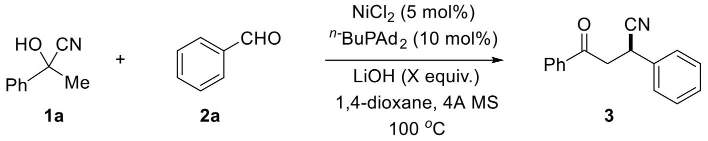

Entry	LiOH (eq.)	Yield (%)
1	0.2	NR
2	0.5	NR
3	1.0	Trace
4	2.0	64
5	3.0	85

In addition, LiOH was very important in this transformation, not just work as the base for the Aldol condensation, but also important in the “Borrowing” and “Returning”. As it shows below, the nickel catalyzed cyano borrowing reaction has no activity at the absence of LiOH. Low conversion was achieved for the decyanoation of the cyanohydrins and no Micheal addition was occurred without the addition of LiOH. Overall, lithium hydroxide is the key additive for the transformation.

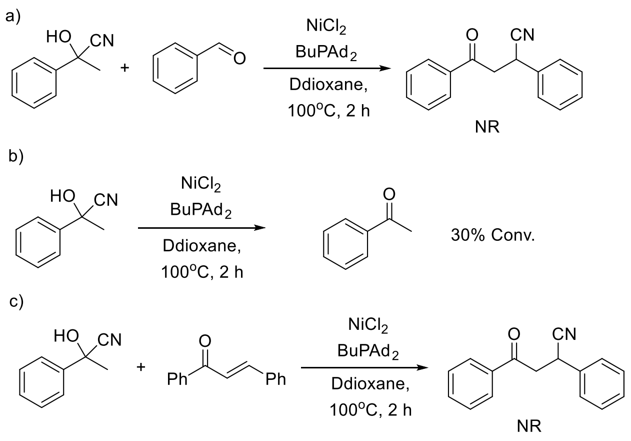

VI References

1, Huang, X.; Wang, W. *Synlett* **2017**, *28*, 439-444.

2, Lin, S.; Wei, Y.; Liang, F. *Chem. Commun.*, **2012**, *48*, 9879-9881.

3, Wang, Y.; Chen, X. F. *Eur. J. Org. Chem***. 2013**, 4624–463.

4, Zhang, J.; Liu, X.; Wang, R. *Chem. Eur. J***. 2014**, *20*, 4911 – 4915.

VII NMR of products

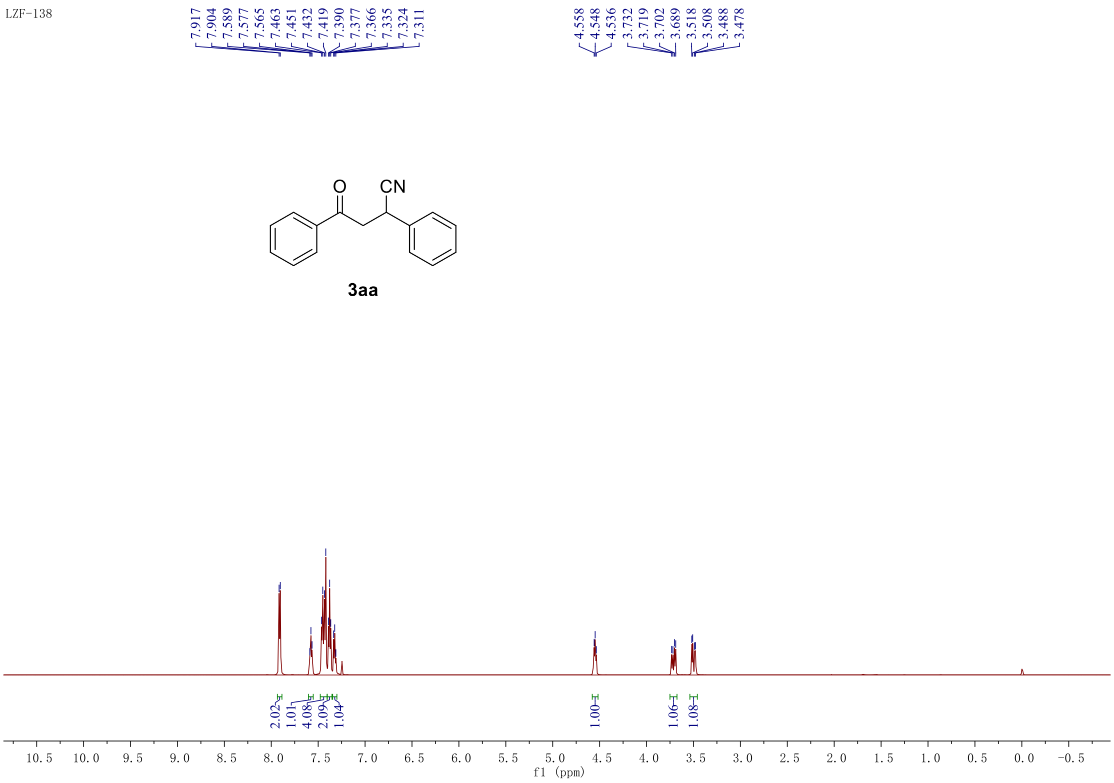

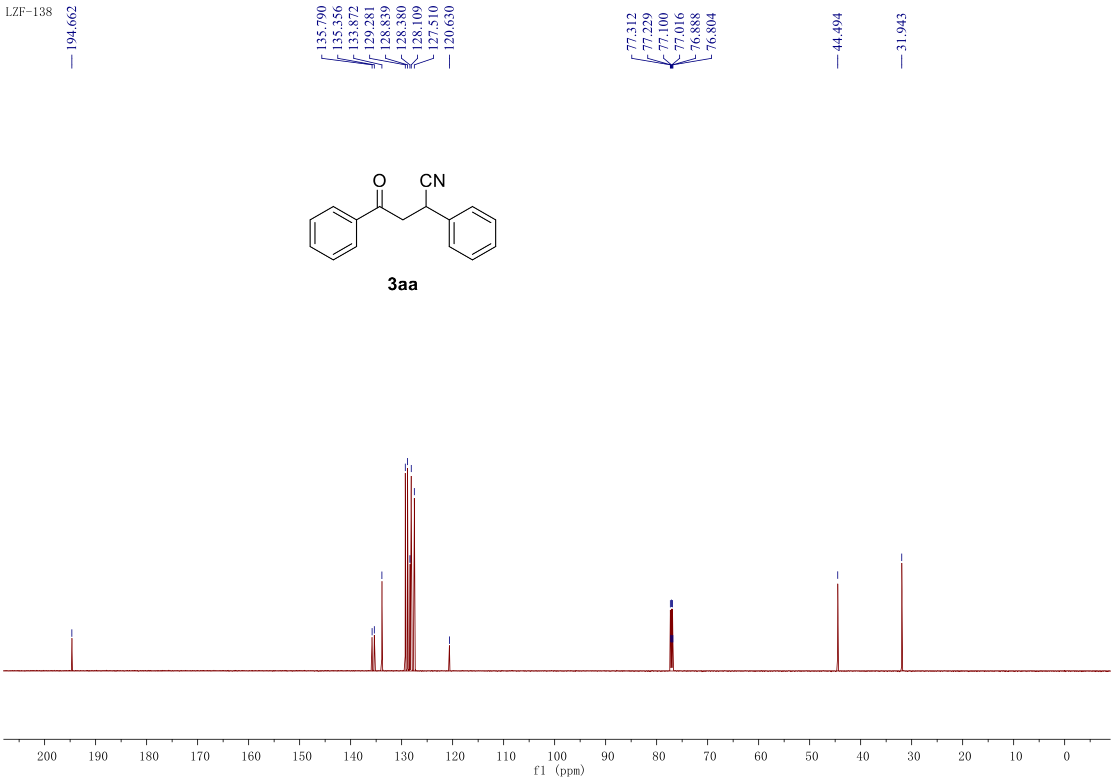

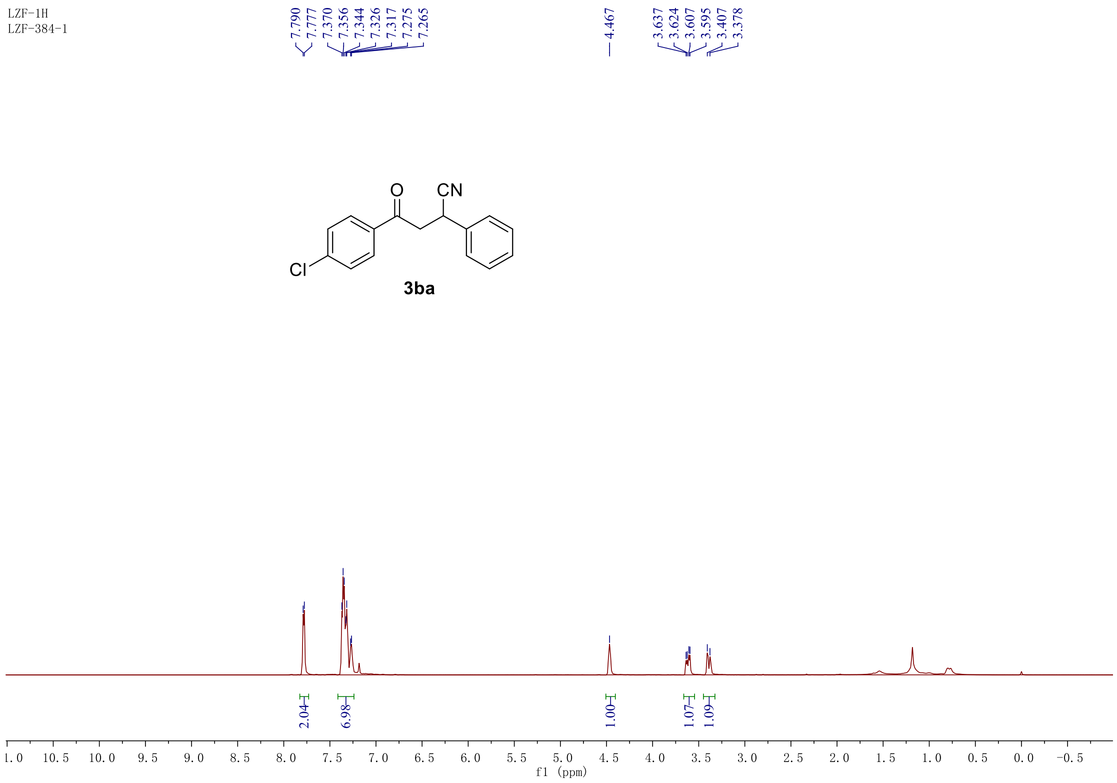
LZF-1H
LZF-384-1
7.790
7.777
7.370
7.356
7.344
7.326
7.317
7.275
7.265
4.467
3.637
3.624
3.607
3.595
3.407
3.378
O
CN
Cl
3ba
2.04
6.98
1.00
1.07
1.09
f1 (ppm)

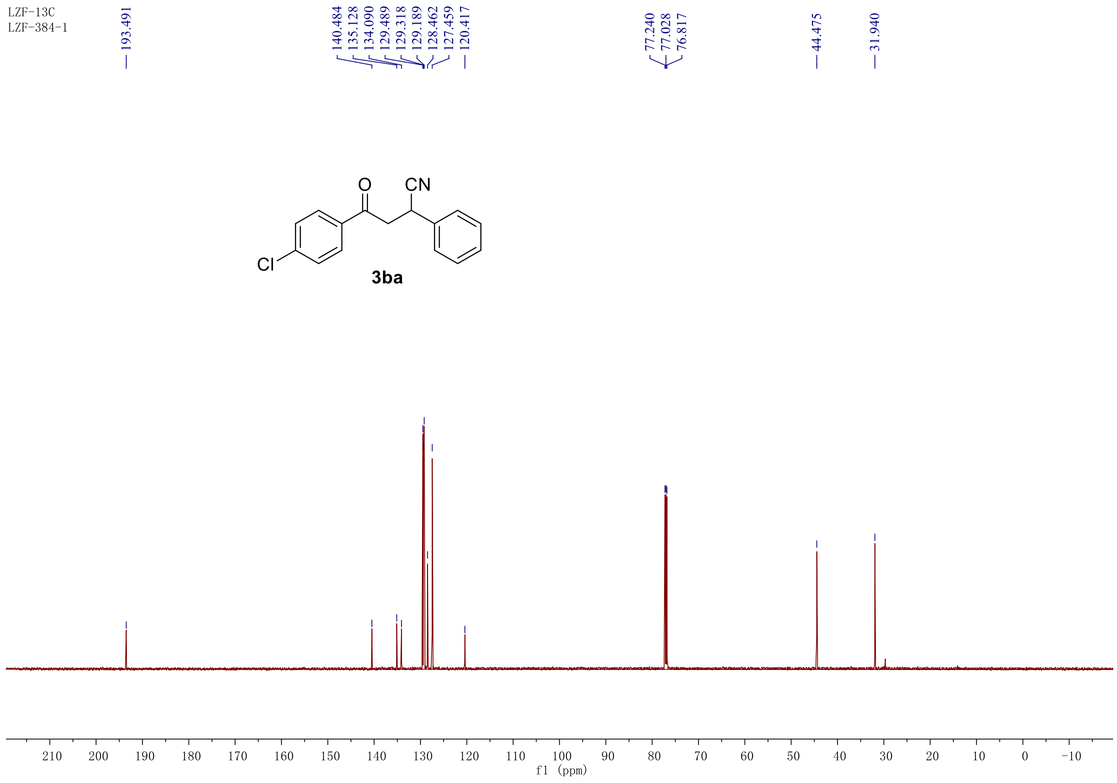
LZF-13C
LZF-384-1
193.491
140.484
135.128
134.090
129.489
129.318
129.189
128.462
127.459
120.417
77.240
77.028
76.817
44.475
31.940
O
CN
Cl
3ba
f1 (ppm)

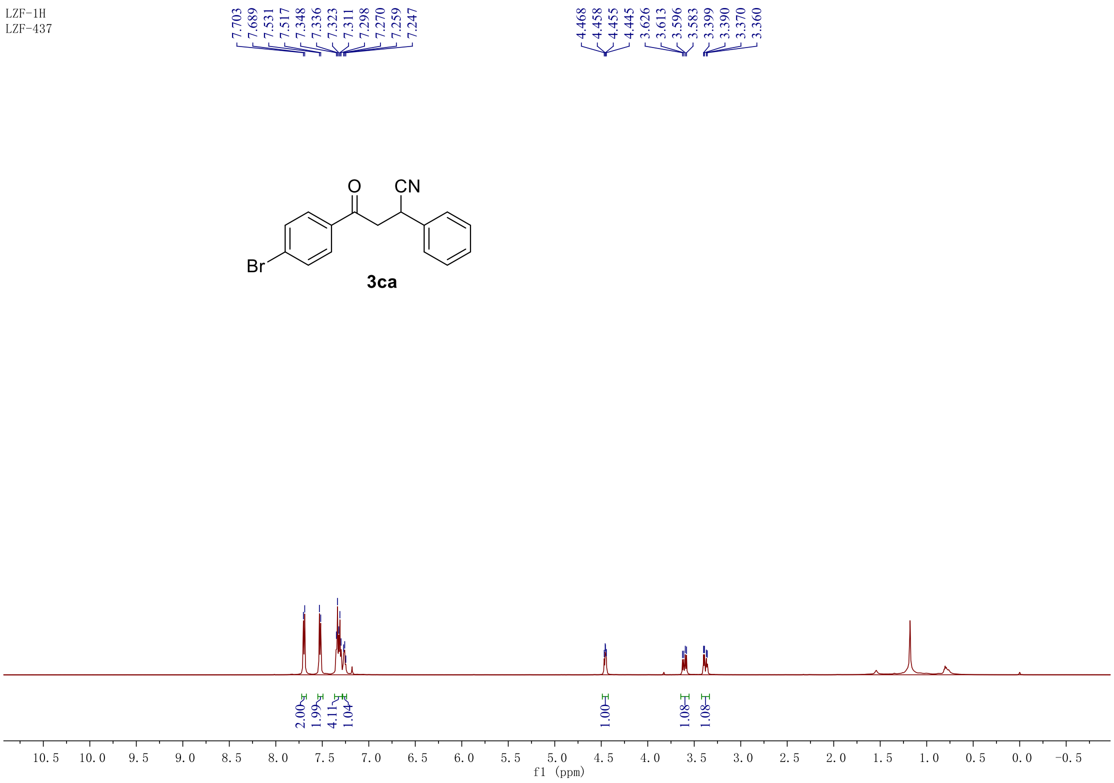
LZF-1H
LZF-437
7.703
7.689
7.531
7.517
7.348
7.336
7.323
7.311
7.298
7.270
7.259
7.247
4.468
4.458
4.455
4.445
3.626
3.613
3.596
3.583
3.399
3.390
3.370
3.360
O
CN
Br
3ca
2.00
1.99
4.11
1.04
1.00
1.08
1.08
f1 (ppm)

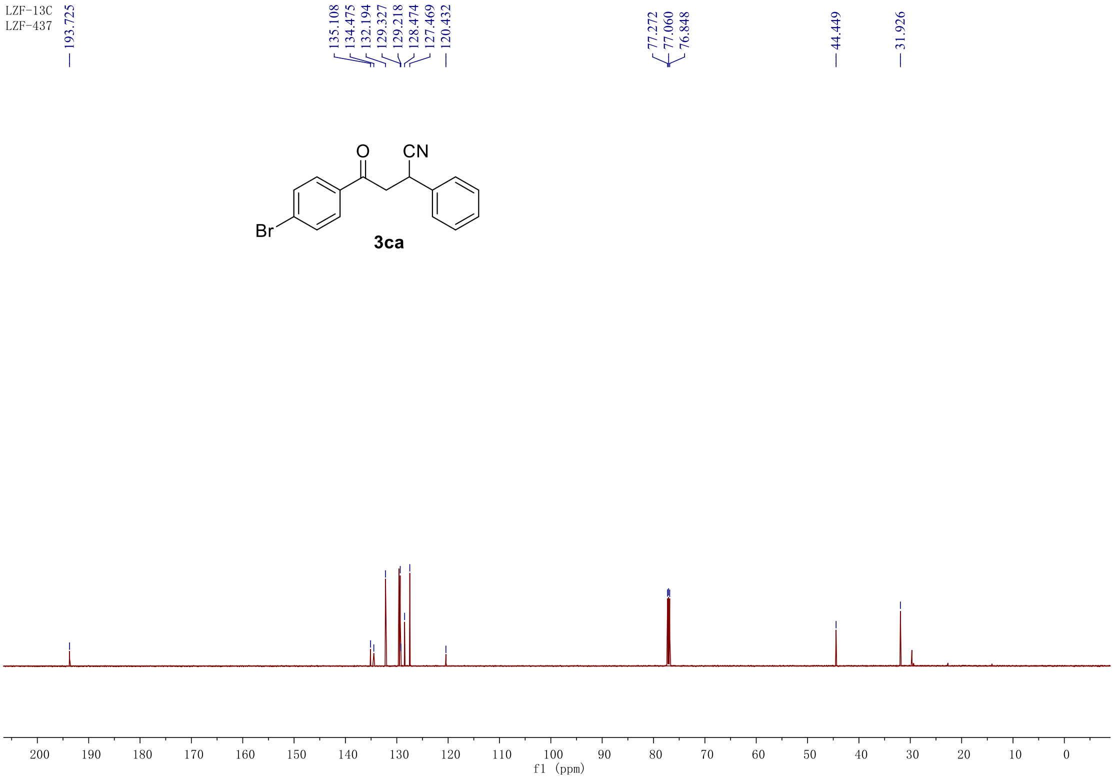
LZF-13C
LZF-437
193.725
135.108
134.475
132.194
129.327
129.218
128.474
127.469
120.432
77.272
77.060
76.848
44.449
31.926
O
CN
Br
3ca
f1 (ppm)

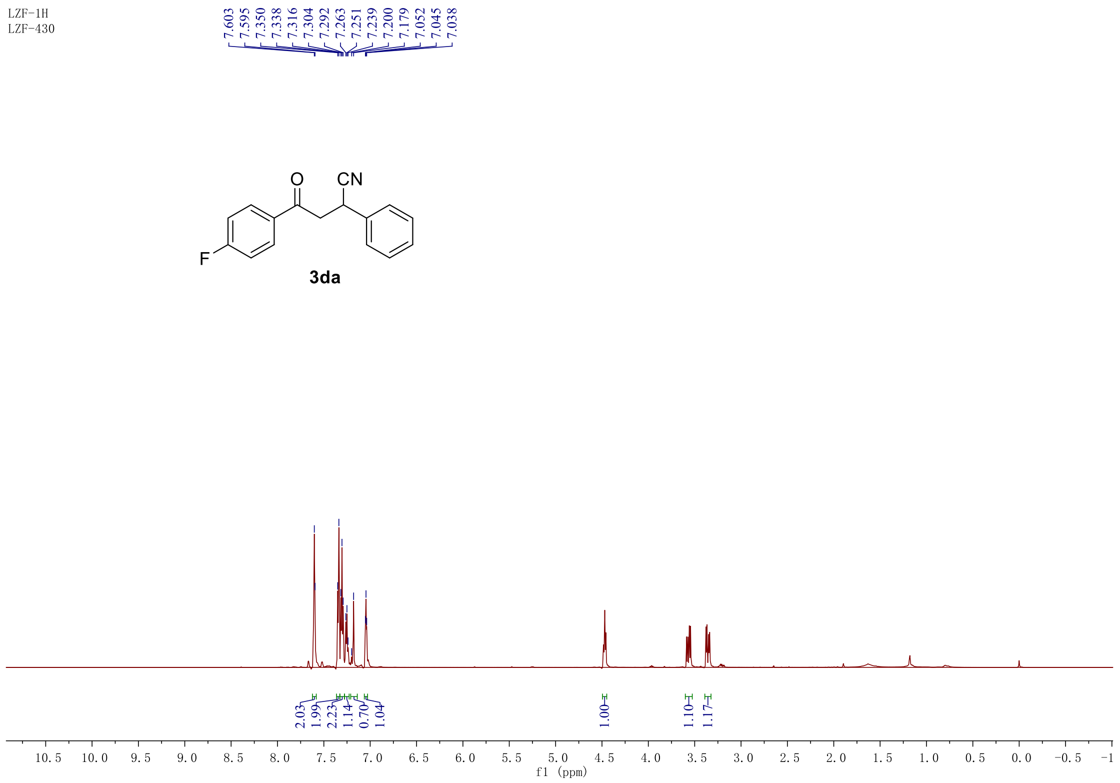
LZF-1H
LZF-430
7.603
7.595
7.350
7.338
7.316
7.304
7.292
7.263
7.251
7.239
7.200
7.179
7.052
7.045
7.038
O
CN
F
3da
2.03
1.99
2.23
1.14
0.70
1.04
1.00
1.10
1.17
f1 (ppm)

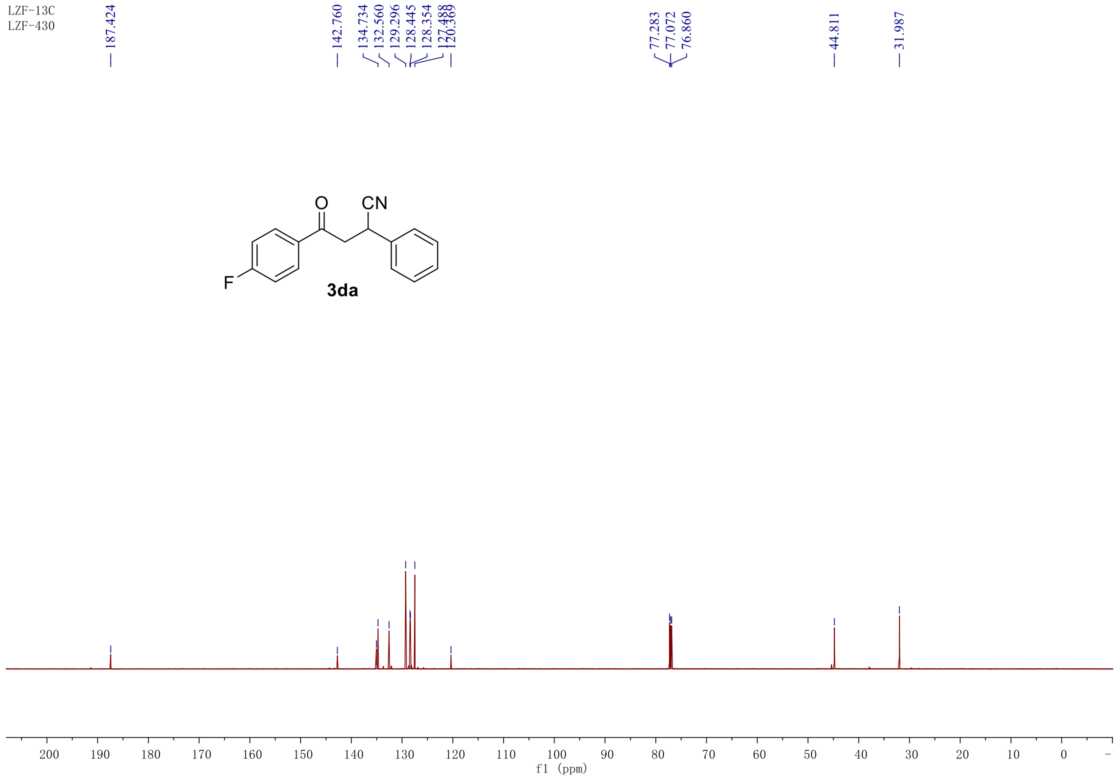
LZF-13C
LZF-430
187.424
142.760
134.734
132.560
129.296
128.445
128.354
127.488
120.369
77.283
77.072
76.860
44.811
31.987
O
CN
F
3da
f1 (ppm)

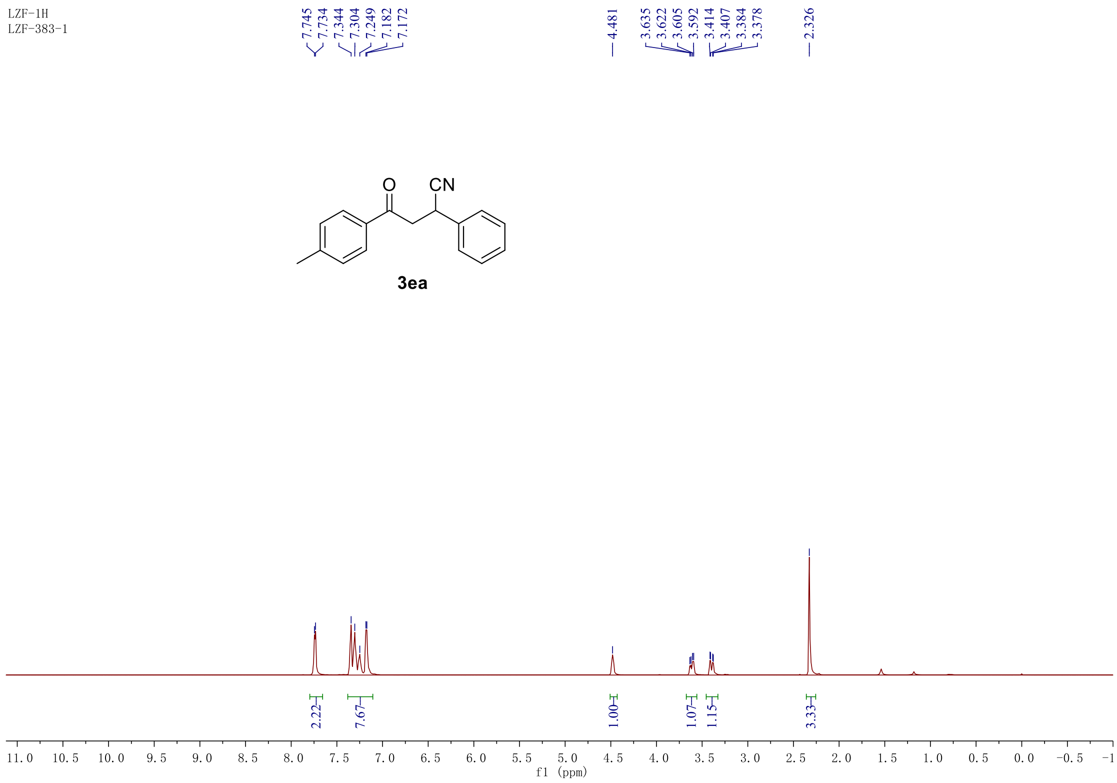
LZF-1H
LZF-383-1
7.745
7.734
7.344
7.304
7.249
7.182
7.172
4.481
3.635
3.622
3.605
3.592
3.414
3.407
3.384
3.378
2.326
O
CN
3ea
2.22
7.67
1.00
1.07
1.15
3.33
11.0 10.5 10.0 9.5 9.0 8.5 8.0 7.5 7.0 6.5 6.0 5.5 5.0 4.5 4.0 3.5 3.0 2.5 2.0 1.5 1.0 0.5 0.0 -0.5 -1
f1 (ppm)

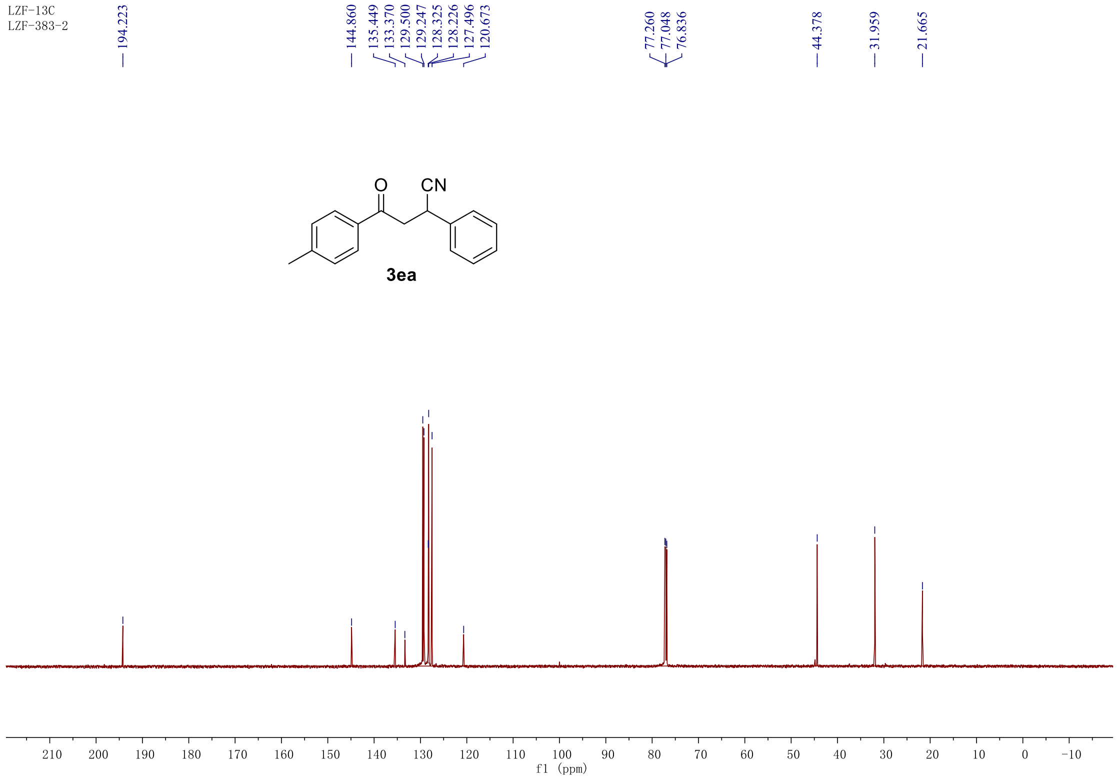
LZF-13C
LZF-383-2
194.223
144.860
135.449
133.370
129.500
129.247
128.325
128.226
127.496
120.673
77.260
77.048
76.836
44.378
31.959
21.665
O
CN
3ea
210 200 190 180 170 160 150 140 130 120 110 100 90 80 70 60 50 40 30 20 10 0 -10
f1 (ppm)

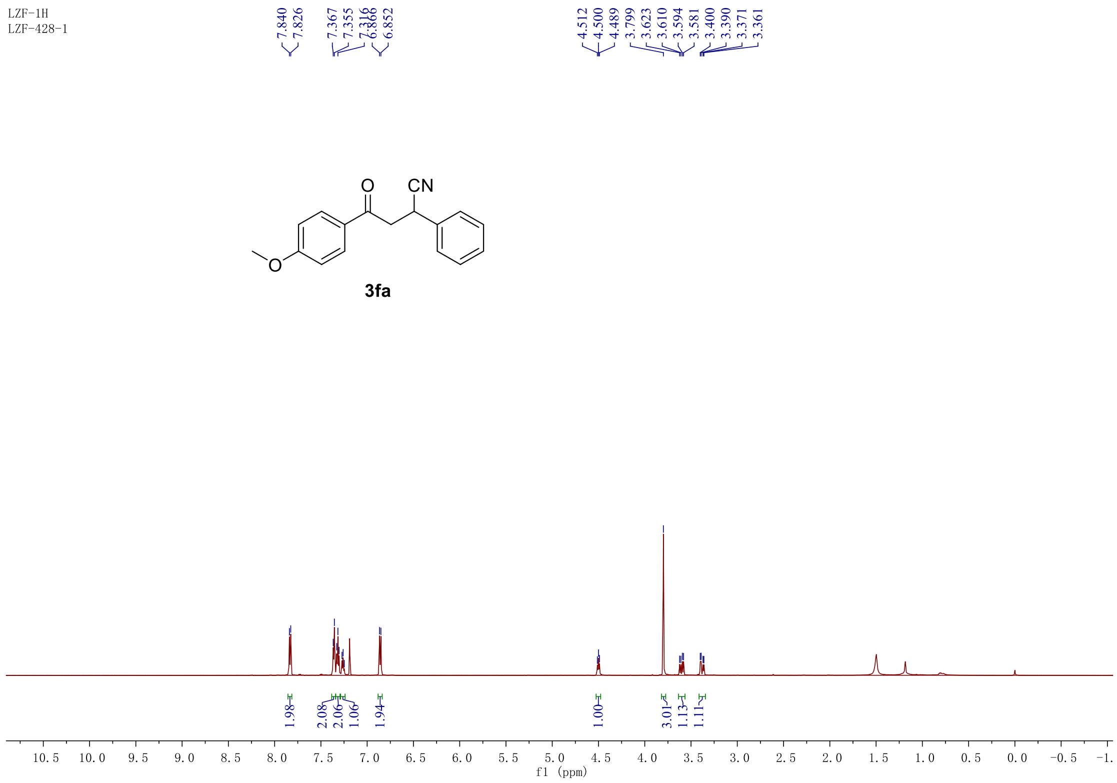
LZF-1H
LZF-428-1
7.840
7.826
7.367
7.355
7.316
6.866
6.852
4.512
4.500
4.489
3.799
3.623
3.610
3.594
3.581
3.400
3.390
3.371
3.361
O
CN
O
3fa
1.98
2.08
2.06
1.06
1.94
1.00
3.01
1.13
1.11
f1 (ppm)

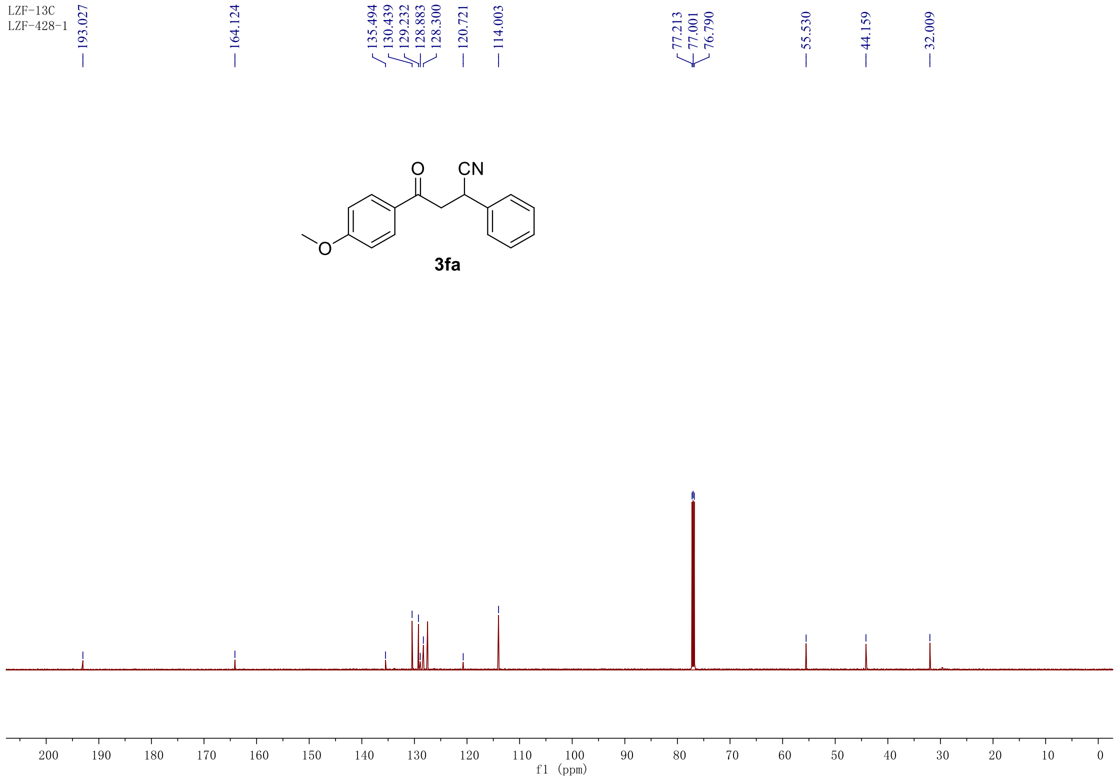
LZF-13C
LZF-428-1
193.027
164.124
135.494
130.439
129.232
128.883
128.300
120.721
114.003
77.213
77.001
76.790
55.530
44.159
32.009
O
CN
O
3fa
f1 (ppm)

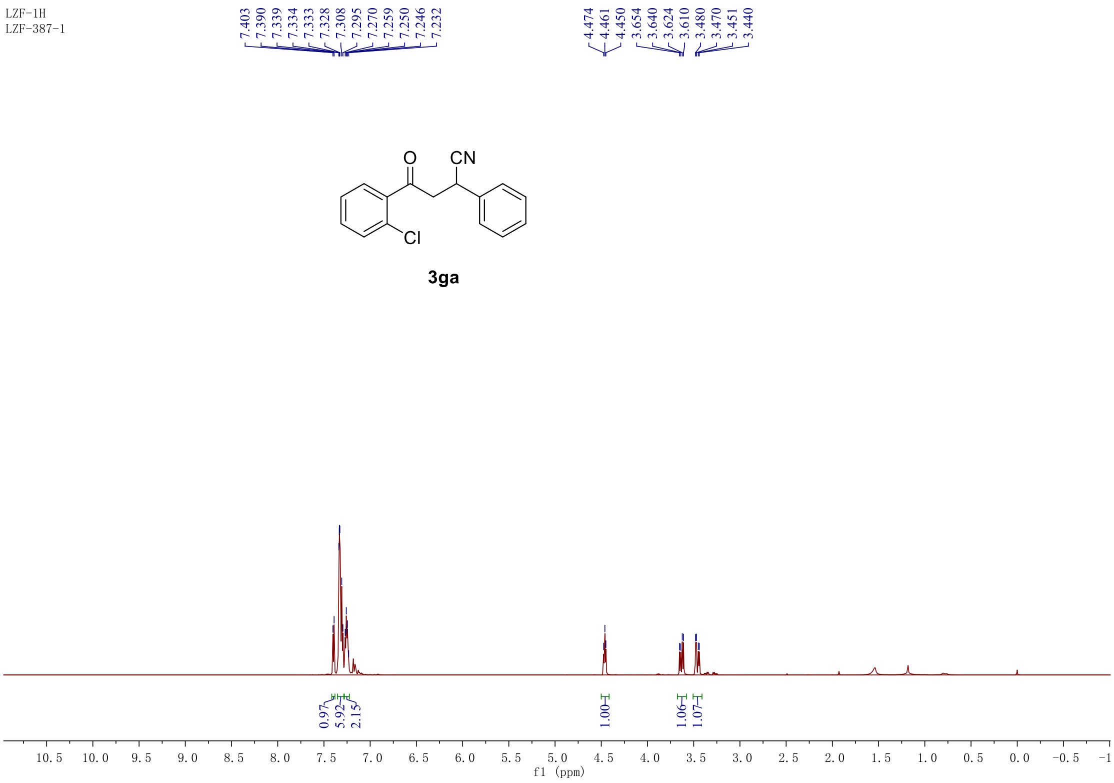
LZF-1H
LZF-387-1
7.403 7.390 7.339 7.334 7.333 7.328 7.308 7.295 7.270 7.259 7.250 7.246 7.232
4.474 4.461 4.450 3.654 3.640 3.624 3.610 3.480 3.470 3.451 3.440
O
CN
Cl
3ga
0.97 5.92 2.15
1.00
1.06 1.07
f1 (ppm)

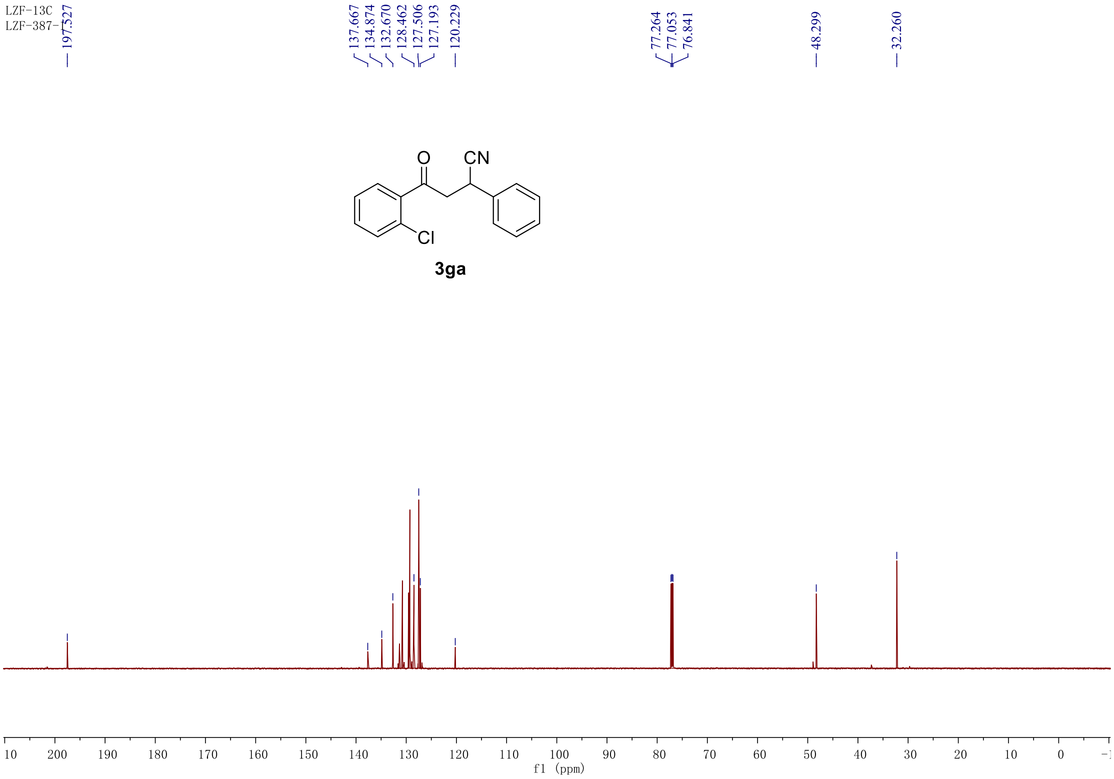
LZF-13C
LZF-387-1
197.527
137.667 134.874 132.670 128.462 127.506 127.193 120.229
77.264 77.053 76.841
48.299
32.260
O
CN
Cl
3ga
f1 (ppm)

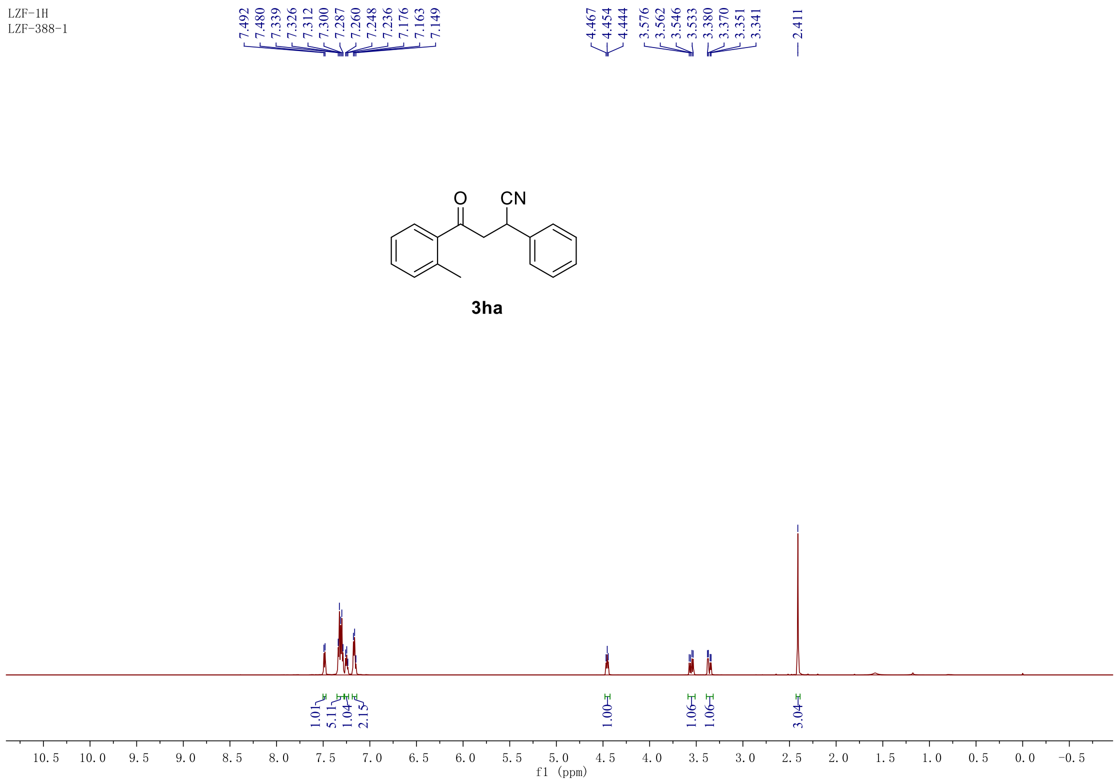
LZF-1H
LZF-388-1
7.492
7.480
7.339
7.326
7.312
7.300
7.287
7.260
7.248
7.236
7.176
7.163
7.149
4.467
4.454
4.444
3.576
3.562
3.546
3.533
3.380
3.370
3.351
3.341
2.411
O
CN
3ha
1.01
5.11
1.04
2.15
1.00
1.06
1.06
3.04
10.5 10.0 9.5 9.0 8.5 8.0 7.5 7.0 6.5 6.0 5.5 5.0 4.5 4.0 3.5 3.0 2.5 2.0 1.5 1.0 0.5 0.0 -0.5
f1 (ppm)

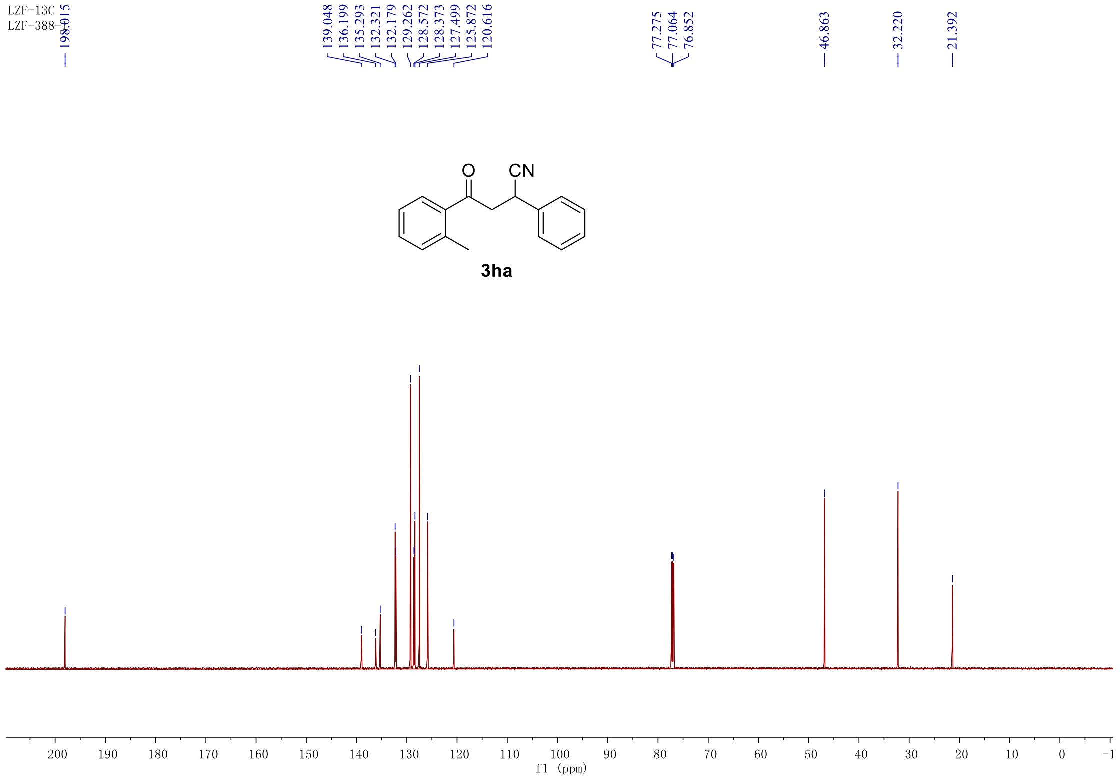
LZF-13C
LZF-388-1
198.015
139.048
136.199
135.293
132.321
132.179
129.262
128.572
128.373
127.499
125.872
120.616
77.275
77.064
76.852
46.863
32.220
21.392
O
CN
3ha
200 190 180 170 160 150 140 130 120 110 100 90 80 70 60 50 40 30 20 10 0 -1
f1 (ppm)

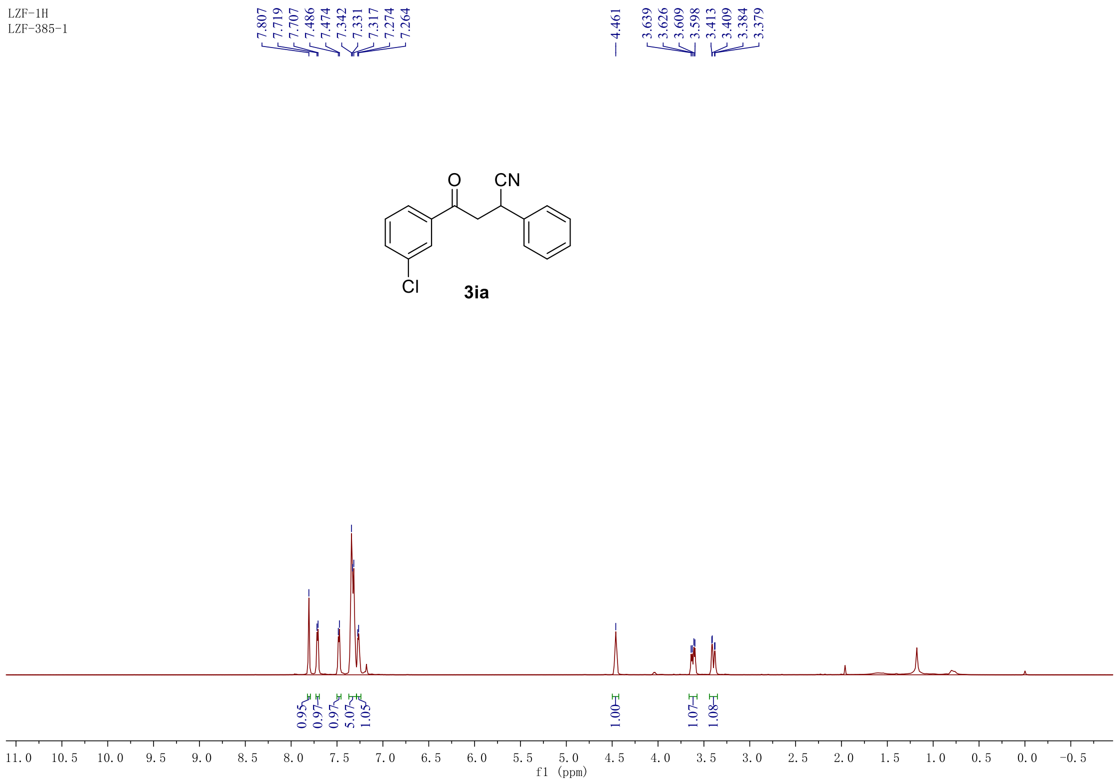
LZF-1H
LZF-385-1
7.807
7.719
7.707
7.486
7.474
7.342
7.331
7.317
7.274
7.264
4.461
3.639
3.626
3.609
3.598
3.413
3.409
3.384
3.379
O
CN
Cl
3ia
0.95
0.97
0.97
5.07
1.05
1.00
1.07
1.08
11.0 10.5 10.0 9.5 9.0 8.5 8.0 7.5 7.0 6.5 6.0 5.5 5.0 4.5 4.0 3.5 3.0 2.5 2.0 1.5 1.0 0.5 0.0 -0.5
f1 (ppm)

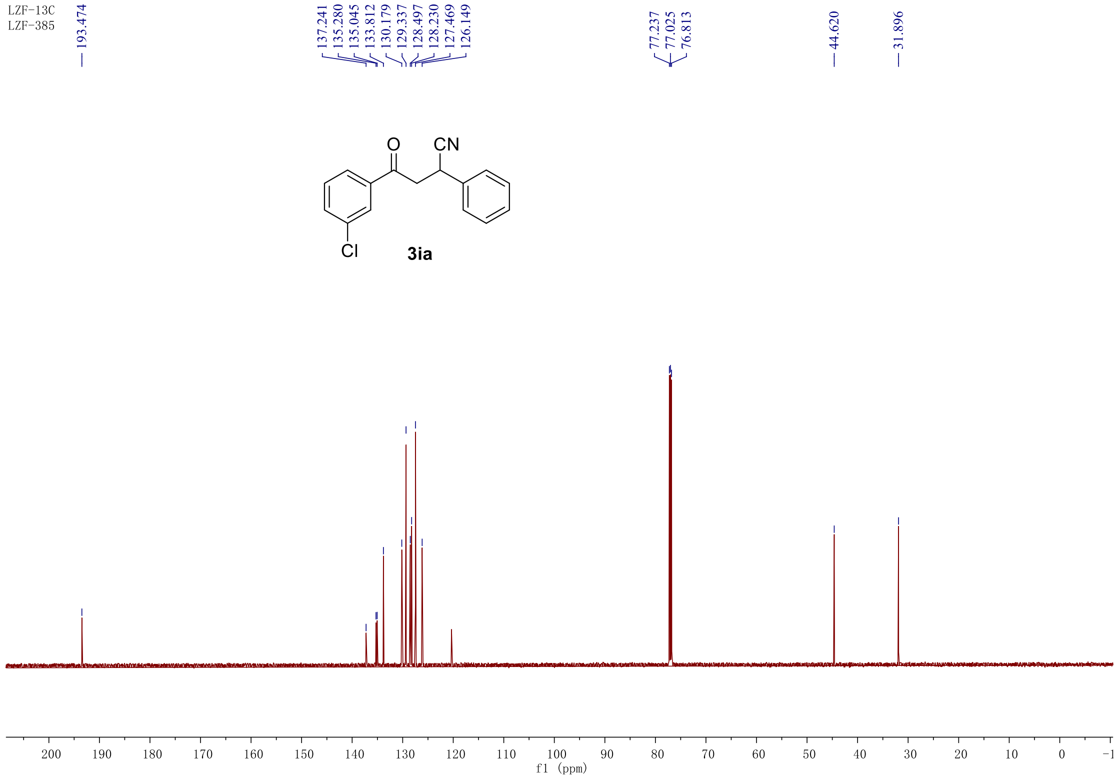
LZF-13C
LZF-385
193.474
137.241
135.280
135.045
133.812
130.179
129.337
128.497
128.230
127.469
126.149
77.237
77.025
76.813
44.620
31.896
O
CN
Cl
3ia
200 190 180 170 160 150 140 130 120 110 100 90 80 70 60 50 40 30 20 10 0 -1
f1 (ppm)

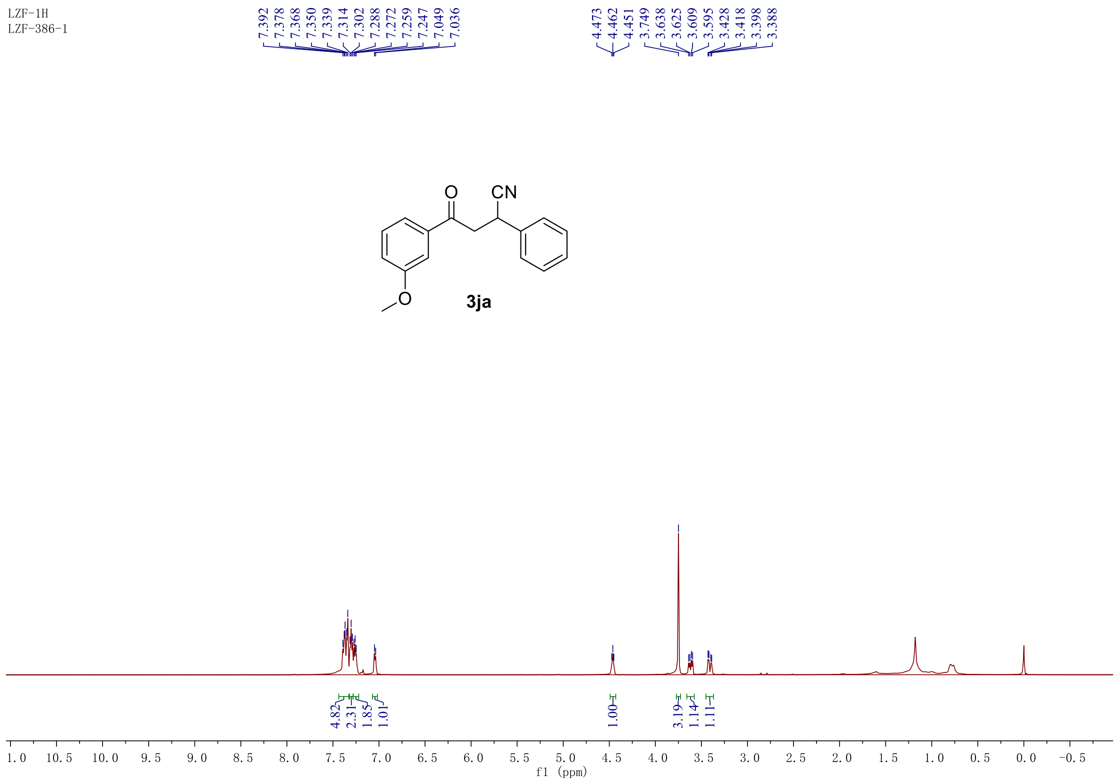
LZF-1H
LZF-386-1
7.392
7.378
7.368
7.350
7.339
7.314
7.302
7.288
7.272
7.259
7.247
7.049
7.036
4.473
4.462
4.451
3.749
3.638
3.625
3.609
3.595
3.428
3.418
3.398
3.388
O
CN
O
3ja
4.82
2.31
1.85
1.01
1.00
3.19
1.14
1.11
f1 (ppm)

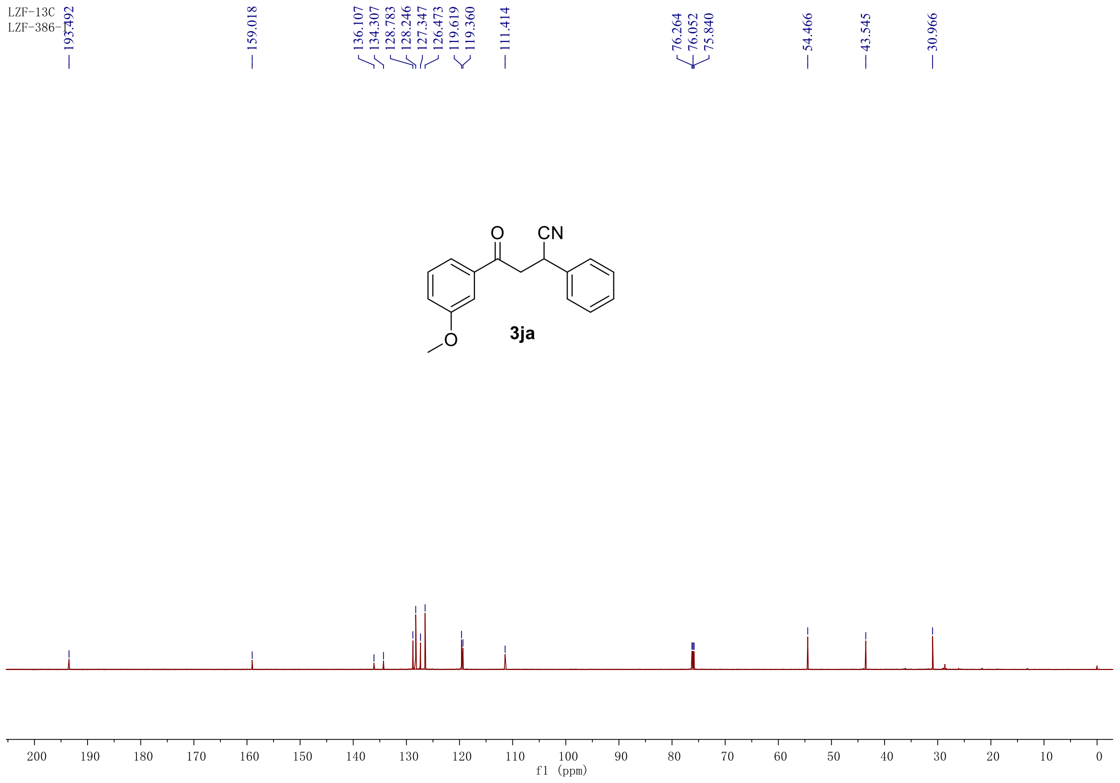
LZF-13C
LZF-386-1
193.492
159.018
136.107
134.307
128.783
128.246
127.347
126.473
119.619
119.360
111.414
76.264
76.052
75.840
54.466
43.545
30.966
O
CN
O
3ja
f1 (ppm)

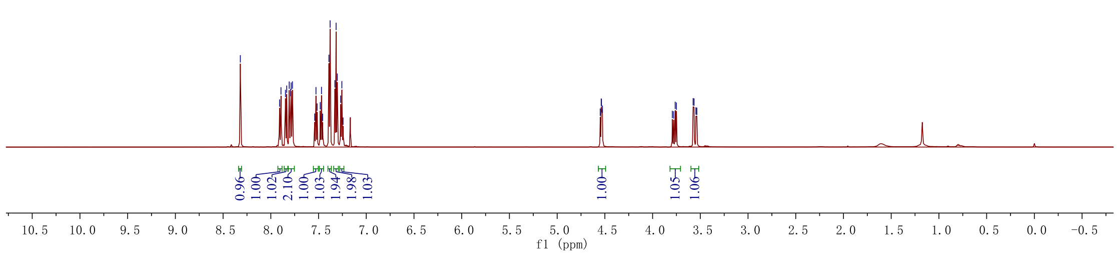
LZF-1H
LZF-431
8.320
7.908
7.894
7.847
7.833
7.807
7.792
7.787
7.773
7.540
7.527
7.515
7.482
7.469
7.391
7.379
7.328
7.316
7.303
7.268
7.256
4.549
4.538
4.536
4.525
3.793
3.780
3.764
3.750
3.575
3.566
3.546
3.536
0.96
1.00
1.02
2.10
1.00
1.03
1.94
1.98
1.03
1.00
1.05
1.06
10.5 10.0 9.5 9.0 8.5 8.0 7.5 7.0 6.5 6.0 5.5 5.0 4.5 4.0 3.5 3.0 2.5 2.0 1.5 1.0 0.5 0.0 -0.5
f1 (ppm)

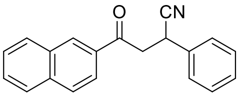
O
CN

3ka

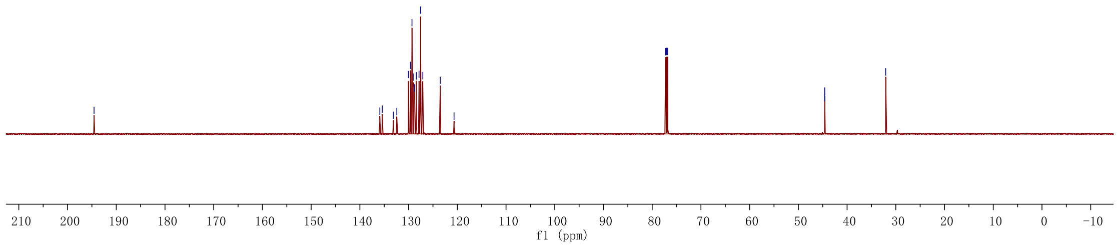
LZF-13C
LZF-431
194.560
135.904
135.403
133.118
132.420
130.027
129.612
129.306
128.942
128.786
128.402
127.861
127.546
127.080
123.504
120.673
77.268
77.057
76.845
44.601
44.584
32.074
210 200 190 180 170 160 150 140 130 120 110 100 90 80 70 60 50 40 30 20 10 0 -10
f1 (ppm)

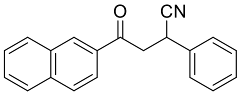
O
CN

3ka

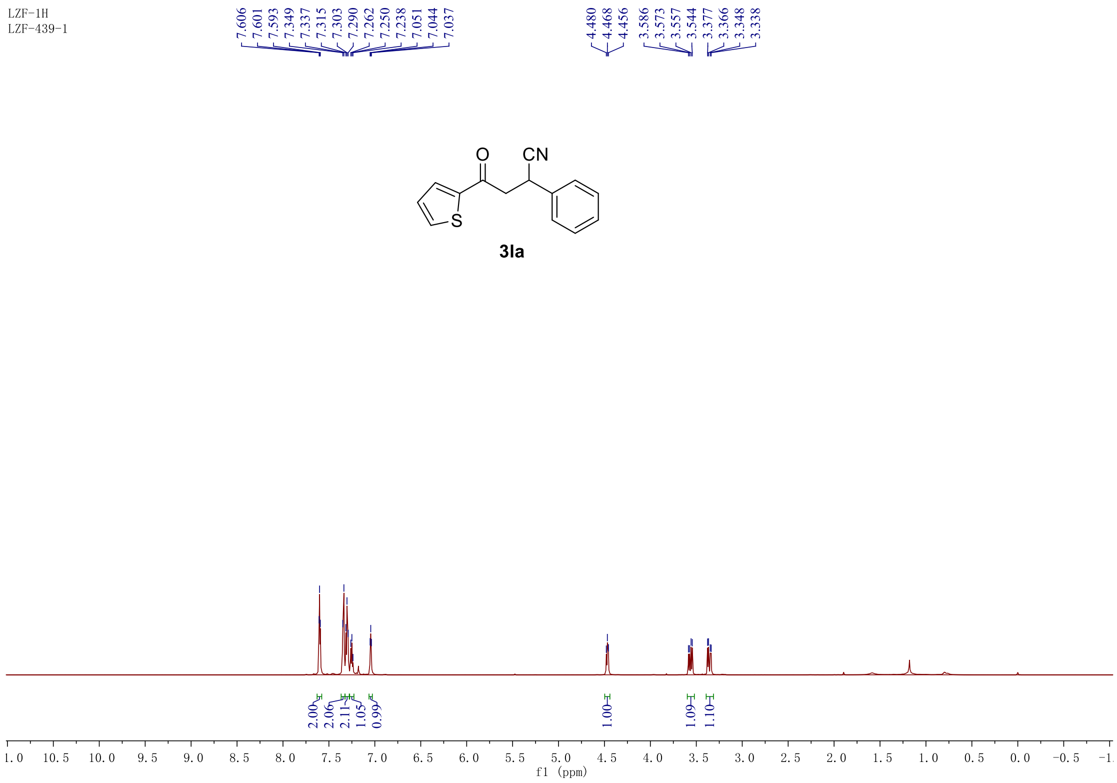
LZF-1H
LZF-439-1
7.606
7.601
7.593
7.349
7.337
7.315
7.303
7.290
7.262
7.250
7.238
7.051
7.044
7.037
4.480
4.468
4.456
3.586
3.573
3.557
3.544
3.377
3.366
3.348
3.338
O
CN
S
3la
2.00
2.06
2.11
1.05
0.99
1.00
1.09
1.10
f1 (ppm)

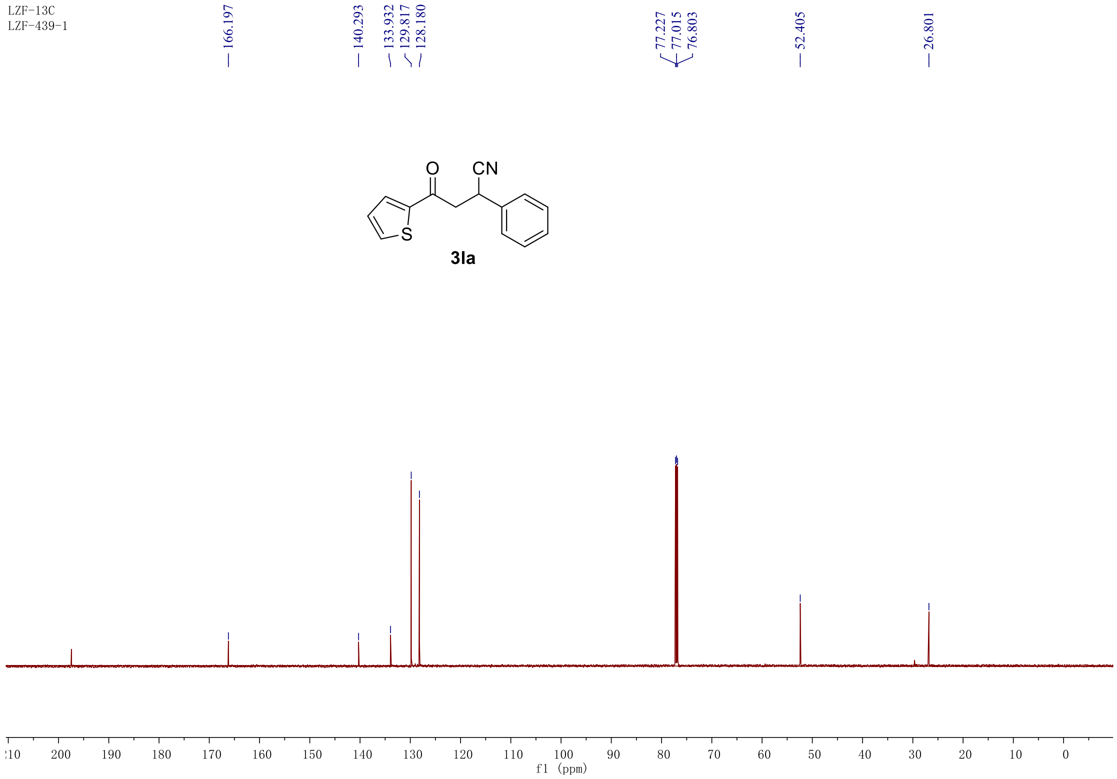
LZF-13C
LZF-439-1
166.197
140.293
133.932
129.817
128.180
77.227
77.015
76.803
52.405
26.801
O
CN
S
3la
f1 (ppm)

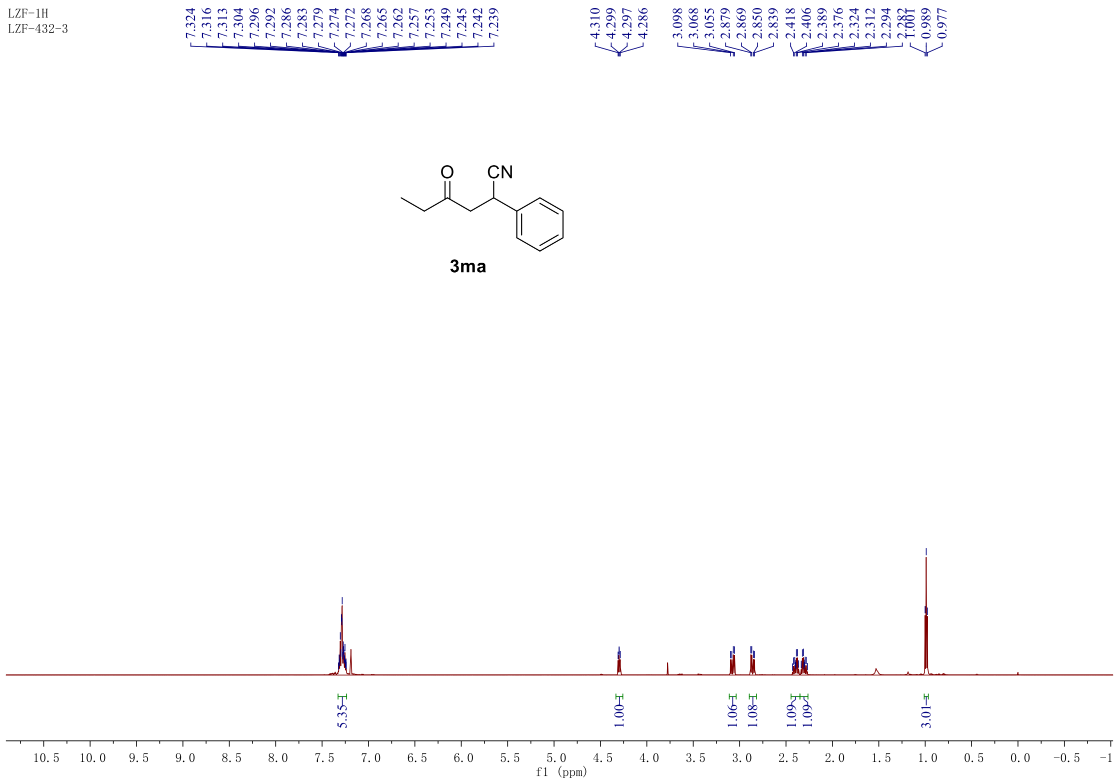
LZF-1H
LZF-432-3
7.324
7.316
7.313
7.304
7.296
7.292
7.286
7.283
7.279
7.274
7.272
7.268
7.265
7.262
7.257
7.253
7.249
7.245
7.242
7.239
4.310
4.299
4.297
4.286
3.098
3.068
3.055
2.879
2.869
2.850
2.839
2.418
2.406
2.389
2.376
2.324
2.312
2.294
2.282
1.001
0.989
0.977
3ma
5.35
1.00
1.06
1.08
1.09
1.09
3.01
f1 (ppm)

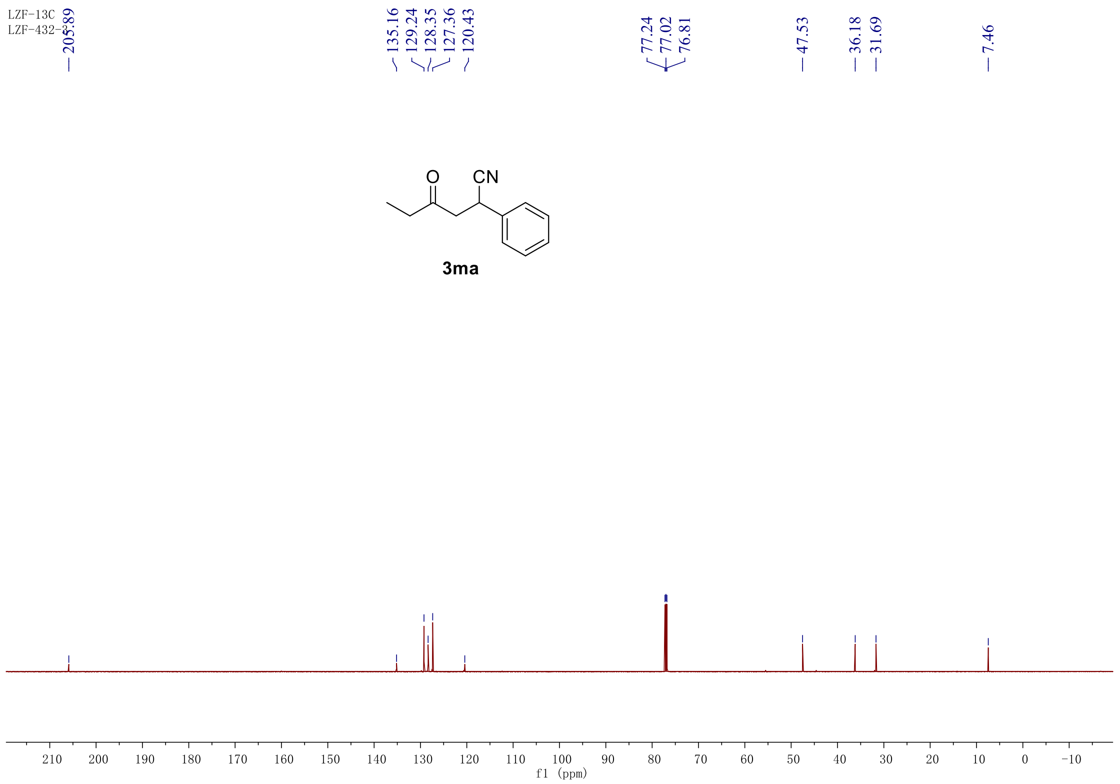
LZF-13C
LZF-432-3
205.89
135.16
129.24
128.35
127.36
120.43
77.24
77.02
76.81
47.53
36.18
31.69
7.46
3ma
f1 (ppm)

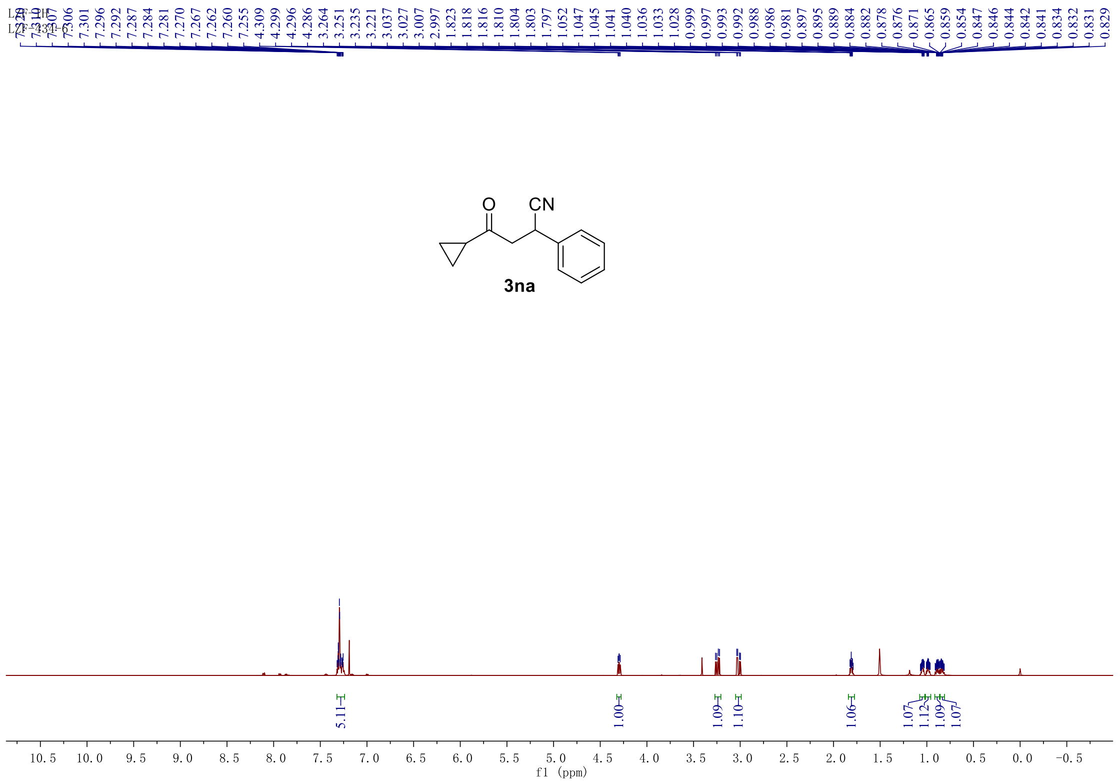
3na
5.11
1.00
1.09
1.10
1.06
1.07
1.12
1.09
1.07
f1 (ppm)

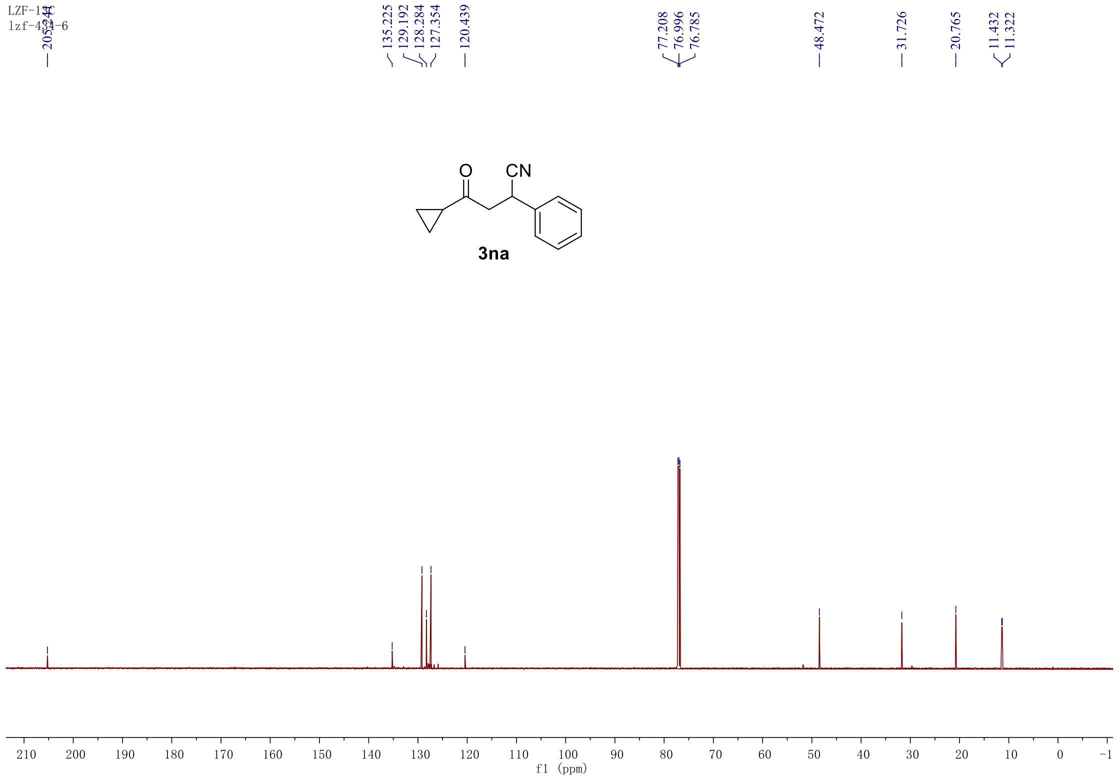
135.225
129.192
128.284
127.354
120.439
77.208
76.996
76.785
48.472
31.726
20.765
11.432
11.322
3na
f1 (ppm)

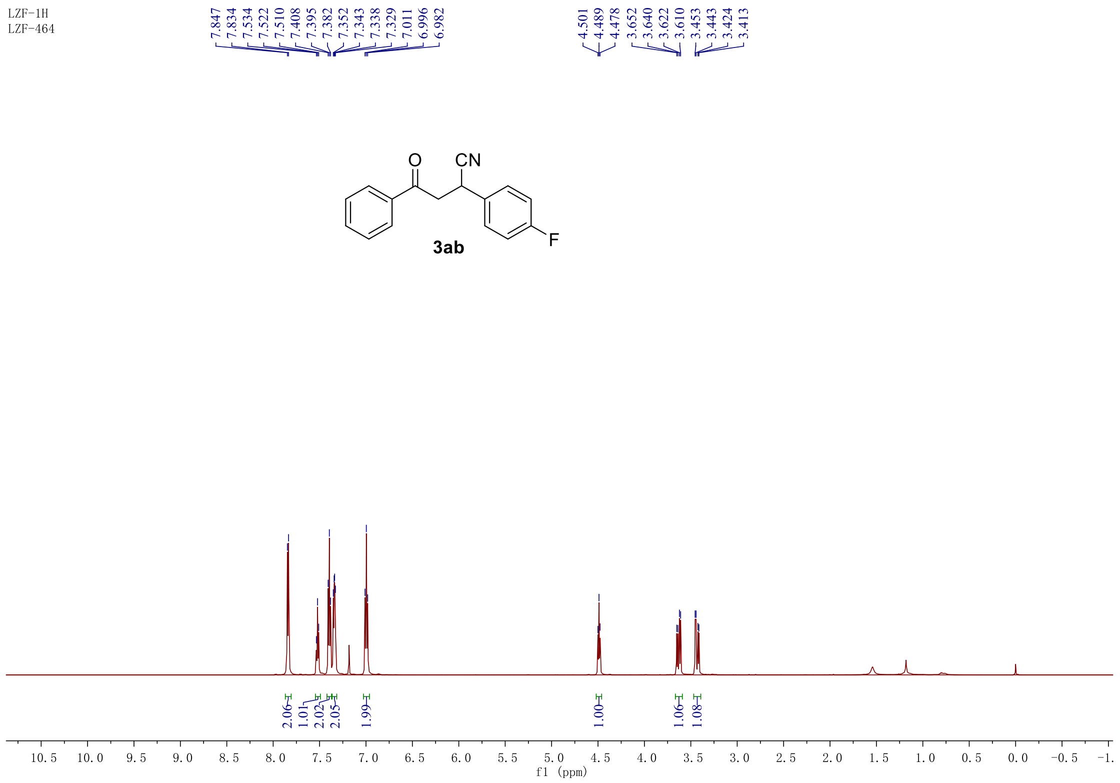
LZF-1H
LZF-464
7.847
7.834
7.534
7.522
7.510
7.408
7.395
7.382
7.352
7.343
7.338
7.329
7.011
6.996
6.982
4.501
4.489
4.478
3.652
3.640
3.622
3.610
3.453
3.443
3.424
3.413
O
CN
F
3ab
2.06
1.01
2.02
2.05
1.99
1.00
1.06
1.08
10.5 10.0 9.5 9.0 8.5 8.0 7.5 7.0 6.5 6.0 5.5 5.0 4.5 4.0 3.5 3.0 2.5 2.0 1.5 1.0 0.5 0.0 -0.5
f1 (ppm)

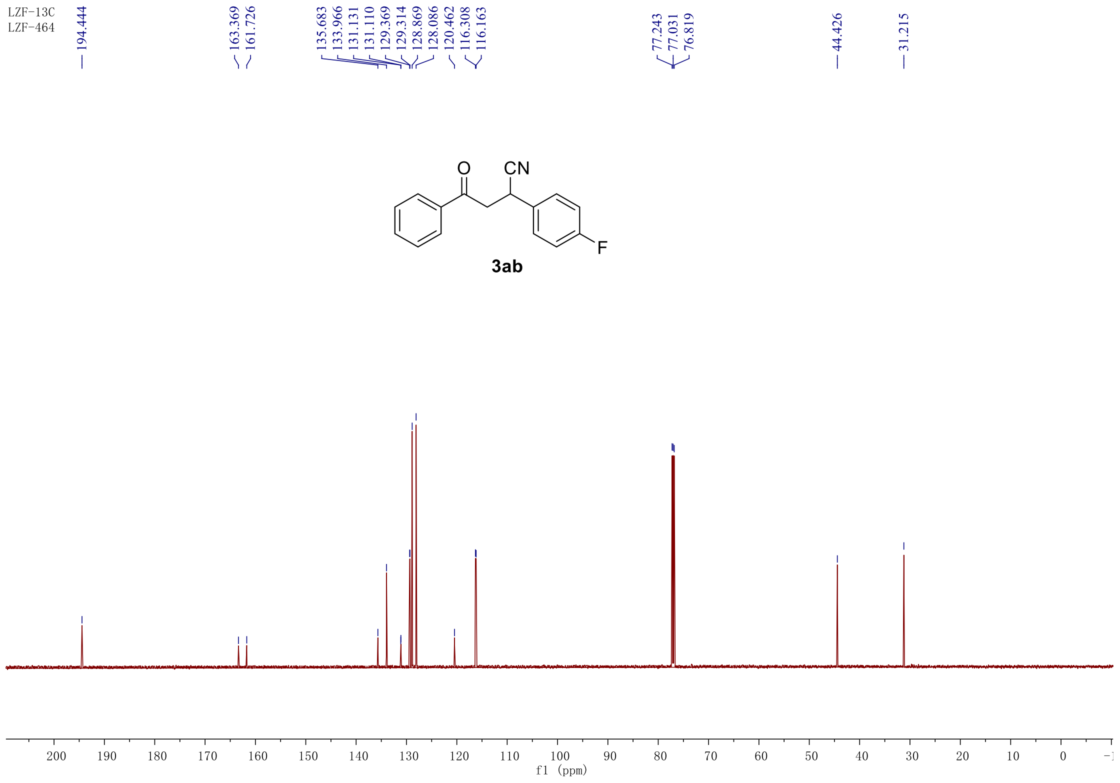
LZF-13C
LZF-464
194.444
163.369
161.726
135.683
133.966
131.131
131.110
129.369
129.314
128.869
128.086
120.462
116.308
116.163
77.243
77.031
76.819
44.426
31.215
O
CN
F
3ab
200 190 180 170 160 150 140 130 120 110 100 90 80 70 60 50 40 30 20 10 0
f1 (ppm)

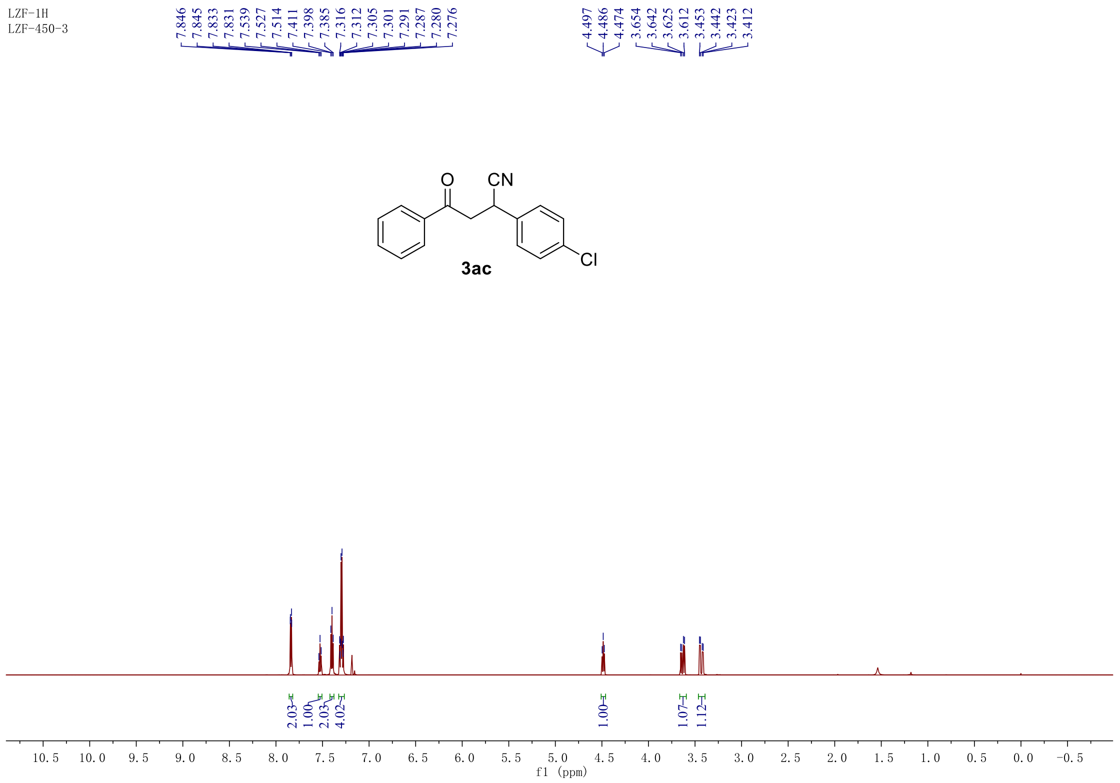
LZF-1H
LZF-450-3
7.846
7.845
7.833
7.831
7.539
7.527
7.514
7.411
7.398
7.385
7.316
7.312
7.305
7.301
7.291
7.287
7.280
7.276
4.497
4.486
4.474
3.654
3.642
3.625
3.612
3.453
3.442
3.423
3.412
O
CN
Cl
3ac
2.03
1.00
2.03
4.02
1.00
1.07
1.12
10.5 10.0 9.5 9.0 8.5 8.0 7.5 7.0 6.5 6.0 5.5 5.0 4.5 4.0 3.5 3.0 2.5 2.0 1.5 1.0 0.5 0.0 -0.5
f1 (ppm)

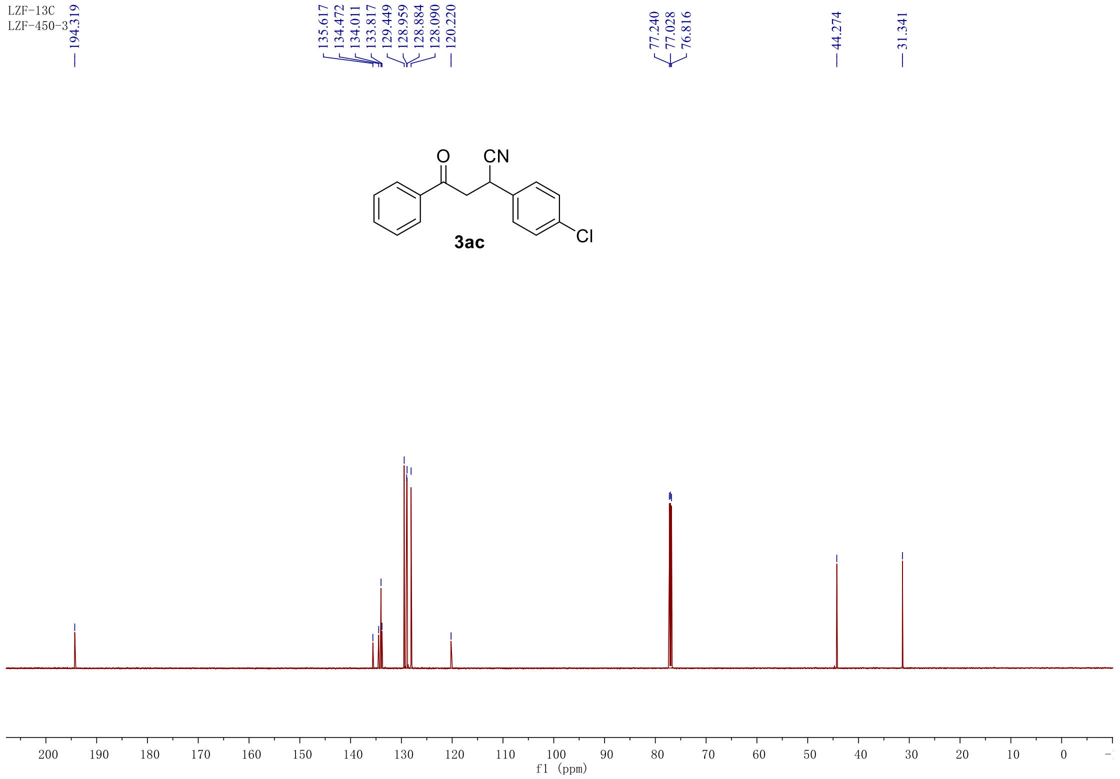
LZF-13C
LZF-450-3
194.319
135.617
134.472
134.011
133.817
129.449
128.959
128.884
128.090
120.220
77.240
77.028
76.816
44.274
31.341
O
CN
Cl
3ac
200 190 180 170 160 150 140 130 120 110 100 90 80 70 60 50 40 30 20 10 0
f1 (ppm)

LZF-1H
LZF-452

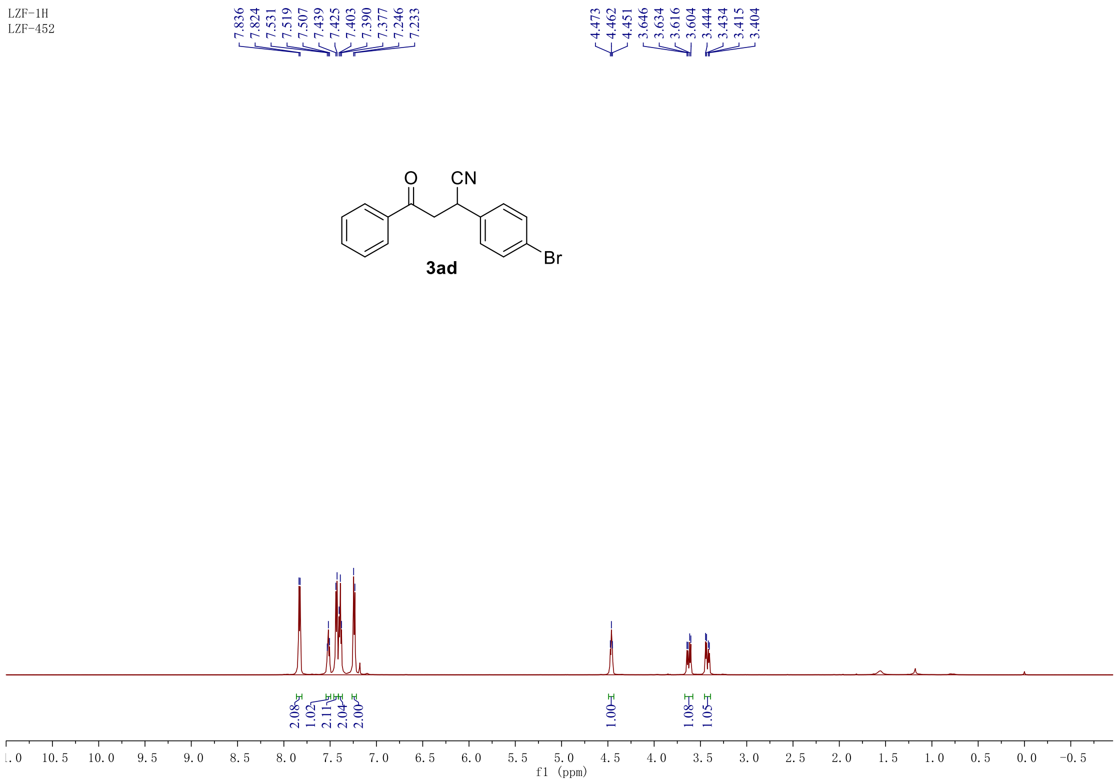

LZF-13C
LZF-452

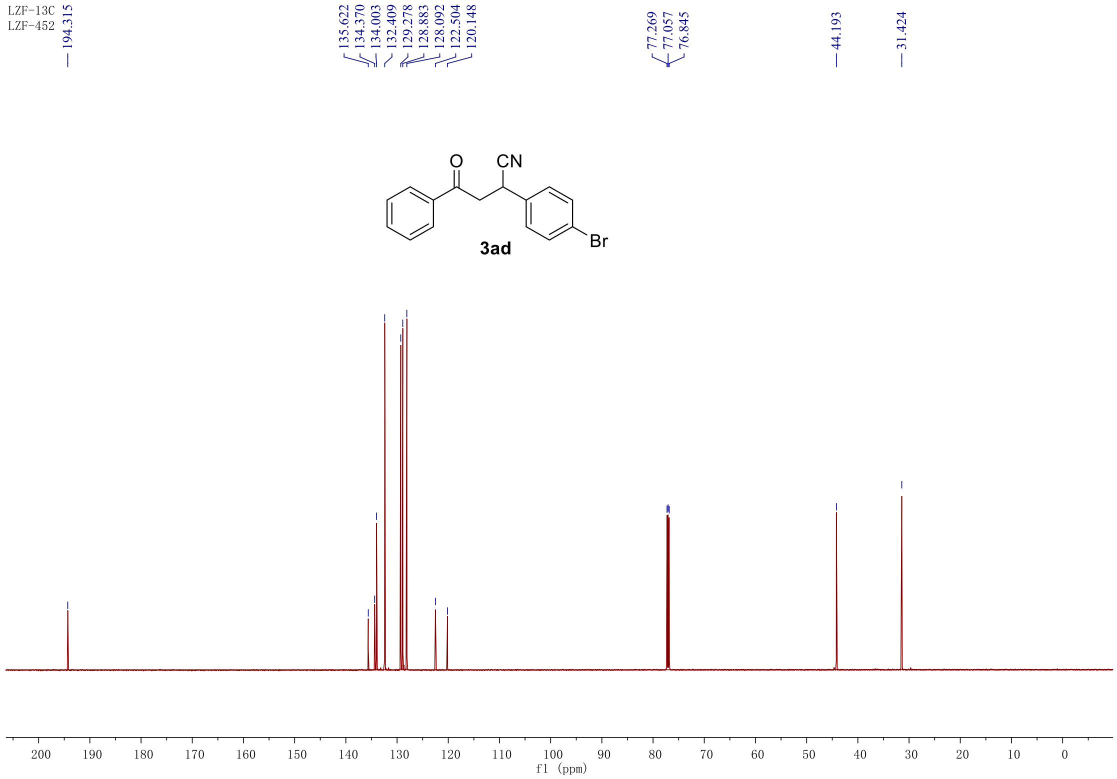

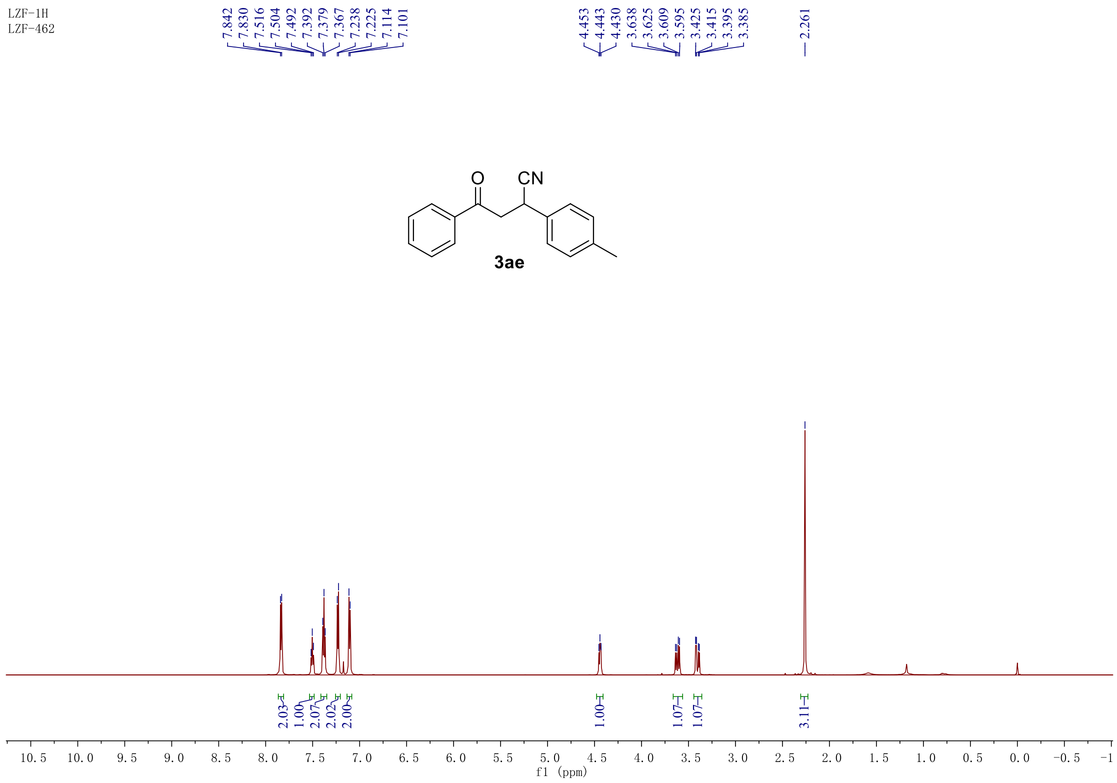
LZF-1H
LZF-462
7.842 7.830 7.516 7.504 7.492 7.392 7.379 7.367 7.238 7.225 7.114 7.101
4.453 4.443 4.430 3.638 3.625 3.609 3.595 3.425 3.415 3.395 3.385
2.261
O
CN
3ae
2.03 1.00 2.07 2.02 2.00
1.00
1.07 1.07
3.11
f1 (ppm)

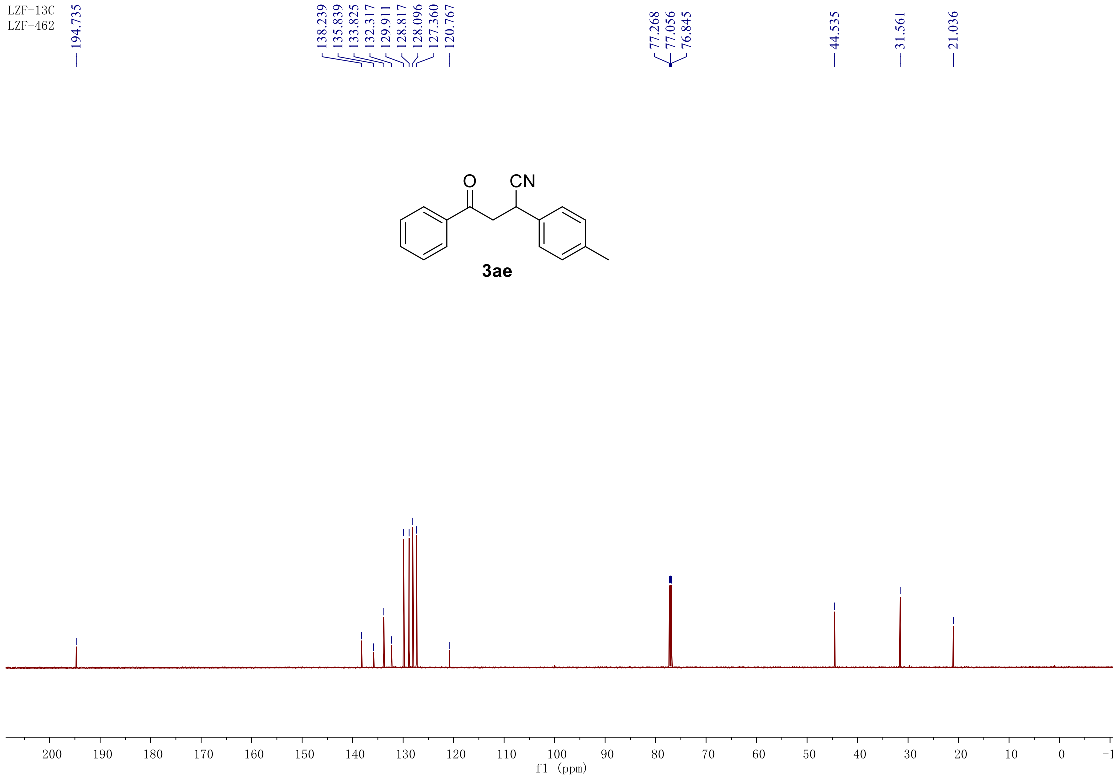
LZF-13C
LZF-462
194.735
138.239 135.839 133.825 132.317 129.911 128.817 128.096 127.360 120.767
77.268 77.056 76.845
44.535
31.561
21.036
O
CN
3ae
f1 (ppm)

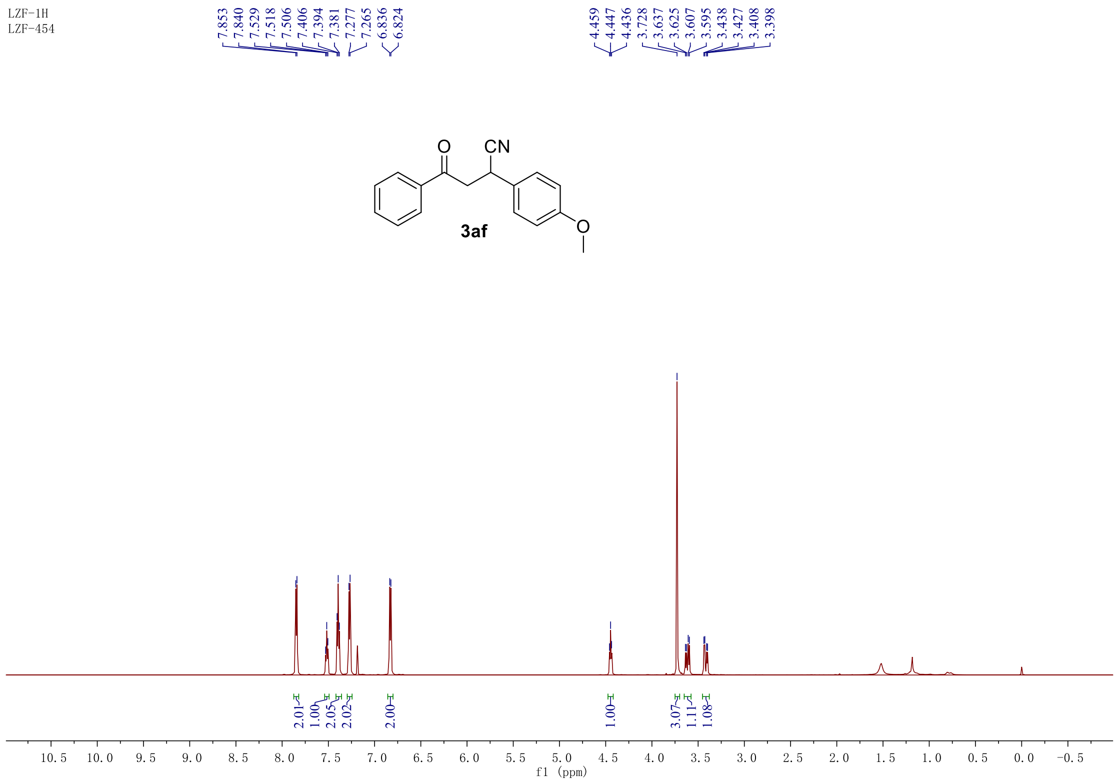
LZF-1H
LZF-454
7.853
7.840
7.529
7.518
7.506
7.406
7.394
7.381
7.277
7.265
6.836
6.824
4.459
4.447
4.436
3.728
3.637
3.625
3.607
3.595
3.438
3.427
3.408
3.398
O
CN
O
3af
2.01
1.00
2.05
2.02
2.00
1.00
3.07
1.11
1.08
10.5 10.0 9.5 9.0 8.5 8.0 7.5 7.0 6.5 6.0 5.5 5.0 4.5 4.0 3.5 3.0 2.5 2.0 1.5 1.0 0.5 0.0 -0.5
f1 (ppm)

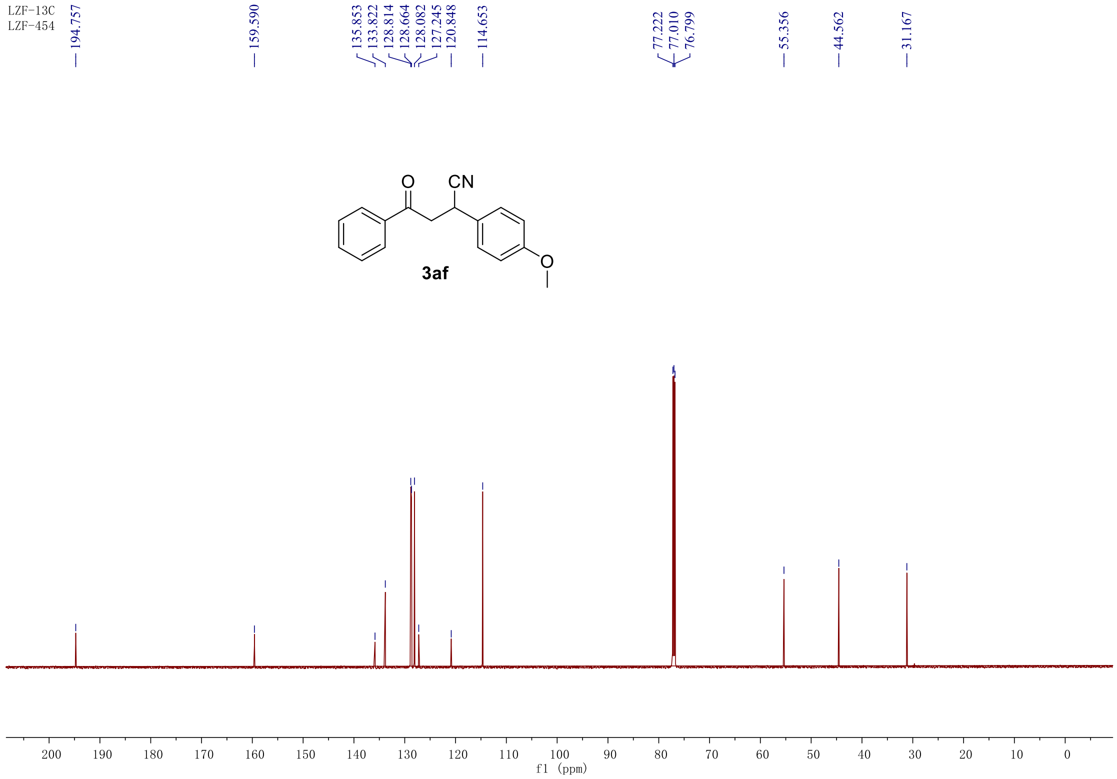
LZF-13C
LZF-454
194.757
159.590
135.853
133.822
128.814
128.664
128.082
127.245
120.848
114.653
77.222
77.010
76.799
55.356
44.562
31.167
O
CN
O
3af
200 190 180 170 160 150 140 130 120 110 100 90 80 70 60 50 40 30 20 10 0
f1 (ppm)

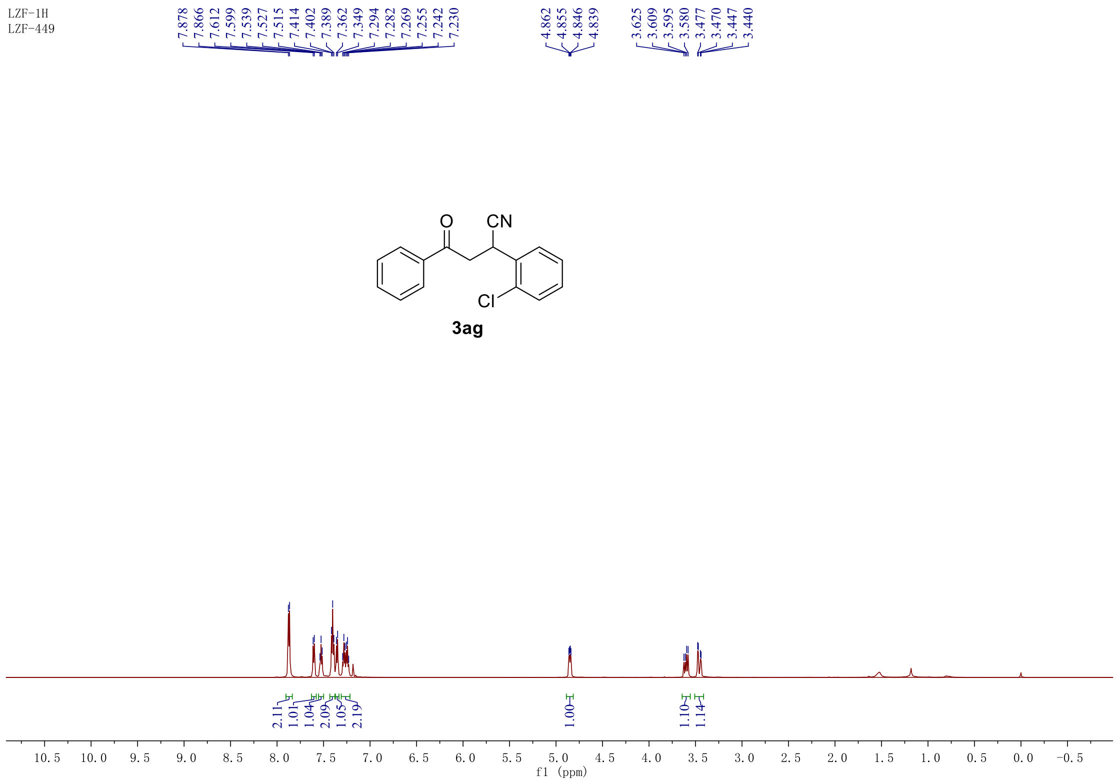
LZF-1H
LZF-449
7.878
7.866
7.612
7.599
7.539
7.527
7.515
7.414
7.402
7.389
7.362
7.349
7.294
7.282
7.269
7.255
7.242
7.230
4.862
4.855
4.846
4.839
3.625
3.609
3.595
3.580
3.477
3.470
3.447
3.440
O
CN
Cl
3ag
2.11
1.01
1.04
2.09
1.05
2.19
1.00
1.10
1.14
10.5 10.0 9.5 9.0 8.5 8.0 7.5 7.0 6.5 6.0 5.5 5.0 4.5 4.0 3.5 3.0 2.5 2.0 1.5 1.0 0.5 0.0 -0.5
f1 (ppm)

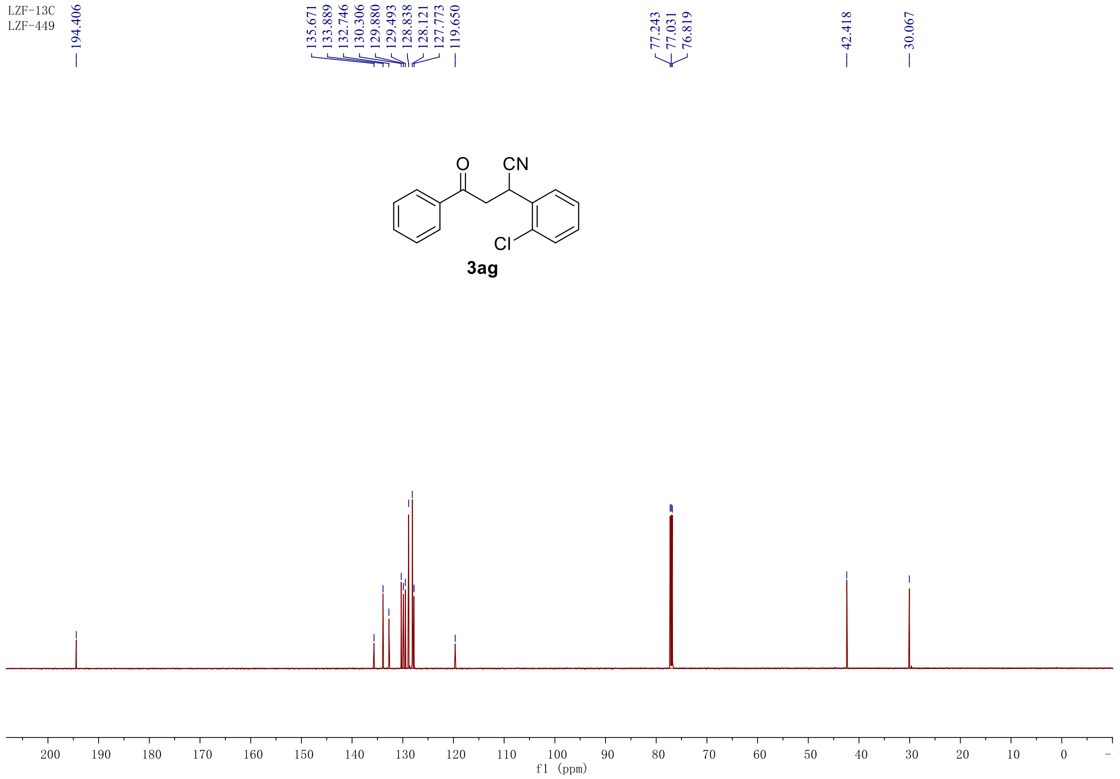
LZF-13C
LZF-449
194.406
135.671
133.889
132.746
130.306
129.880
129.493
128.838
128.121
127.773
119.650
77.243
77.031
76.819
42.418
30.067
O
CN
Cl
3ag
200 190 180 170 160 150 140 130 120 110 100 90 80 70 60 50 40 30 20 10 0
f1 (ppm)

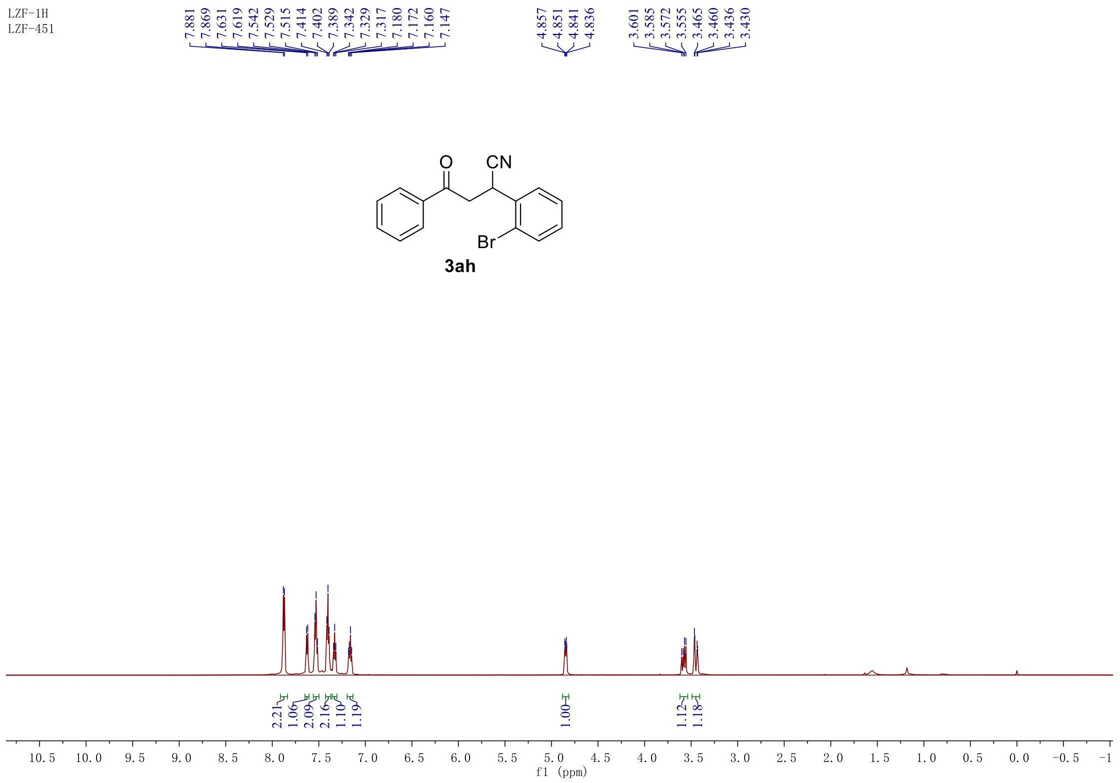
LZF-1H
LZF-451
7.881 7.869 7.631 7.619 7.542 7.529 7.515 7.414 7.402 7.389 7.342 7.329 7.317 7.180 7.172 7.160 7.147
4.857 4.851 4.841 4.836
3.601 3.585 3.572 3.555 3.465 3.460 3.436 3.430
O
CN
Br
3ah
2.21 1.06 2.09 2.16 1.10 1.19
1.00
1.12 1.18
f1 (ppm)

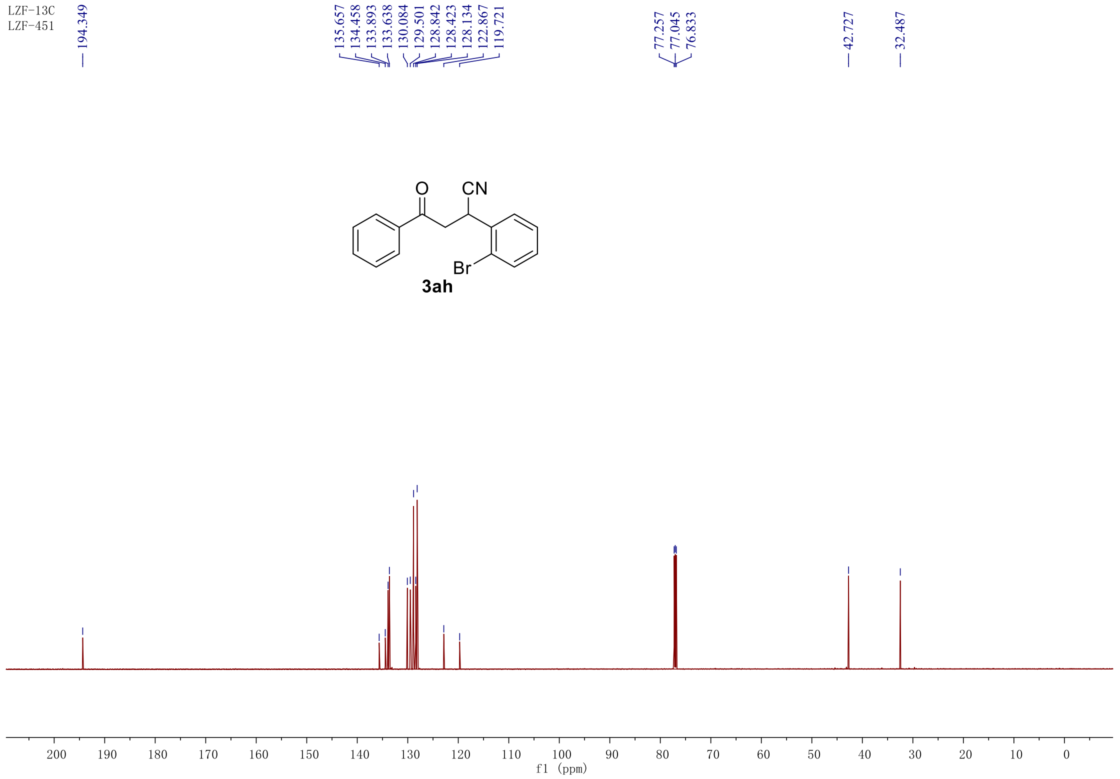
LZF-13C
LZF-451
194.349
135.657 134.458 133.893 133.638 130.084 129.501 128.842 128.423 128.134 122.867 119.721
77.257 77.045 76.833
42.727
32.487
O
CN
Br
3ah
f1 (ppm)

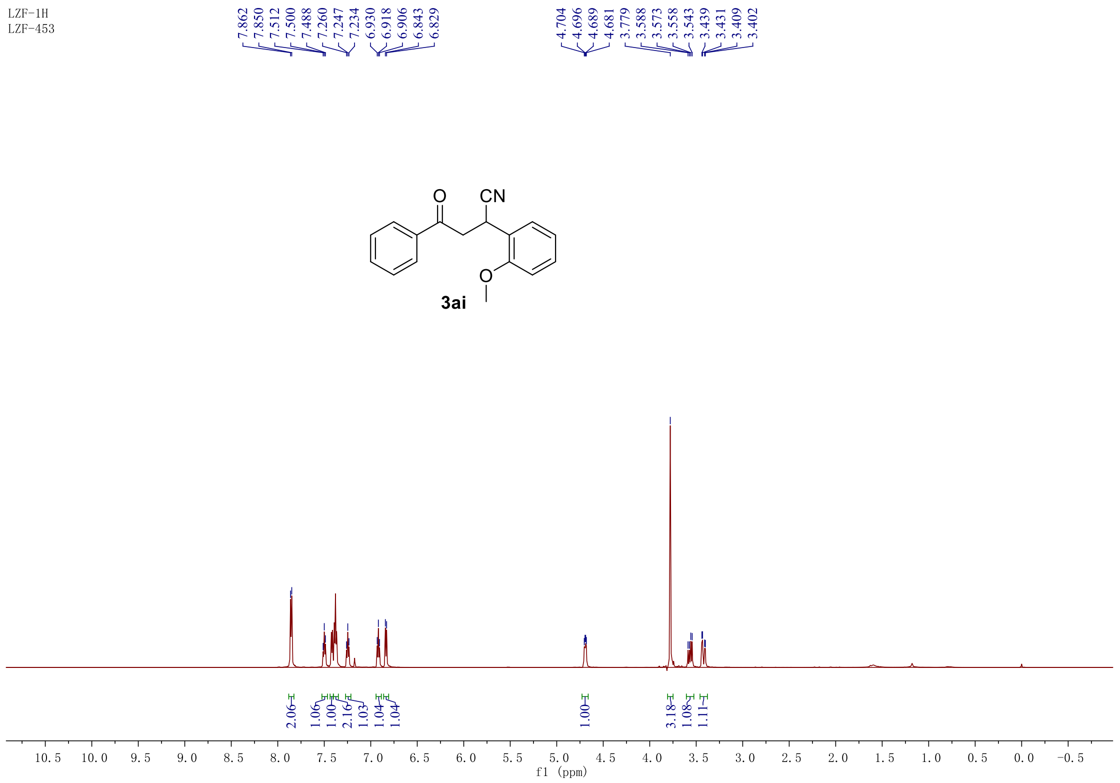
LZF-1H
LZF-453
7.862
7.850
7.512
7.500
7.488
7.260
7.247
7.234
6.930
6.918
6.906
6.843
6.829
4.704
4.696
4.689
4.681
3.779
3.588
3.573
3.558
3.543
3.439
3.431
3.409
3.402
O
CN
3ai
2.06
1.06
1.00
2.16
1.03
1.04
1.04
1.00
3.18
1.08
1.11
10.5 10.0 9.5 9.0 8.5 8.0 7.5 7.0 6.5 6.0 5.5 5.0 4.5 4.0 3.5 3.0 2.5 2.0 1.5 1.0 0.5 0.0 -0.5
f1 (ppm)

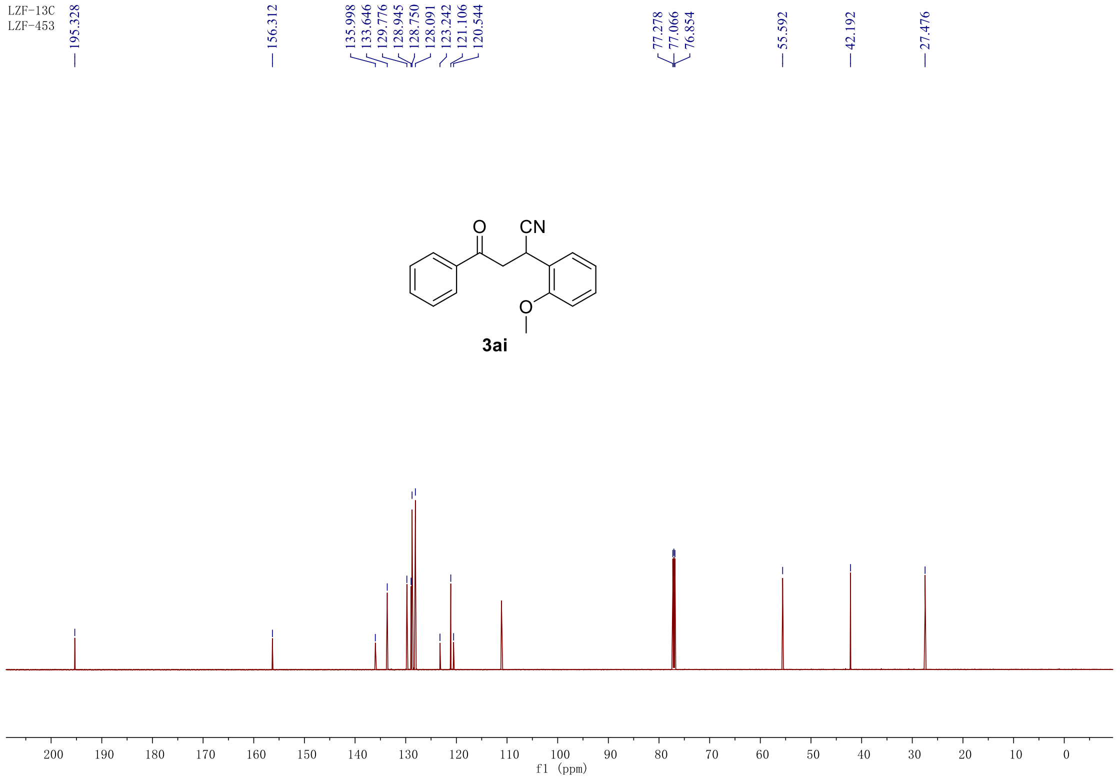
LZF-13C
LZF-453
195.328
156.312
135.998
133.646
129.776
128.945
128.750
128.091
123.242
121.106
120.544
77.278
77.066
76.854
55.592
42.192
27.476
O
CN
3ai
200 190 180 170 160 150 140 130 120 110 100 90 80 70 60 50 40 30 20 10 0
f1 (ppm)

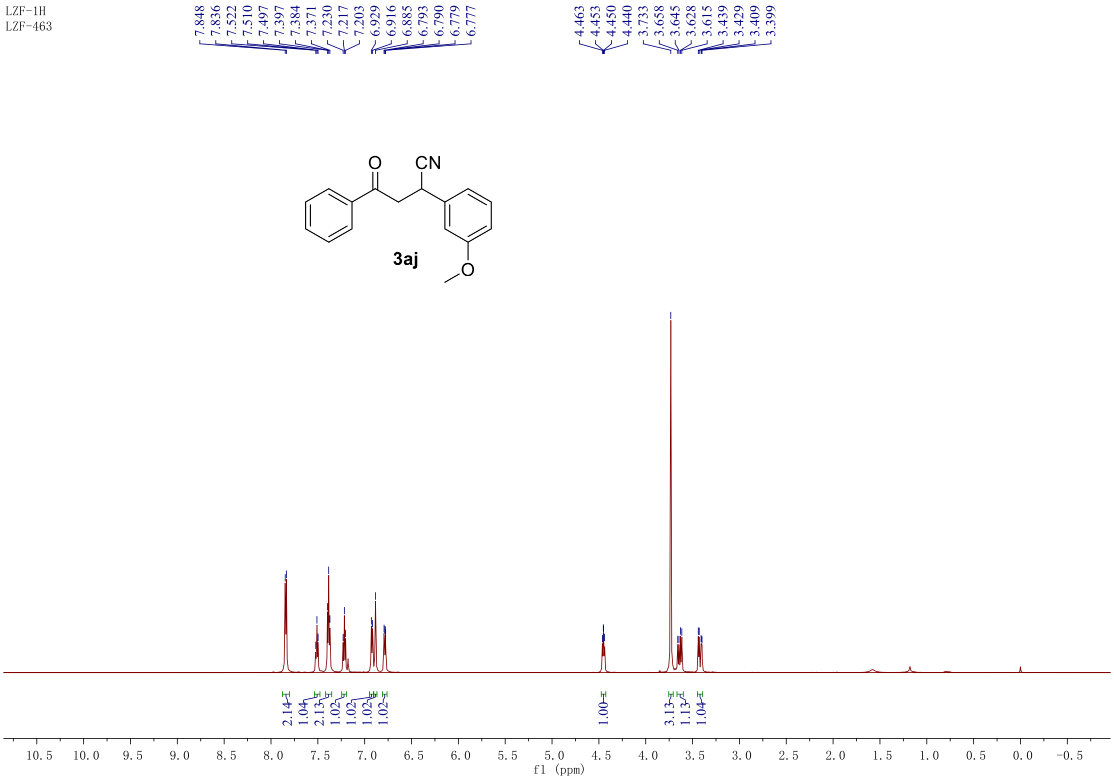
LZF-1H
LZF-463
7.848
7.836
7.522
7.510
7.497
7.397
7.384
7.371
7.230
7.217
7.203
6.929
6.916
6.885
6.793
6.790
6.779
6.777
4.463
4.453
4.450
4.440
3.733
3.658
3.645
3.628
3.615
3.439
3.429
3.409
3.399
O
CN
O
3aj
2.14
1.04
2.13
1.02
1.02
1.02
1.02
1.00
3.13
1.13
1.04
f1 (ppm)

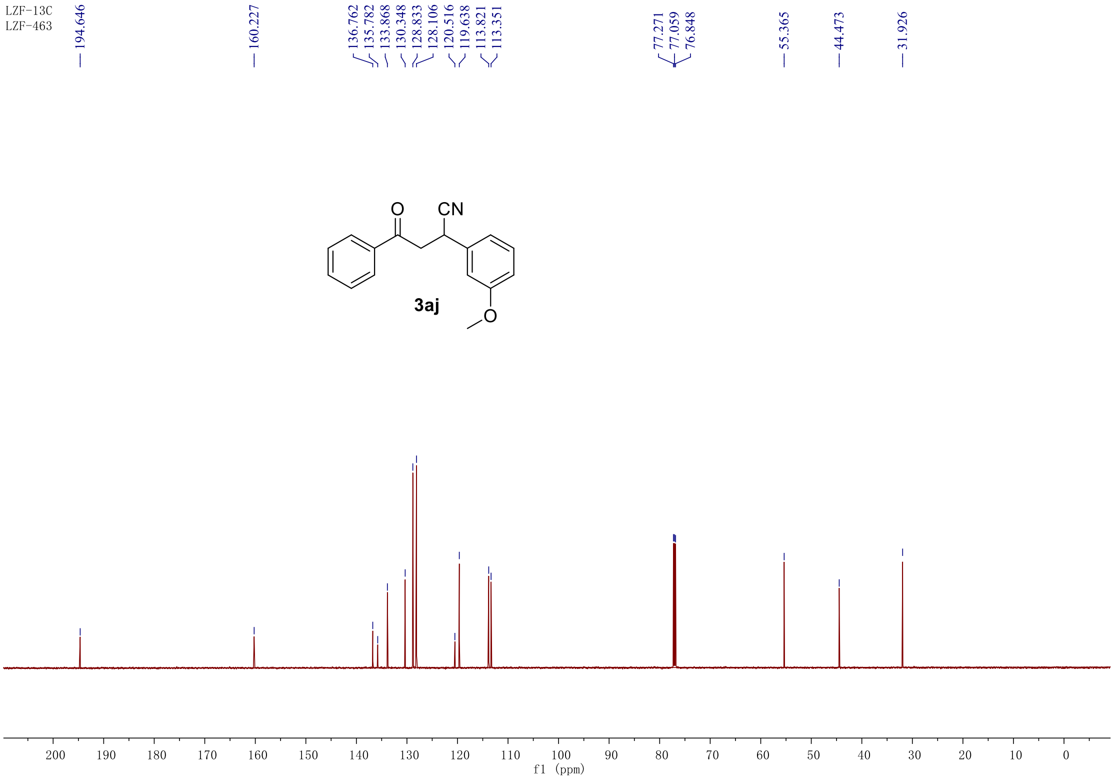
LZF-13C
LZF-463
194.646
160.227
136.762
135.782
133.868
130.348
128.833
128.106
120.516
119.638
113.821
113.351
77.271
77.059
76.848
55.365
44.473
31.926
O
CN
O
3aj
f1 (ppm)

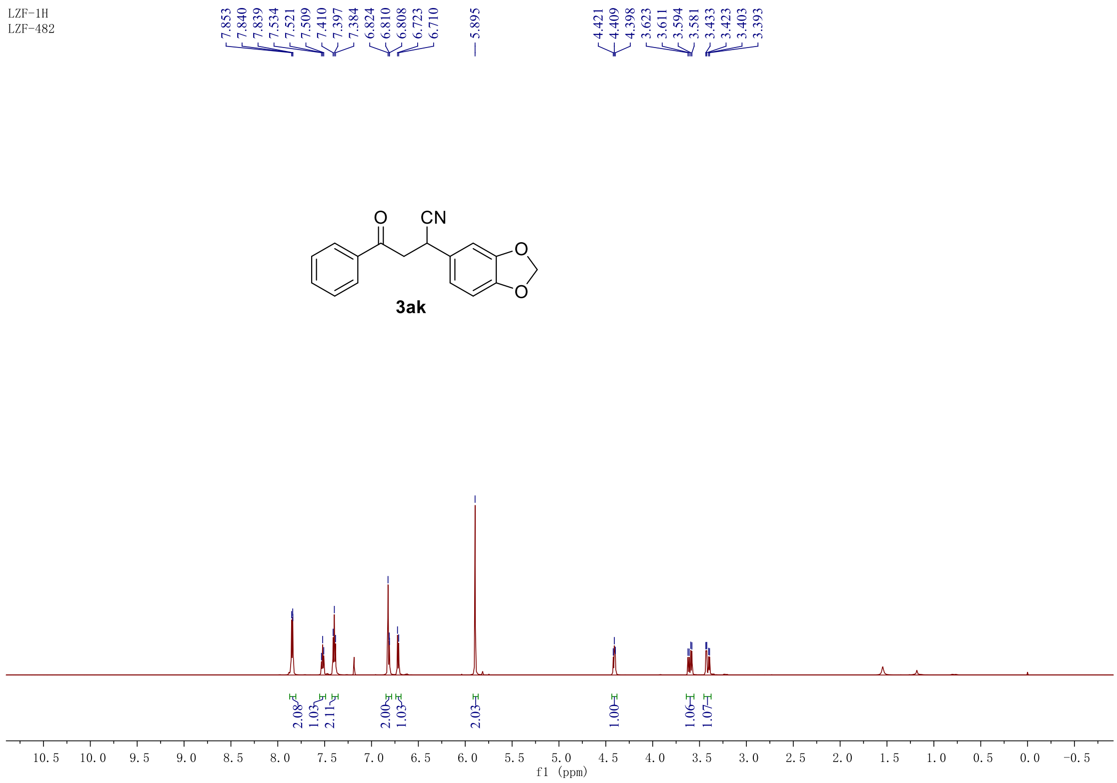
LZF-1H
LZF-482
7.853
7.840
7.839
7.534
7.521
7.509
7.410
7.397
7.384
6.824
6.810
6.808
6.723
6.710
5.895
4.421
4.409
4.398
3.623
3.611
3.594
3.581
3.433
3.423
3.403
3.393
O
CN
O
O
3ak
2.08
1.03
2.11
2.00
1.03
2.03
1.00
1.06
1.07
10.5 10.0 9.5 9.0 8.5 8.0 7.5 7.0 6.5 6.0 5.5 5.0 4.5 4.0 3.5 3.0 2.5 2.0 1.5 1.0 0.5 0.0 -0.5
f1 (ppm)

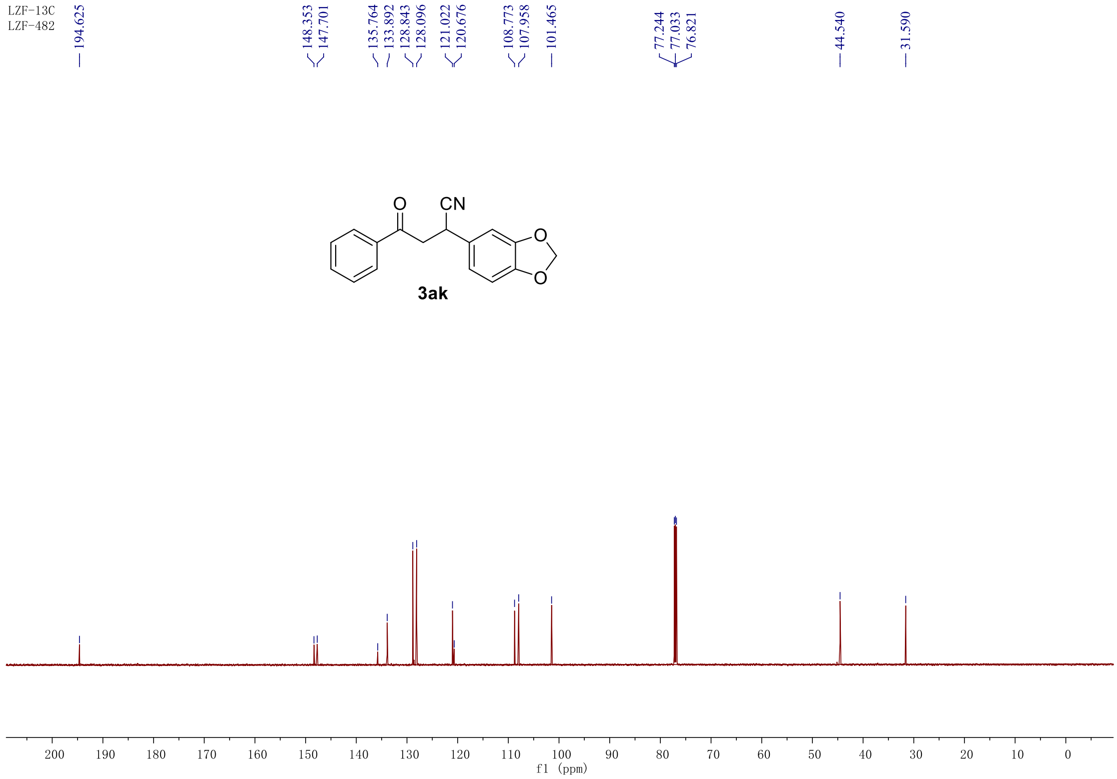
LZF-13C
LZF-482
194.625
148.353
147.701
135.764
133.892
128.843
128.096
121.022
120.676
108.773
107.958
101.465
77.244
77.033
76.821
44.540
31.590
O
CN
O
O
3ak
200 190 180 170 160 150 140 130 120 110 100 90 80 70 60 50 40 30 20 10 0
f1 (ppm)

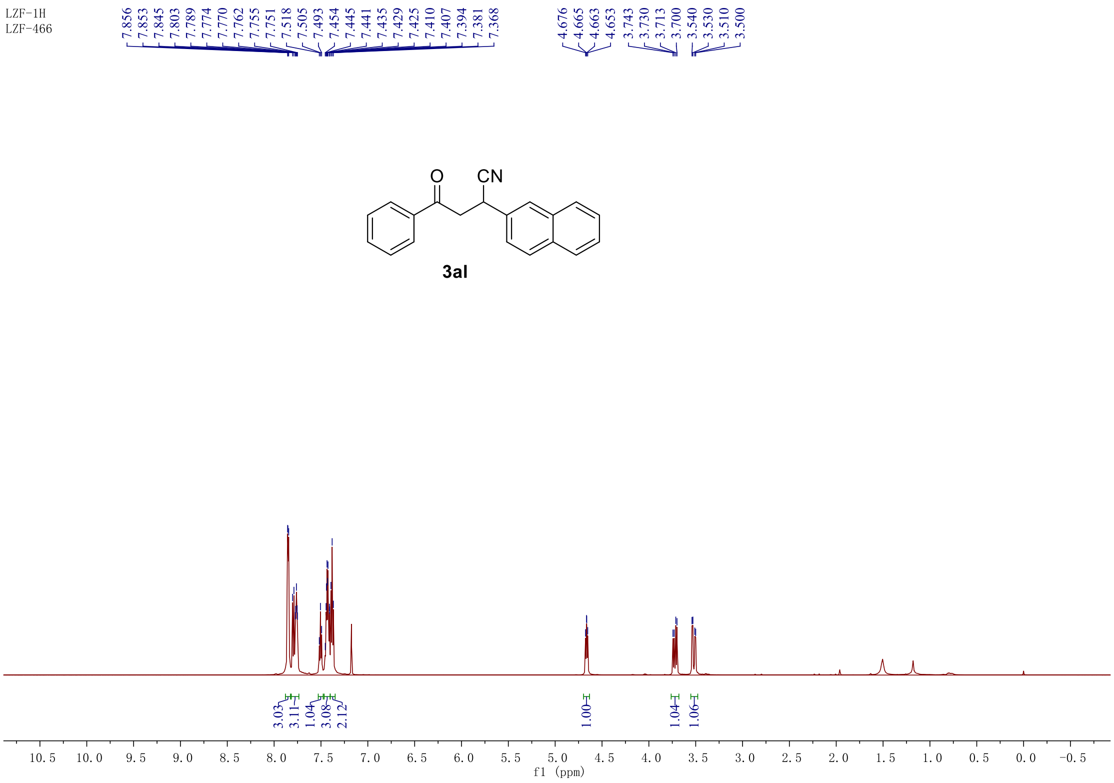
LZF-1H
LZF-466
7.856 7.853 7.845 7.803 7.789 7.774 7.770 7.762 7.755 7.751 7.518 7.505 7.493 7.454 7.445 7.441 7.435 7.429 7.425 7.410 7.407 7.394 7.381 7.368
4.676 4.665 4.663 4.653 3.743 3.730 3.713 3.700 3.540 3.530 3.510 3.500
O
CN
3.03 3.11 1.04 3.08 2.12 1.00 1.04 1.06
10.5 10.0 9.5 9.0 8.5 8.0 7.5 7.0 6.5 6.0 5.5 5.0 4.5 4.0 3.5 3.0 2.5 2.0 1.5 1.0 0.5 0.0 -0.5
f1 (ppm)

3al

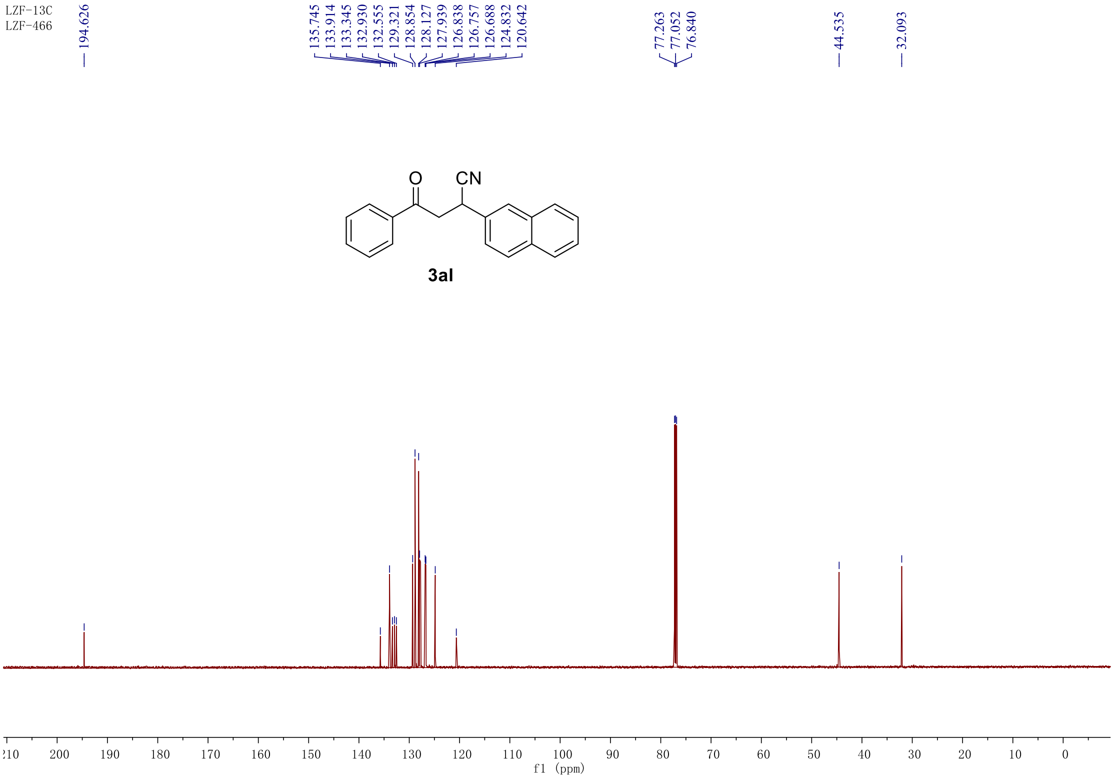
LZF-13C
LZF-466
194.626
135.745 133.914 133.345 132.930 132.555 129.321 128.854 128.127 127.939 126.838 126.757 126.688 124.832 120.642
77.263 77.052 76.840
44.535
32.093
O
CN
210 200 190 180 170 160 150 140 130 120 110 100 90 80 70 60 50 40 30 20 10 0
f1 (ppm)

3al

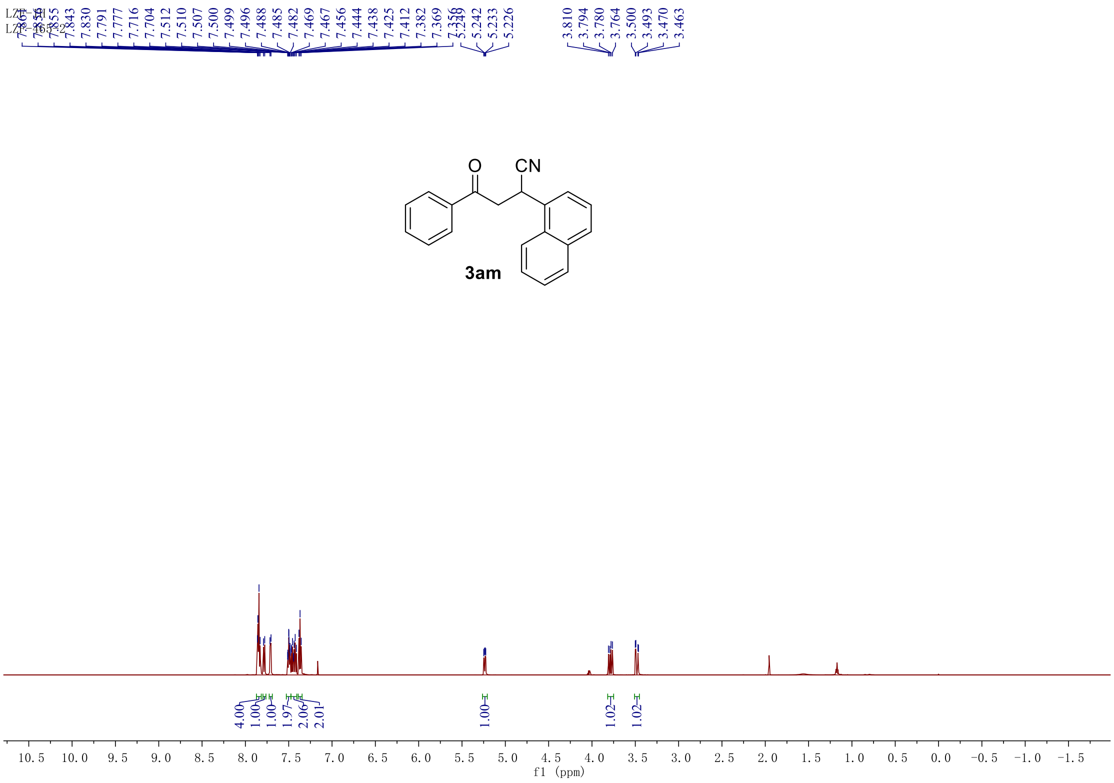
7.855
7.843
7.830
7.791
7.777
7.716
7.704
7.512
7.510
7.507
7.500
7.499
7.496
7.488
7.485
7.482
7.469
7.467
7.456
7.444
7.438
7.425
7.412
7.382
7.369
5.242
5.233
5.226
3.810
3.794
3.780
3.764
3.500
3.493
3.470
3.463
O
CN
3am
4.00
1.00
1.00
1.97
2.06
2.01
1.00
1.02
1.02
f1 (ppm)

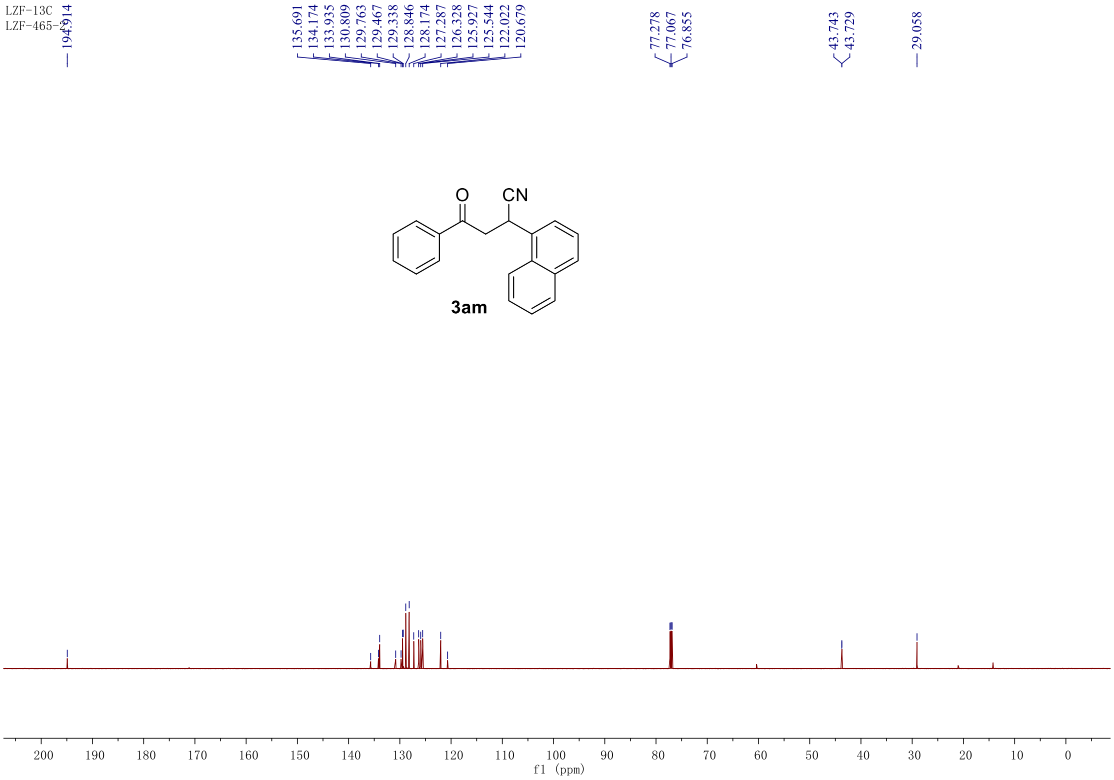
LZF-13C
LZF-465-2
194.914
135.691
134.174
133.935
130.809
129.763
129.467
129.338
128.846
128.174
127.287
126.328
125.927
125.544
122.022
120.679
77.278
77.067
76.855
43.743
43.729
29.058
O
CN
3am
f1 (ppm)

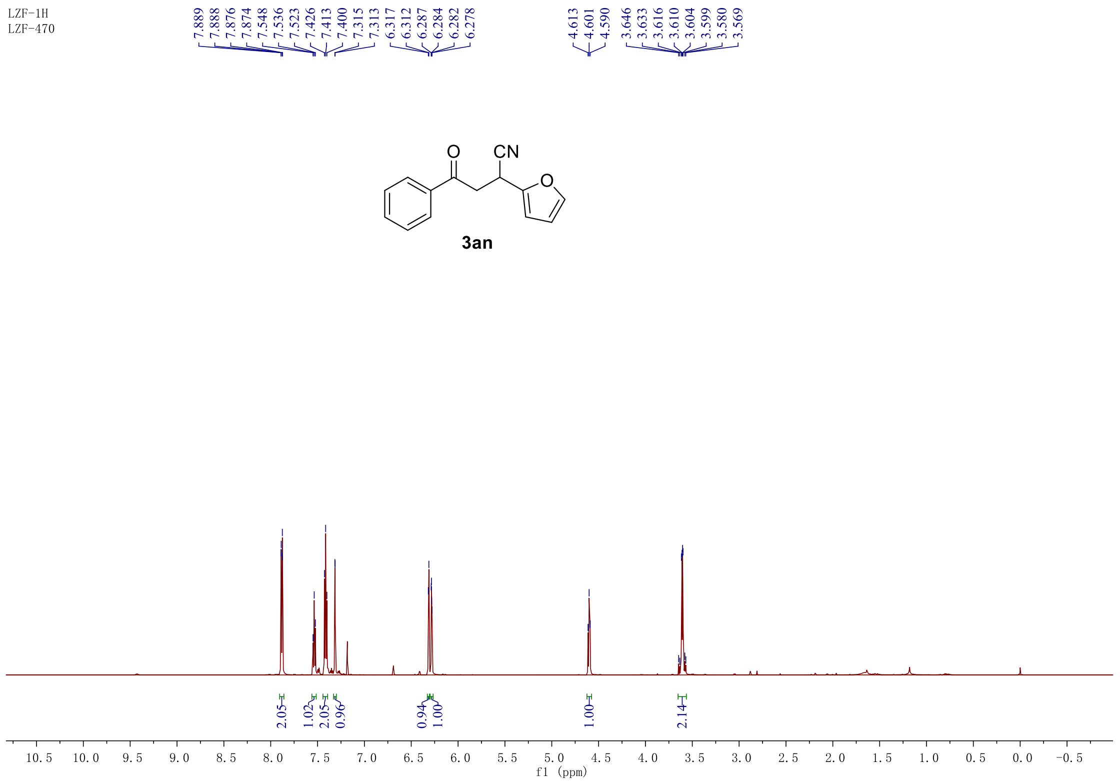
LZF-1H
LZF-470
7.889 7.888 7.876 7.874 7.548 7.536 7.523 7.426 7.413 7.400 7.315 7.313 6.317 6.312 6.287 6.284 6.282 6.278
4.613 4.601 4.590 3.646 3.633 3.616 3.610 3.604 3.599 3.580 3.569
O
CN
O
3an
2.05 1.02 2.05 0.96 0.94 1.00 1.00 2.14
f1 (ppm)

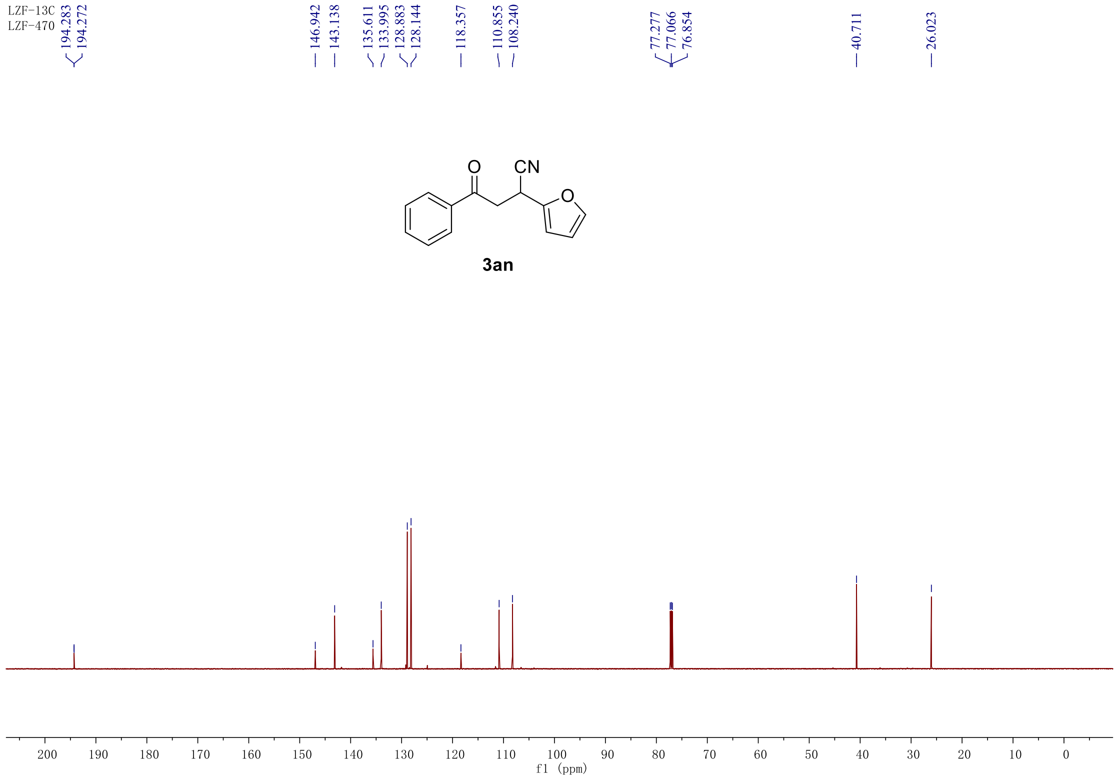
LZF-13C
LZF-470
194.283 194.272
146.942 143.138 135.611 133.995 128.883 128.144 118.357 110.855 108.240
77.277 77.066 76.854
40.711
26.023
O
CN
O
3an
f1 (ppm)

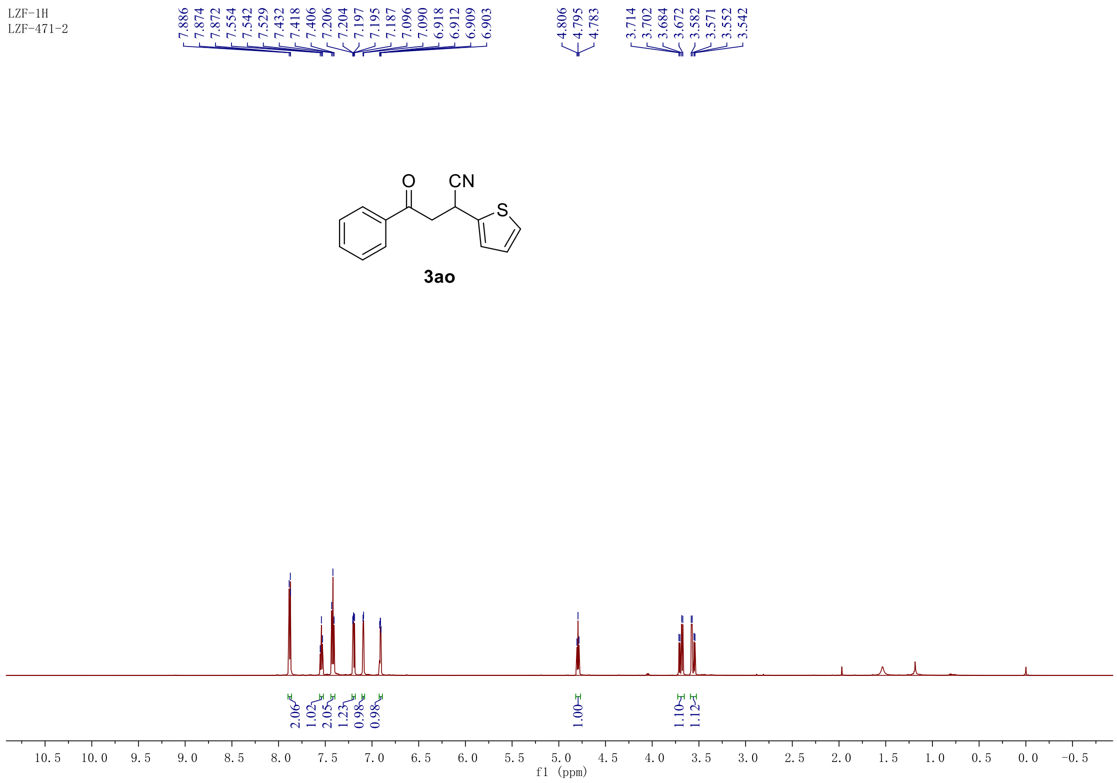
LZF-1H
LZF-471-2
7.886
7.874
7.872
7.554
7.542
7.529
7.432
7.418
7.406
7.206
7.204
7.197
7.195
7.187
7.096
7.090
6.918
6.912
6.909
6.903
4.806
4.795
4.783
3.714
3.702
3.684
3.672
3.582
3.571
3.552
3.542
O
CN
S
3ao
2.06
1.02
2.05
1.23
0.98
0.98
1.00
1.10
1.12
10.5 10.0 9.5 9.0 8.5 8.0 7.5 7.0 6.5 6.0 5.5 5.0 4.5 4.0 3.5 3.0 2.5 2.0 1.5 1.0 0.5 0.0 -0.5
f1 (ppm)

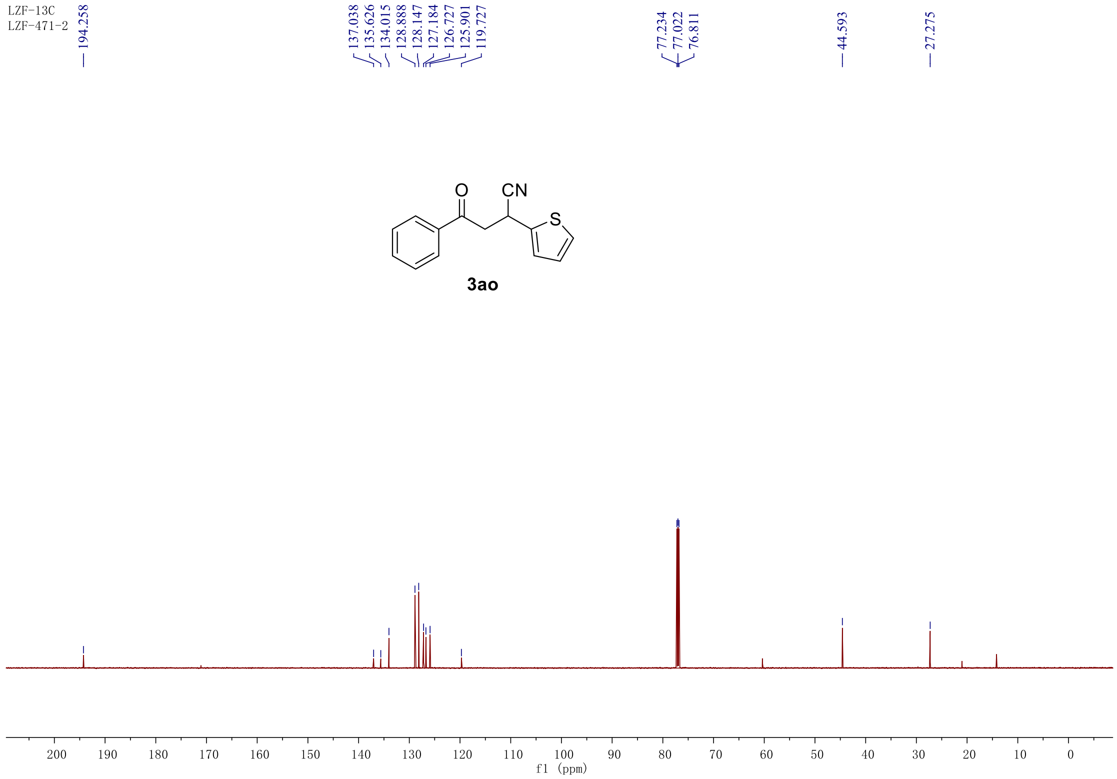
LZF-13C
LZF-471-2
194.258
137.038
135.626
134.015
128.888
128.147
127.184
126.727
125.901
119.727
77.234
77.022
76.811
44.593
27.275
O
CN
S
3ao
200 190 180 170 160 150 140 130 120 110 100 90 80 70 60 50 40 30 20 10 0
f1 (ppm)

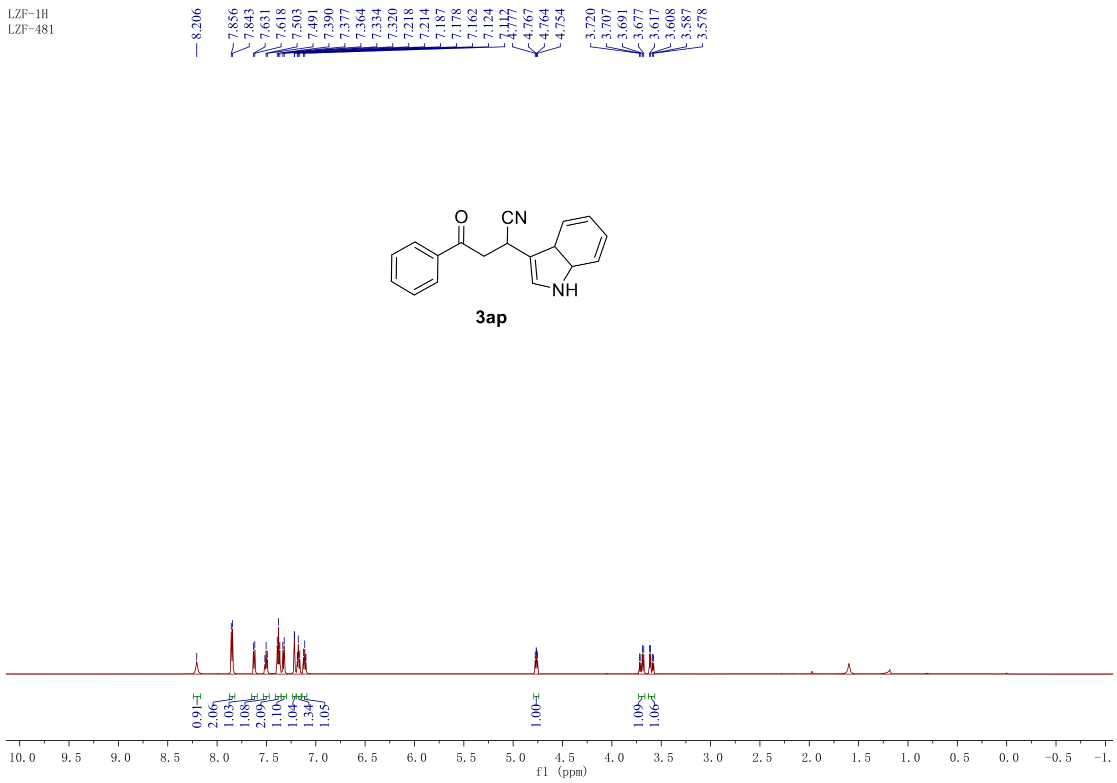

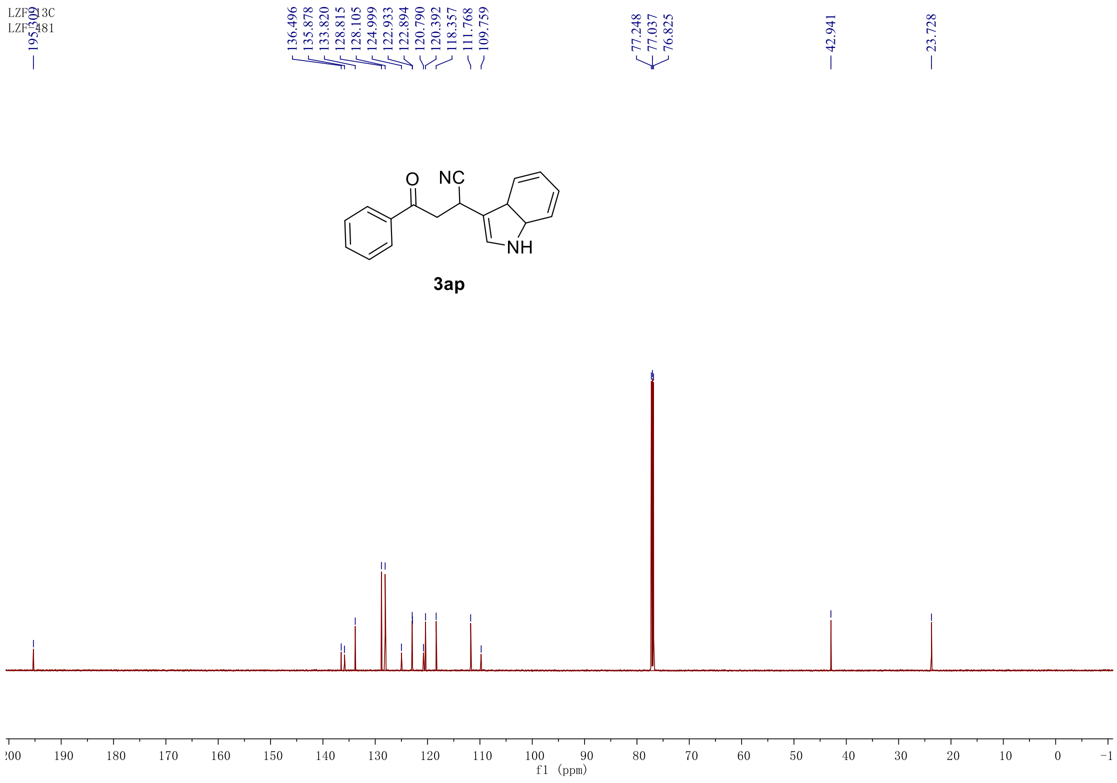

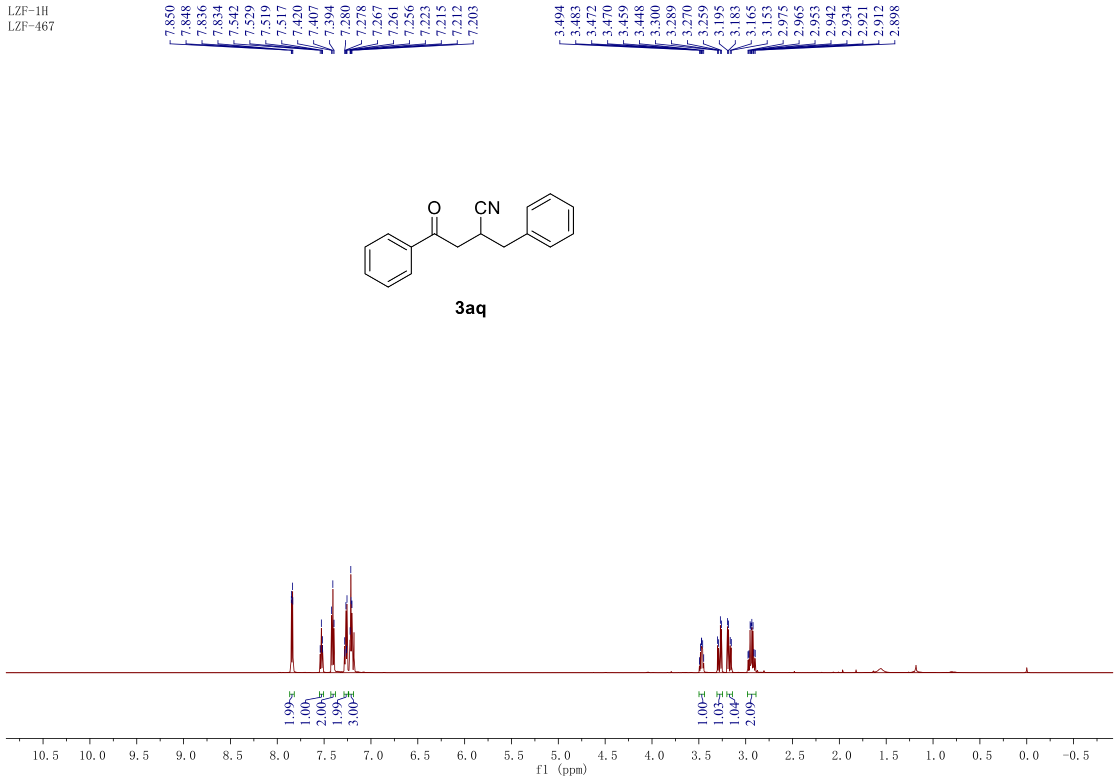

LZF-1H
LZF-467
7.850 7.848 7.836 7.834 7.542 7.529 7.519 7.517 7.420 7.407 7.394 7.280 7.278 7.267 7.261 7.256 7.223 7.215 7.212 7.203
3.494 3.483 3.472 3.470 3.459 3.448 3.300 3.289 3.270 3.259 3.195 3.183 3.165 3.153 2.975 2.965 2.953 2.942 2.934 2.921 2.912 2.898
O
CN
3aq
1.99 1.00 2.00 1.99 3.00
1.00 1.03 1.04 2.09
10.5 10.0 9.5 9.0 8.5 8.0 7.5 7.0 6.5 6.0 5.5 5.0 4.5 4.0 3.5 3.0 2.5 2.0 1.5 1.0 0.5 0.0 -0.5
f1 (ppm)

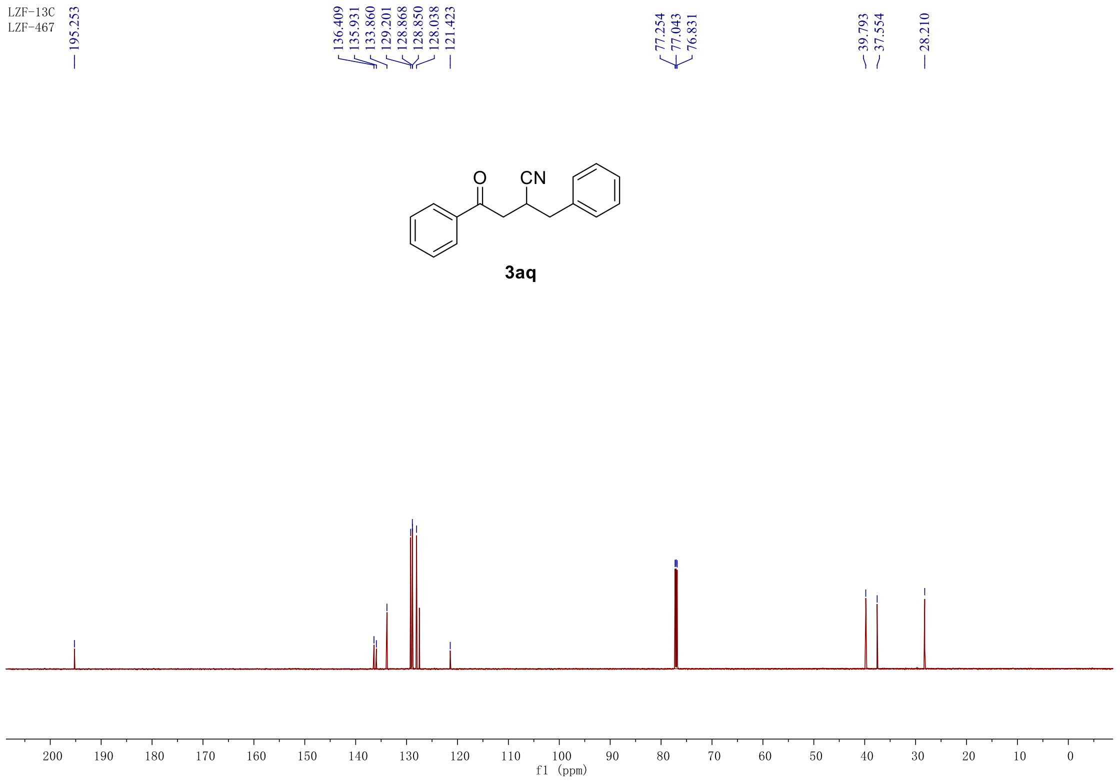

LZF-13C
LZF-467
195.253
136.409 135.931 133.860 129.201 128.868 128.850 128.038 121.423
77.254 77.043 76.831
39.793 37.554
28.210
O
CN
3aq
200 190 180 170 160 150 140 130 120 110 100 90 80 70 60 50 40 30 20 10 0
f1 (ppm)

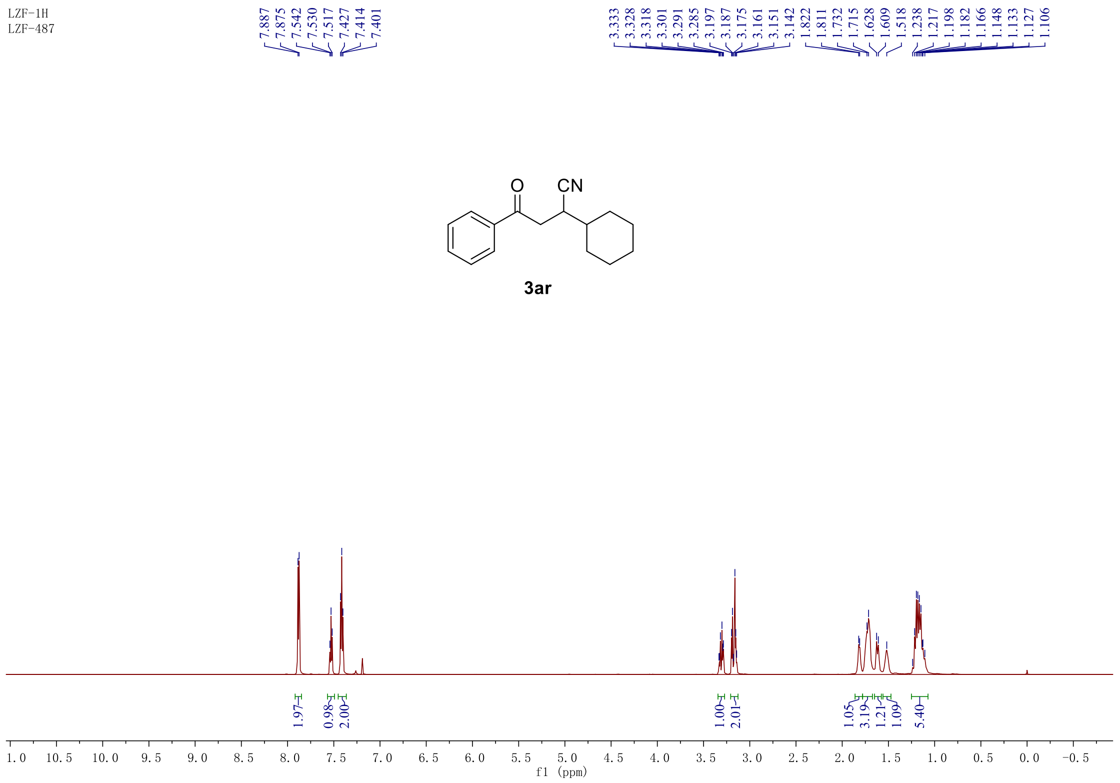
LZF-1H
LZF-487
7.887
7.875
7.542
7.530
7.517
7.427
7.414
7.401
3.333
3.328
3.318
3.301
3.291
3.285
3.197
3.187
3.175
3.161
3.151
3.142
1.822
1.811
1.732
1.715
1.628
1.609
1.518
1.238
1.217
1.198
1.182
1.166
1.148
1.133
1.127
1.106
O
CN
3ar
1.97
0.98
2.00
1.00
2.01
1.05
3.19
1.21
1.09
5.40
f1 (ppm)

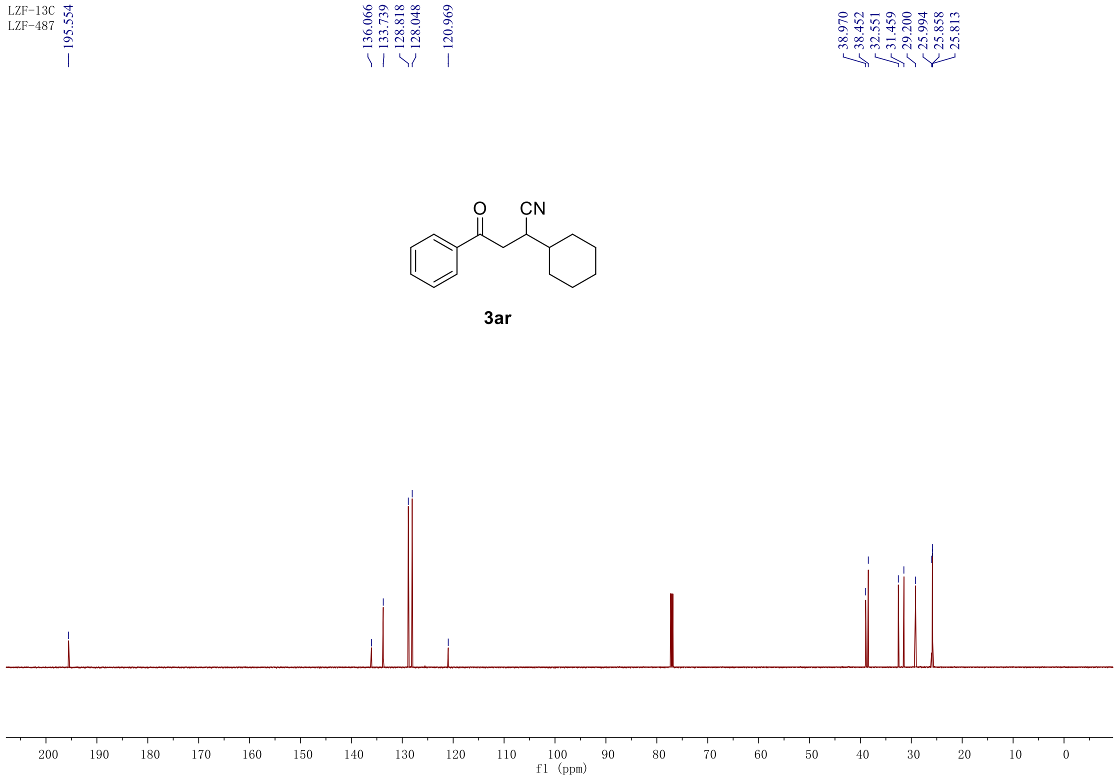
LZF-13C
LZF-487
195.554
136.066
133.739
128.818
128.048
120.969
38.970
38.452
32.551
31.459
29.200
25.994
25.858
25.813
O
CN
3ar
f1 (ppm)

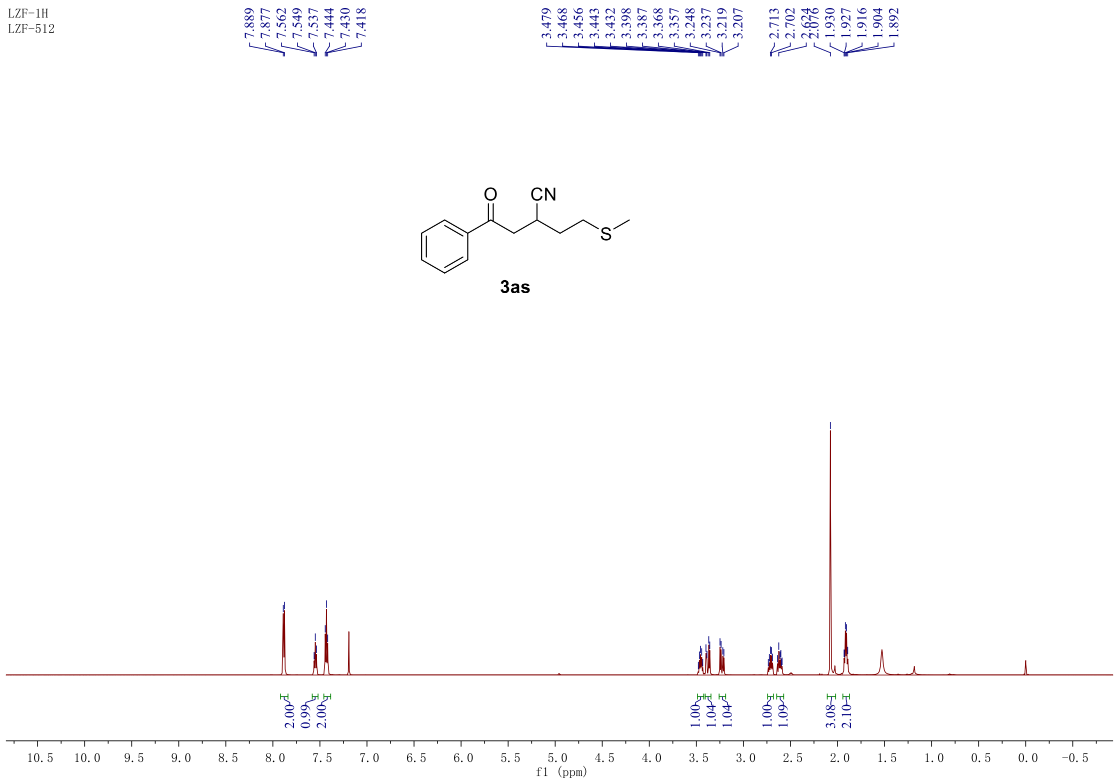
LZF-1H
LZF-512
7.889
7.877
7.562
7.549
7.537
7.444
7.430
7.418
3.479
3.468
3.456
3.443
3.432
3.398
3.387
3.368
3.357
3.248
3.237
3.219
3.207
2.713
2.702
2.624
2.076
1.930
1.927
1.916
1.904
1.892
O
CN
S
3as
2.00
0.99
2.00
1.00
1.04
1.04
1.00
1.09
3.08
2.10
10.5 10.0 9.5 9.0 8.5 8.0 7.5 7.0 6.5 6.0 5.5 5.0 4.5 4.0 3.5 3.0 2.5 2.0 1.5 1.0 0.5 0.0 -0.5
f1 (ppm)

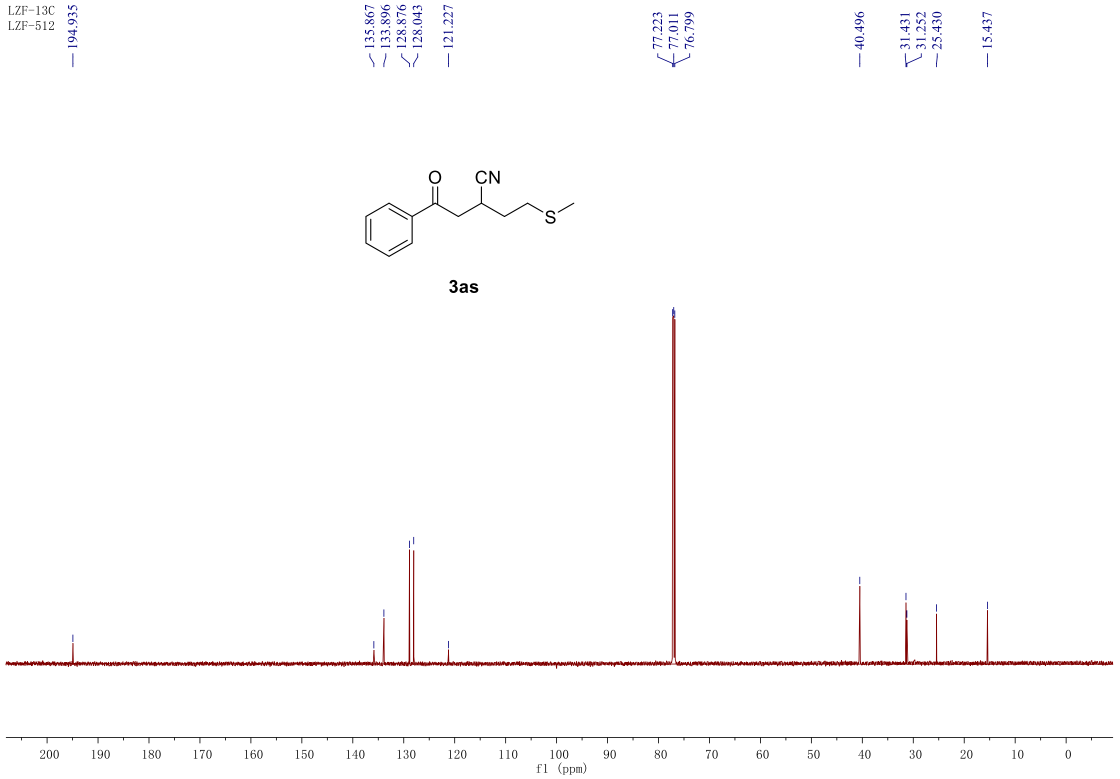
LZF-13C
LZF-512
194.935
135.867
133.896
128.876
128.043
121.227
77.223
77.011
76.799
40.496
31.431
31.252
25.430
15.437
O
CN
S
3as
200 190 180 170 160 150 140 130 120 110 100 90 80 70 60 50 40 30 20 10 0
f1 (ppm)

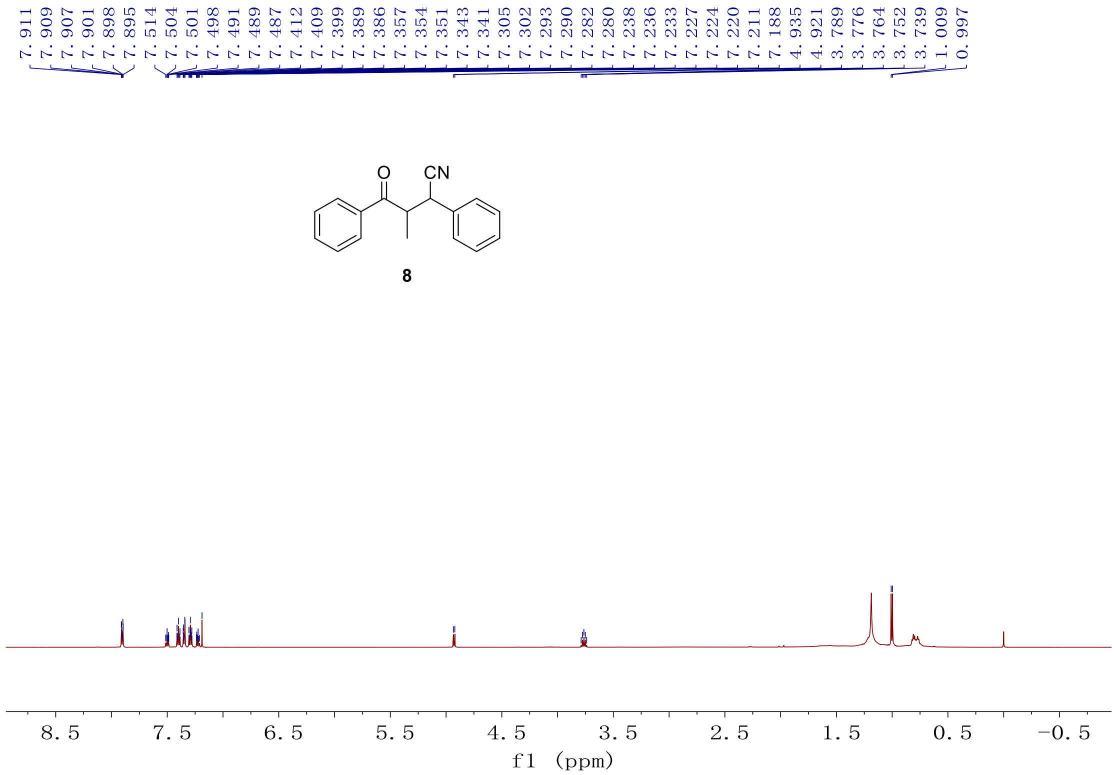
7.911 7.909 7.907 7.901 7.898 7.895 7.514 7.504 7.501 7.498 7.491 7.489 7.487 7.412 7.409 7.399 7.389 7.386 7.357 7.354 7.351 7.343 7.341 7.305 7.302 7.293 7.290 7.282 7.280 7.238 7.236 7.233 7.227 7.224 7.220 7.211 7.188 4.935 4.921 3.789 3.776 3.764 3.752 3.739 1.009 0.997
O
CN
8
8.5 7.5 6.5 5.5 4.5 3.5 2.5 1.5 0.5 -0.5
f1 (ppm)

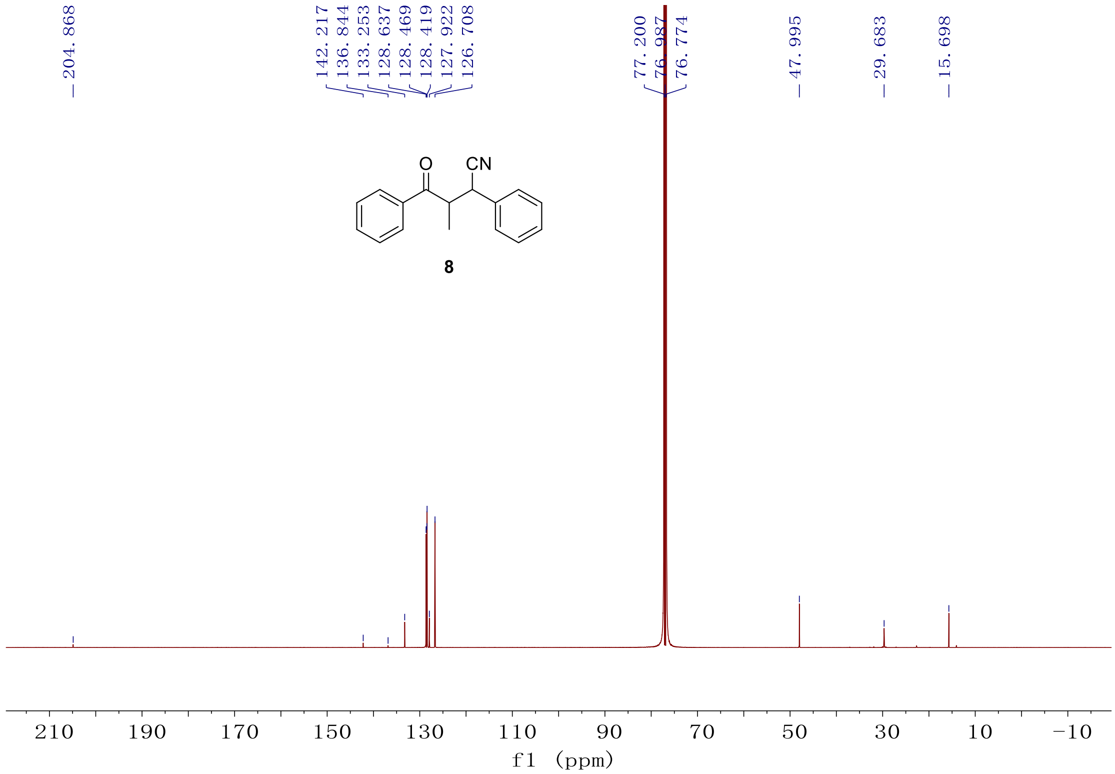
204.868
142.217 136.844 133.253 128.637 128.469 128.419 127.922 126.708
77.200 76.987 76.774
47.995
29.683
15.698
O
CN
8
210 190 170 150 130 110 90 70 50 30 10 -10
f1 (ppm)

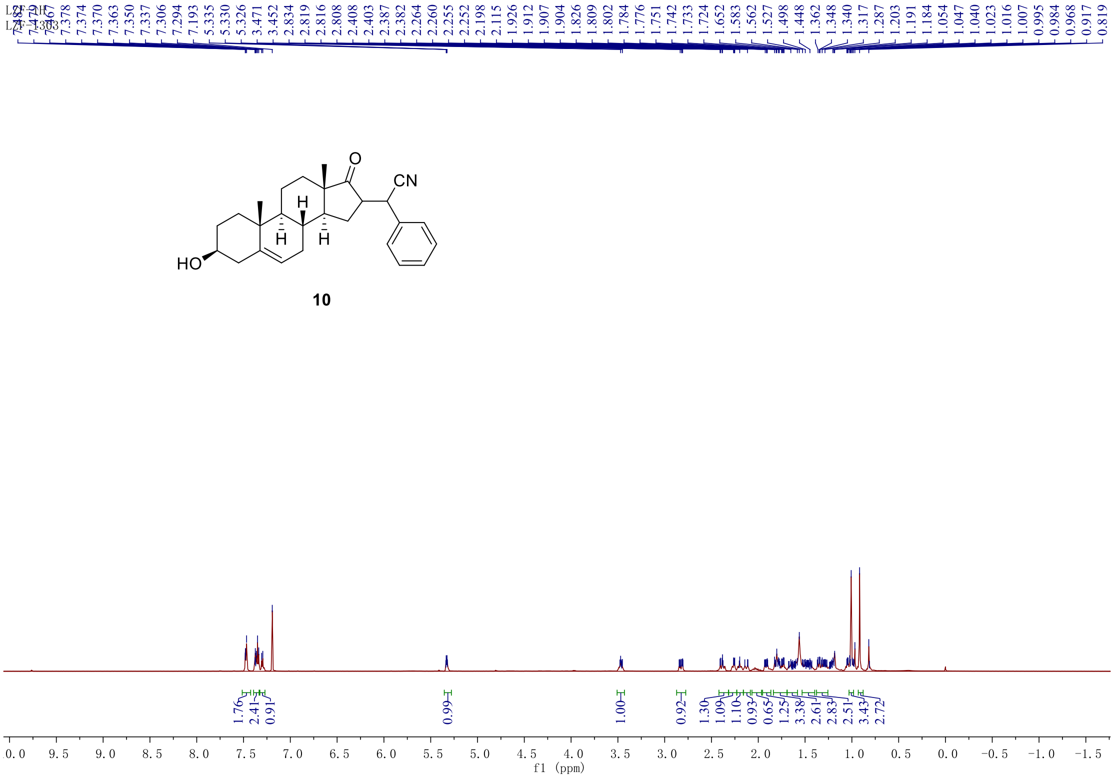
7.467
7.378
7.374
7.370
7.363
7.350
7.337
7.306
7.294
7.193
5.335
5.330
5.326
3.471
3.452
2.834
2.819
2.816
2.808
2.408
2.403
2.387
2.382
2.264
2.260
2.255
2.252
2.198
2.115
1.926
1.912
1.907
1.904
1.826
1.809
1.802
1.784
1.776
1.751
1.742
1.733
1.724
1.652
1.583
1.562
1.527
1.498
1.448
1.362
1.348
1.340
1.317
1.287
1.203
1.191
1.184
1.054
1.047
1.040
1.023
1.016
1.007
0.995
0.984
0.968
0.917
0.819
CN
O
H
H
H
HO
10
1.76
2.41
0.91
0.99
1.00
0.92
1.30
1.09
1.10
0.93
0.65
1.25
3.38
2.61
2.83
2.51
3.43
2.72
f1 (ppm)

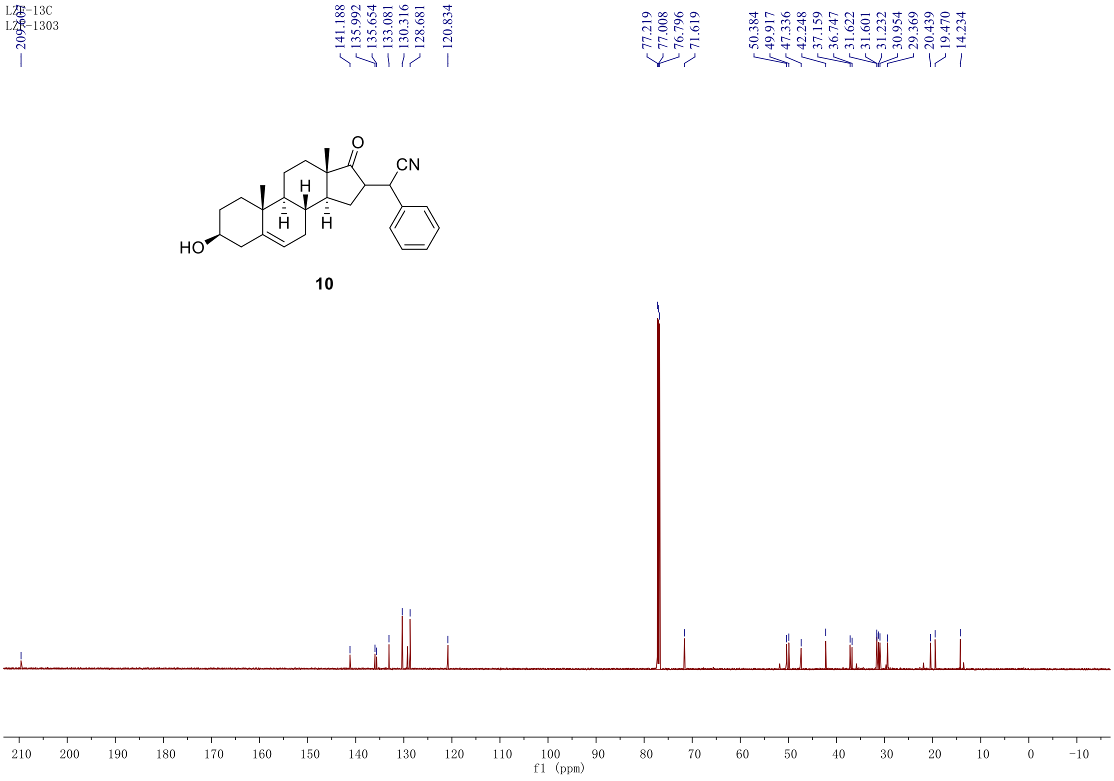
LZF-13C
LZF-1303
141.188
135.992
135.654
133.081
130.316
128.681
120.834
77.219
77.008
76.796
71.619
50.384
49.917
47.336
42.248
37.159
36.747
31.622
31.601
31.232
30.954
29.369
20.439
19.470
14.234
CN
O
H
H
H
HO
10
f1 (ppm)
